# Familial risks of five types of osteoarthritis in first-, second- and third-degree relatives - A nationwide Swedish family study

**DOI:** 10.1016/j.ocarto.2025.100637

**Published:** 2025-05-30

**Authors:** Christian Anker-Hansen, MirNabi Pirouzifard, Jan Sundquist, Kristina Sundquist, Bengt Zöller

**Affiliations:** Center for Primary Health Care Research, Lund University/Region Skåne, Malmö, Sweden

**Keywords:** Heredity, Epidemiology, Risk factors, Osteoarthritis, Family

## Abstract

**Objective:**

Osteoarthritis (OA) is a common degenerative disease with a genetic contribution. However, no large nationwide family study concerning the heredity of OA has been published. This first nationwide study aimed to determine the familial risks of the main types of OA in twins, full-siblings, half-siblings, and cousins in Sweden.

**Design:**

The Swedish Multigeneration register was linked to the National Patient Register (NPR) to investigate the heredity of OA (poly-, hip-, knee-, first carpometacarpal joint-, and other-OA) between 1997 and 2018. Offspring born by Swedish parents were included. The adjusted familial hazard ratios (HRs) with 95 ​% confidence interval (CI) were determined for OA among twins, full-siblings, half-siblings, and cousins. Adjustments were made for birth year, sex, educational level, and comorbidities (chronic obstructive pulmonary disease, alcoholism, and obesity).

**Results:**

A total of 6 547 966 individuals (48.77 ​% women) were included with mean age 41.44 years (range 0–86.96 years) at end of follow-up. Familial HRs were increased for all five types of OA (even in cousins) and correlated to degree of genetic resemblance between relatives. For instance, adjusted HRs among full-siblings were for poly-OA 2.29 (95 ​% CI 2.09–2.51), hip-OA 2.04 (95 ​% CI 1.98–2.07), knee-OA 1.75 (95 ​% CI 1.73–1.77), thumb-OA 2.60 (95 ​% CI 2.45–2.76), and other-OA 1.52 (95 ​% CI 1.48–1.56).

**Conclusions:**

Heredity is an important predictor of future risk of OA for all five types of OA in the Swedish population. Strongest heredity was observed for first carpometacarpal joint -OA followed by poly-OA and hip-OA. Weakest heredity was observed for knee-OA and other-OA.

## 1 Introduction

Osteoarthritis (OA) is a multifactorial, progressively deteriorating disease of the joints that causes cartilage degradation, joint space narrowing, and chronic synovitis of the joint [1]. The clinical manifestations vary but include stiffness, pain at rest, and, when weight-bearing, impaired function and recurring swelling [1]. OA is rare in young adults but increases with age and is considered an age-related disease [1]. However, OA is often underdiagnosed in younger individuals [2]. Occupation, especially repetitive heavy work, a sedative lifestyle as well as obesity are risk factors that can promote the onset of OA, as well as significant trauma to the joint, i.e. post-traumatic OA [3]. Women also have a higher risk of OA than men in all joints, especially after menopause, and oestrogen deficiency could be a possible explanation [[4], [5], [6]]. However, these risk factors do not fully explain an individual's risk of developing the disease as well as the progression of OA [7].

Several studies have indicated that hereditary factors are important regarding OA [7]. The heritability, i.e. the proportion of variation in a population that can be explained by genetic factors, of OA is reported to be between 35 ​% and 65 ​% [8,9]. Epidemiological studies have estimated that both non-genetic and genetic risk factors are also of importance for OA [10]. Twin studies have shown a heritability between 39 ​% and 65 ​% in radiographic OA of the hand and knee in women, about 60 ​% in hip OA, and about 70 ​% in OA of the spine [5,11,12]. However, in a Danish twins study of primary knee OA patients, no genetic component was found in men, and only a weak genetic component was found in women [13]. In another Danish twin study, the heritability for hip OA was 47 ​% [14]. The genetic influence increased from 50 years of age [14]. In a sibling study from the UK, the odds ratios (OR) in siblings with knee OA were 2.9 (95%CI 2.3–3.7) for tibiofemoral OA, and 1.7 (95%CI 1.4–2.2) for patellofemoral OA [15]. The estimated OR for knee OA was 2.9 and the heritability was 62 ​% [15]. A Dutch study of 191 middle-aged siblings with multiple joint OA found familial aggregation of osteoarthritis for hand with familial OR of 4.4 (95%CI 2.0–9.5), and OR for hip of 3.9 (95%CI 1.8–8.4). However, knee OA did not cluster in families [16].

Boer et al. performed a multicohort genome-wide association study (GWAS) that identified a total of 100 independently associated risk variants across 11 different OA phenotypes [17]. The study showed that certain OA risk variants are common in disease development in any joint, but also the existence of joint-specific variants [17].

The present Swedish study is the first nationwide family study to determine familial risk for five types of OA (poly-, hip-, knee-, thumb- and other) among twins, full-siblings, half-siblings, and cousins, i.e. first-, second-, and third-degree relatives.

## 2 Method

### 2.1 Study population

We used several nationwide Swedish registers in this study [[18], [19], [20], [21], [22], [23]]. The Swedish Population Register was linked to the Multigeneration Register to identify parents, twins (no information about zygosity), full-siblings, half-siblings, and cousins. The Swedish Multigenerational Register provides reliable data on index cases born from January 1, 1932, and later. The follow-up time was until December 31, 2018. Linkage was also made to the National Patient Register (NPR), including all hospital discharge diagnoses in Sweden from 1997 onwards. It also includes hospital outpatient diagnoses from 2001 to 2018 but does not include diagnoses from primary health care. Thus, the study only includes data on specialist-treated OA patients born between 1932 and 2018 who were diagnosed from 1997 onwards. The International Classification of Disease 10th edition (ICD-10) started on January 1, 1997, and data on OA diagnoses were collected from this date.

Inclusion criteria were individuals born in Sweden to Swedish-born parents. Thus, both biological parents were obligatorily known to secure details on kinship. Exclusion criteria were individuals not born in Sweden or with one or both parents born outside of Sweden. Individuals born before January 1st, 1932, were also excluded as kinship is not defined for these individuals.

Four groups of relative pairs could be defined: twins (born on same date), full-siblings, half-siblings, and cousins [24]. We allowed the same person to be included in more than one family relationship and more than one type of OA. The ambiguity as to which relative's trait should be used as the dependent, and which as the independent variable, is frequently resolved by using double entry [[24], [25], [26]]. In the analyses, each relative was entered twice - once as the first relative (dependent variable) and once as the second relative in a pair (explanatory variable or predictor for relative history of OA). In an additional analysis, parent-offspring pairs were also included. Parent-offspring pairs were conducted as single entry. Only parent-offspring pairs with both parents alive in 1997 or later were included in this analysis. This means that, with the exception of parent-offspring pairs, each relative is entered twice in the data, and each member of a relative pair provides once the dependent and once the explanatory variable as described [24]. While the consistency of the regression estimates for heritability and environmental influences is not affected by double entry, the standard errors of the coefficients are biased and need to be adjusted [[24], [25], [26]]. In the Swedish Population Register, we identified all relative pairs, i.e. 148 758 double-entry twins, 9 989 446 double-entry full-siblings, 2 839 076 double entry half-siblings and 25 883 163 double entry cousins (Supplementary Table 1). All possible relative pairs were ascertained in a family. Robust standard errors were used to consider non-independence between cases [24,27].

Baseline for study participants was defined as the date of inclusion, i.e., January 1st, 1997, or date of birth. Follow-up time was the time from baseline to event of OA, emigration, death, or end of follow-up, whichever came first. Predictor was relative history of respective OA type. The outcome was the same type of OA diagnosis in parent-offspring, twins, full-siblings, half siblings, and cousins, i.e., first-, second-, and third-degree relatives.

### 2.2 Definition of OA

The five types of OA were defined by ICD-10 in the NPR between 1997 and 2018: poly osteoarthritis (M15), hip osteoarthritis (M16), knee osteoarthritis (M17), Osteoarthritis of first carpometacarpal joint (M18), and other osteoarthritis (M19) [18].

### 2.3 Main predictors

Main predictors (i.e. exposure) were OA (poly osteoarthritis, hip osteoarthritis, knee osteoarthritis, osteoarthritis of first carpometacarpal joint, or other osteoarthritis) in the proband in pairs of twins, full-siblings, half-siblings, and cousins.

### 2.4 Outcomes

Main outcomes (dependent variables) were OA (poly osteoarthritis, hip osteoarthritis, knee osteoarthritis, osteoarthritis of first carpometacarpal joint, or other osteoarthritis) in the relative of the proband in pairs of twins, full-siblings, half-siblings, and cousins.

### 2.5 Adjusting variables

Birth year was defined as the year of birth in the Swedish Population Register. Sex was defined as male or female. Education level was defined as level 1 ​= ​primary school only (9 years in Sweden) or missing value, level 2 ​= ​10–11 years, or level 3 ​= ​more than 11 years of education.

Comorbidities used for adjustment were by ICD-10 codes in the NPR: chronic obstructive pulmonary disease (COPD) (J41-J44), alcoholism (F10), obesity (E66), and cancer (C00–C97) [4,28,29]. Use of opioids was defined by prescription of opioids any time during follow-up with the Anatomical Therapeutic Chemical code (ATC code) NO2A in the Swedish prescribed drug register [23]. Occupation was classified into six categories: white collar jobs requiring advanced education (reference), blue collar jobs, administrative jobs, service-, care- and sales occupations, military jobs and other jobs requiring only shorter training.

### 2.6 Statistical analysis

Familial risks were defined by incidence rate ratios or hazard ratios (HR) and were calculated for subjects with relatives affected by OA compared with subjects whose relatives were not affected by OA. Incidence rates for OA were defined as the number of events divided by the person-time at risk. The familial incidence ratio between two incidence densities (rate in the exposed population divided by rate in those unexposed) gave the IRR. OA-free survival curves were constructed according to the Kaplan–Meier method to compare individuals with and without relative history of respective OA. For comparison of two curves, the log-rank test, resulting in a test statistic with a χ^2^ distribution and 1 *df*, was used. Cumulative incidence curves were also plotted. The adjusted familial HRs with a confidence interval (CI) of 95 ​% between twins, full-siblings, half siblings, and cousins' OA events were estimated using competing-event analysis according to Fine and Gray [30]. Death and emigration were competing events. Models were adjusted for year of birth, sex, level of education, COPD, alcoholism, and obesity. Familial HRs for OA were calculated for relatives of individuals who had a diagnosis of OA compared with relatives of individuals unaffected by OA as the reference group. An aggregated model with twins, full-siblings, half-siblings, and cousins was also created. Odds ratios (ORs) were compared with HRs estimated with either Cox regression or Competing risk models. Statistical significance was set at *P* ​< ​0.05, and all tests were two-tailed. Data were analysed using SAS version 9.4 (SAS Institute, USA).

## 3 Results

### 3.1 Descriptives

A total of 6,547,966 individuals in the Swedish population met the inclusion criteria (Table 1). In the whole study population, 51.23 ​% were males and 48.77 ​% were females, with a mean age of 41.44 years. Lower levels of educational attainment were observed among all types of OA compared to controls (Table 1). Supplementary Tables 1–4 show the descriptives for twins, full-siblings, half-siblings, and cousins separately.

**Table 1:** Characteristics of all study participants in the whole study sample (unique individuals) grouped by the presence of poly osteoarthritis, hip osteoarthritis, knee osteoarthritis, osteoarthritis of first carpometacarpal joint, and other osteoarthritis.

	All individuals n ​= ​6547966 (100 ​%)	Poly osteoarthritis (M15)		Hip osteoarthritis (M16)		Knee osteoarthritis (M17)		Osteoarthritis of first carpometacarpal joint (M18)		Other osteoarthritis (M19)	
		No M15 n ​= ​6525803 (99.66)	M15 n ​= ​22163 (0.34)	No M16 n ​= ​6420683 (98.06)	M16 n ​= ​127283 (1.94)	No M17 n ​= ​6314387 (96.43)	M17 n ​= ​233579 (3.57)	No M18 n ​= ​6511082 (99.44)	M18 n ​= ​36884 (0.56)	No M19 n ​= ​6418144 (98.02)	M19 n ​= ​129822 (1.98)
**Sex**											
Male	3354652 (51.23)	3349637 (51.33)	5015 (22.63)	3294442 (51.31)	60210 (47.30)	3239549 (51.30)	115103 (49.28)	3345482 (51.38)	9170 (24.86)	3292709 (51.30)	61943 (47.71)
Female	3193314 (48.77)	3176166 (48.67)	17148 (77.37)	3126241 (48.69)	67073 (52.70)	3074838 (48.70)	118476 (50.72)	3165600 (48.62)	27714 (75.14)	3125435 (48.70)	67879 (52.29)
**Education**											
>11y	3005031 (45.89)	2998167 (45.94)	6873 (31.01)	2969534 (46.25)	35590 (27.96)	2938378 (46.53)	66947 (28.66)	2995306 (46.00)	9738 (26.40)	2970362 (46.28)	34987 (26.95)
**Year of birth**											
Mean (SD)	1976.53 (23.22)	1976.63 (23.19)	1948.30 (8.91)	1977.09 (23.05)	1948.11 (10.58)	1977.46 (23.03)	1951.51 (11.35)	1976.68 (23.19)	1950.41 (9.73)	1976.99 (23.16)	1953.80 (12.49)
Median (IQR)	1976 (1957–1995)	1976 (1957–1996)	1947 (1942–1953)	1977 (1958–1996)	1946 (1940–1954)	1977 (1959–1996)	1950 (1943–1959)	1976 (1957–1996)	1949 (1943–1956)	1977 (1958–1996)	1952 (1944–1962)
(Range)	(1932–2018)	(1932–2018)	(1932–2010)	(1932–2018)	(1935–2017)	(1932–2018)	(1932–2015)	(1932–2018)	(1935–2017)	(1932–2018)	(1932–2013)
**Age at end follow-up**											
Mean (SD)	41.44 (22.80)	41.35 (22.78)	61.68 (9.20)	40.88 (22.63)	62.27 (10.67)	40.51 (22.59)	58.58 (11.58)	41.30 (22.77)	59.89 (9.50)	40.98 (22.73)	57.70 (12.63)
Median (IQR)	42.29 (22.87–60.54)	42.12 (22.80–60.38)	61.90 (56.12–68.04)	41.46 (22.46–59.63)	63.53 (56.31–69.77)	40.80 (22.04–59.13)	59.70 (51.59–66.88)	41.96 (22.71–60.29)	60.21 (54.26–66.13)	41.54 (22.46–59.87)	59.07 (50.14–66.79)
(Range)	(0.00–86.96)	(0.00–86.96)	(0.41–85.38)	(0.00–86.96)	(0.05–86.68)	(0.00–86.96)	(0.54–86.62)	(0.00–86.96)	(0.46–86.28)	(0.00–86.96)	(0.08–86.60)
**Age at M onset**											
Mean (SD)	NA	NA	61.68 (9.20)	NA	62.27 (10.67)	NA	58.58 (11.58)	NA	59.89 (9.50)	NA	57.70 (12.63)
Median (IQR)	NA	NA	61.90 (56.12–68.04)	NA	63.53 (56.31–69.77)	NA	59.70 (51.59–66.88)	NA	60.21 (54.26–66.13)	NA	59.07 (50.14–66.79)
(Range)	NA	NA	(0.41–85.38)	NA	(0.05–86.68)	NA	(0.54–86.62)	NA	(0.46–86.28)	NA	(0.08–86.60)
**Comorbidities; %**											
COPD	1.67	1.66	6.42	1.58	6.24	1.57	4.50	1.64	6.82	1.60	5.31
Alcoholism	3.15	3.15	3.11	3.14	3.72	3.13	3.65	3.14	3.89	3.11	4.86
Obesity	2.96	2.95	5.55	2.88	6.98	2.76	8.40	2.94	5.97	2.88	6.97

Age at end of follow-up: death, migration, M15-M19 diagnosis or end of study period on December 31, 2018, whichever came first; NA, no applicable; SD, standard deviation; IQR, interquartile range.

ICD-10 codes: M15, poly osteoarthritis; M16 osteoarthritis of the hip joint; M17 osteoarthritis of the knee joint; M18 osteoarthritis of the thumb joint; M19 other types of osteoarthritis.

Out of this population, there were 22,163 patients with poly OA (ICD-10 ​= ​M15) Among poly OA patients, 77.37 ​% were females and the mean age was 61.68 years. A total of 127 283 patients had hip OA (ICD-10 ​= ​M16). Among hip OA patients, 52.70 ​% were females and the mean age was 62.27 years. 233,579 patients had knee OA (ICD-10 ​= ​M17) Among knee OA patients, 50.72 ​% were females and the mean age was 58.58 years. 36 884 patients had carpometacarpal thumb OA (ICD-10 ​= ​M18). Among thumb OA patients, 75.14 ​% were females and the mean age was 59.89 years. Lastly, 129,822 individuals had other OA (ICD-10 ​= ​M19) Among other OA patients, 5229 were females and the mean age was 57.70 years. (Table 1).

Obesity and COPD were more common among all types of OA (Table 1). Obesity was especially common among knee OA patients (8.40 ​%). The frequency of alcoholism was slightly higher among all OA groups except for poly OA (Table 1).

### 3.2 Kaplan-Meier analysis and cumulative incidence curves

The familial associations for the five studied types of OA are displayed using Kaplan-Meier analysis and cumulative incidence curves among twins, full-siblings, half-siblings, and cousins (Supplementary Fig. 1A-1E and 2A-2E). The Kaplan-Meier analysis and the cumulative incidence curves suggest a relationship between OA-free survival and the degree of genetic resemblance for all five types of OA (Supplementary Fig. 1A-1E and 2A-2E).

### 3.3 Familial risk of poly OA

**Fig. 1:**
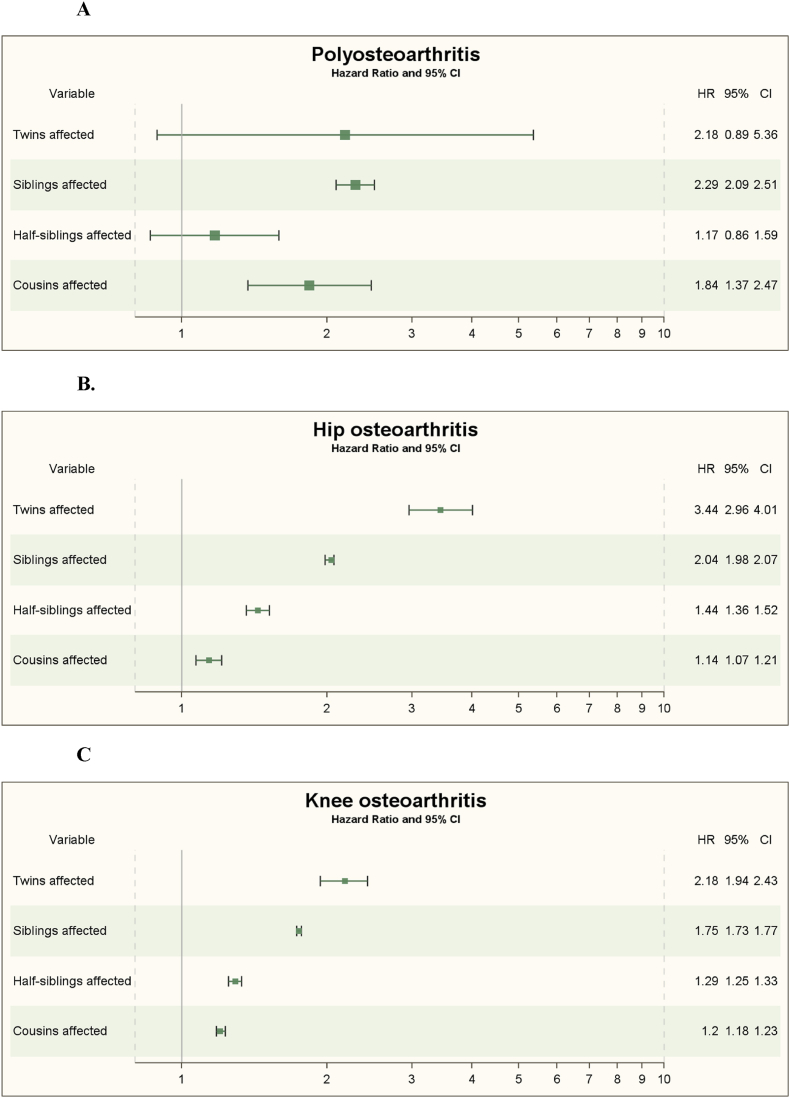

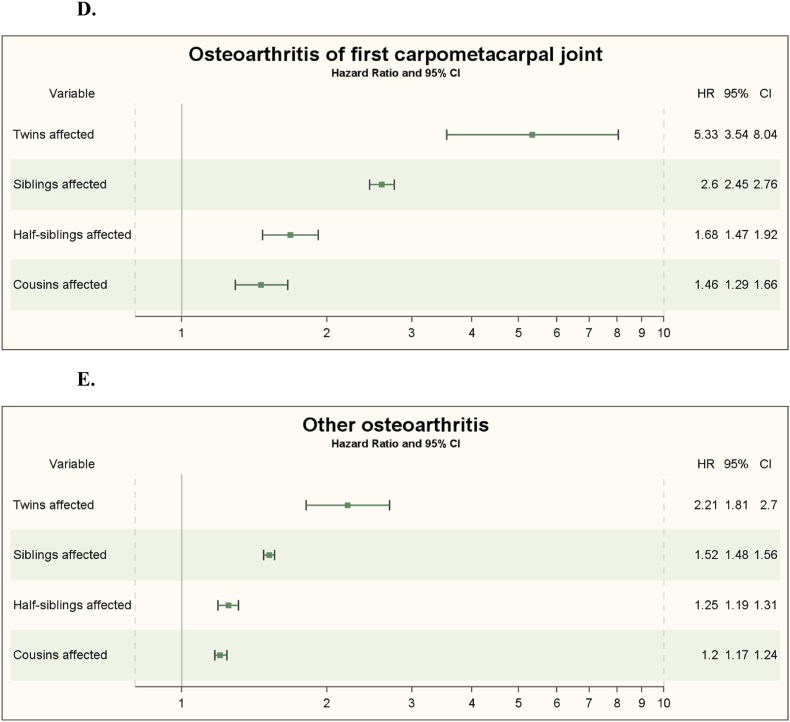
**A-E.** Forest plots of adjusted familial hazard ratios (HR) among twins, full-siblings, half-siblings, and cousins for: A) poly-osteoarthritis (ICD-10 ​= ​M15); B) hip osteoarthritis (ICD-10 ​= ​M16); C) knee osteoarthritis (ICD-10 ​= ​M17); D) thumb osteoarthritis (ICD-10 ​= ​M18); and E): other osteoarthritis (ICD-10 ​= ​M19). Abbreviations: HR ​= ​hazard ratio; CI ​= ​confidence interval).

Out of the 148,758 double-entry twins, 444 of them had the diagnosis poly OA with a fully adjusted familial HR for twins of 2.18 (95 ​% CI 0.89–5.36) (Fig. 1A and Table 2).

**Table 2:** Familial Hazard ratios (HRs) with 95 ​% confidence intervals (95 ​% CI) among twins, full-siblings, half-siblings and cousins for poly osteoarthritis (ICD-10 ​= ​M15) for individuals with relative history of poly osteoarthritis compared with those with non-affected relatives.

Variable	Person-years, No.	Cases, No./Persons at risk, No.	Incidence rate, cases/1000 person-years	Incidence rate ratio (95%CI)	HR(95 ​% CI)		
					Model 1	Model 2	Model 3
**Twins** (n ​= ​148758)							
Non-poly osteoarthritis	2667984	434/148314	0.16 (0.15–0.18)	1 [Reference]	1 [Reference]	1 [Reference]	1 [Reference]
Poly osteoarthritis	9043	10/444	1.11 (0.59–2.06)	**6.80∗∗∗** (3.63–12.72)	**6.27∗∗∗** (2.58–15.27)	2.21 (0.90–5.41)	2.18 (0.89–5.36)
**Full-siblings** (n ​= ​9989446)							
Non-poly osteoarthritis	193103424	42325/9946157	0.22 (0.21–0.22)	1 [Reference]	1 [Reference]	1 [Reference]	1 [Reference]
Poly osteoarthritis	885178	964/43289	1.09 (1.02–1.16)	**4.97∗∗∗** (4.66–5.30)	**4.73∗∗∗** (4.32–5.18)	**2.30∗∗∗** (2.30–2.53)	**2.29∗∗∗** (2.09–2.51)
**Half-siblings** (n ​= ​2839076)							
Non-poly osteoarthritis	54614769	6030/2832996	0.11 (0.11–0.11)	1 [Reference]	1 [Reference]	1 [Reference]	1 [Reference]
Poly osteoarthritis	127394	50/6080	0.39 (0.30–0.52)	**3.56∗∗∗** (2.69–4.70)	**3.37∗∗∗** (2.48–4.58)	1.17 (0.86–1.59)	1.17 (0.86–1.59)
**Cousins** (n ​= ​25845852)							
Non-poly osteoarthritis	494767943	15553/25830249	0.03 (0.03–0.03)	1 [Reference]	1 [Reference]	1 [Reference]	1 [Reference]
Poly osteoarthritis	336028	50/15603	0.15 (0.11–0.20)	**4.73∗∗∗** (3.58–6.24)	**4.42∗∗∗** (3.29–5.93)	**1.84∗∗∗** (1.37–2.47)	**1.84∗∗∗** (1.37–2.47)

HR, Hazard ratio. Model 1 crude. Model 2 adjusted for sex, education and birth year. Model 3 adjusted additionally for COPD, alcoholism and obesity.

All calculations were based on double entry. Significance levels: ∗p ​< ​0.05, ∗∗p ​< ​0.01, ∗∗∗p ​< ​0.001.

Out of the 9,989,446 double-entry full-siblings, 43,289 had the diagnosis poly OA with a fully adjusted familial HR for full-siblings of 2.29 (95 ​% CI 2.09–2.51) (Fig. 1A and Table 2).

Out of the 2,839,076 double-entry half-siblings, 6080 had the diagnosis poly OA with a fully adjusted familial HR for half-siblings of 1.17 (95 ​% CI 0.86–1.59) (Fig. 1A and Table 2).

Out of the 25,883,163 double-entry cousins, 15,623 had the diagnosis poly OA with a fully adjusted familial HR for cousins of 1.84 (95 ​% CI 1.37–2.47) (Fig. 1 and Table 2).

Fig. 1A shows the familial adjusted HRs of poly OA among twins, full-siblings, half-siblings, and cousins. There is no correlation to the degree of genetic resemblance in Fig. 1, but the numbers of familial cases are limited (Supplementary Tables 1–4).

### 3.4 Familial risk of hip OA

Out of the 148,758 double-entry twins, 2672 had the diagnosis hip OA with a fully adjusted familial HR for twins of 3.44 (95 ​% CI 2.99–4.01) (Fig. 1B and Table 3).

**Table 3:** Familial Hazard ratios (HRs) with 95 ​% confidence intervals (95 ​% CI) among twins, full-siblings, half-siblings and cousins for hip osteoarthritis (ICD-10 ​= ​M16) for individuals with relative history of hip osteoarthritis compared with those with non-affected relatives.

Variable	Person-years, No.	Cases, No./Persons at risk, No.	Incidence rate, cases/1000 person-years	Incidence rate ratio (95%CI)	HR(95 ​% CI)		
					Model 1	Model 2	Model 3
**Twins** (n ​= ​148758)							
Non-hip osteoarthritis	2609169	2214/146086	0.85 (0.82–0.88)	1 [Reference]	1 [Reference]	1 [Reference]	1 [Reference]
Hip osteoarthritis	50998	458/2672	8.98 (8.19–9.84)	**10.58∗∗∗** (9.57–11.70)	**9.89∗∗∗** (8.55–11.44)	**3.44∗∗∗** (2.96–4.00)	**3.44∗∗∗** (2.99–4.01)
**Full-siblings** (n ​= ​9989446)							
Non-hip osteoarthritis	187446050	224076/9737480	1.20 (1.19–1.20)	1 [Reference]	1 [Reference]	1 [Reference]	1 [Reference]
Hip osteoarthritis	4949965	27890/251966	5.63 (5.57–5.70)	**4.71∗∗∗** (4.66–4.77)	**4.50∗∗∗** (4.40–4.59)	**2.03∗∗∗** (1.99–2.08)	**2.04∗∗∗** (1.98–2.07)
**Half-siblings** (n ​= ​2839076)							
Non-hip osteoarthritis	53925161	29729/2807799	0.55 (0.55–0.56)	1 [Reference]	1 [Reference]	1 [Reference]	1 [Reference]
Hip osteoarthritis	643966	1548/31277	2.40 (2.29–2.53)	**4.36∗∗∗** (4.14–4.59)	**4.10∗∗∗** (3.87–4.33)	**1.44∗∗∗** (1.36–1.52)	**1.44∗∗∗** (1.36–1.52)
**Cousins** (n ​= ​25845852)							
Non-hip osteoarthritis	492222241	107482/25737214	0.22 (0.22–0.22)	1 [Reference]	1 [Reference]	1 [Reference]	1 [Reference]
Hip osteoarthritis	2327436	1156/108638	0.50 (0.47–0.53)	**2.28∗∗∗** (2.15–2.41)	**2.12∗∗∗** (1.99–2.25)	**1.14∗∗∗** (1.08–1.21)	**1.14∗∗∗** (1.07–1.21)

HR, Hazard ratio. Model 1 crude. Model 2 adjusted for sex, education and birth year. Model 3 adjusted additionally for COPD, alcoholism and obesity.

All calculations were based on double entry. Significance levels: ∗p ​< ​0.05, ∗∗p ​< ​0.01, ∗∗∗p ​< ​0.001.

Out of the 9,989,446 double-entry full-siblings, 251 966 had the diagnosis hip OA with a fully adjusted familial HR for full-siblings of 2.04 (95%CI 1.98–2.07) (Fig. 1B and Table 3).

Out of the 2,839,076 double-entry half-siblings, 31,277 had the diagnosis hip OA with a fully adjusted familial HR for half-siblings of 1.44 (95 ​% CI 1.36–1.52) (Fig. 1B and Table 3).

Out of the 25,883,163 double-entry cousins, 108,813 had the diagnosis hip OA with a fully adjusted familial HR for cousins of 1.14 (95 ​% CI 1.07–1.21) (Fig. 1B and Table 3).

Fig. 1B shows the familial fully adjusted HRs of hip OA among twins, full-siblings, half-siblings, and cousins. There is a correlation between familial HRs and the degree of genetic resemblance in Fig. 1B.

### 3.5 Familial risk of knee OA

Out of the 148,758 double-entry twins, 4771 had the diagnosis knee OA with a fully adjusted familial HR for twins of 2.18 (95 ​% CI 1.94–2.43) (Fig. 1C and Table 4).

**Table 4:** Familial Hazard ratios (HRs) with 95 ​% confidence intervals (95 ​% CI) among twins, full-siblings, half-siblings and cousins for knee osteoarthritis (ICD-10 ​= ​M17) for individuals with relative history of knee osteoarthritis compared with those with non-affected relatives.

Relative history of Knee osteoarthritis	Person-years, No.	Cases, No./Persons at risk, No.	Incidence rate, cases/1000 person-years	Incidence rate ratio (95%CI)	HR(95 ​% CI)		
					Model 1	Model 2	Model 3
**Twins** (n ​= ​148758)							
Non-knee osteoarthritis	2549152	3977/143987	1.56 (1.51–1.61)	1 [Reference]	1 [Reference]	1 [Reference]	1 [Reference]
Knee osteoarthritis	92495	794/4771	8.58 (8.01–9.20)	**5.50∗∗∗** (5.10–5.94)	**5.18∗∗∗** (4.65–5.78)	**2.22∗∗∗** (1.98–2.48)	**2.18∗∗∗** (1.94–2.43)
**Full-siblings** (n ​= ​9989446)							
Non-knee osteoarthritis	181979682	384024/9544258	2.11 (2.10–2.12)	1 [Reference]	1 [Reference]	1 [Reference]	1 [Reference]
Knee osteoarthritis	8718710	61164/445188	7.02 (6.96–7.07)	**3.32∗∗∗** (3.30–3.35)	**3.20∗∗∗** (3.16–3.25)	**1.78∗∗∗** (1.75–1.80)	**1.75∗∗∗** (1.73–1.77)
**Half-siblings** (n ​= ​2839076)							
Non-knee osteoarthritis	52832252	64218/2769566	1.22 (1.21–1.22)	1 [Reference]	1 [Reference]	1 [Reference]	1 [Reference]
Knee osteoarthritis	1420272	5292/69510	3.73 (3.63–3.83)	**3.07∗∗∗** (2.98–3.15)	**2.91∗∗∗** (2.82–3.01)	**1.30∗∗∗** (1.26–1.34)	**1.29∗∗∗** (1.25–1.33)
**Cousins** (n ​= ​25845852)							
Non-knee osteoarthritis	486025083	311936/25523500	0.64 (0.64–0.64)	1 [Reference]	1 [Reference]	1 [Reference]	1 [Reference]
Knee osteoarthritis	6860891	10416/322352	1.52 (1.49–1.55)	**2.37∗∗∗** (2.32–2.42)	**2.24∗∗∗** (2.20–2.30)	**1.21∗∗∗** (1.18–1.23)	**1.20∗∗∗** (1.18–1.23)

HR, Hazard ratio. Model 1 crude. Model 2 adjusted for sex, education and birth year. Model 3 adjusted additionally for COPD, alcoholism and obesity.

All calculations were based on double entry. Significance levels: ∗p ​< ​0.05, ∗∗p ​< ​0.01, ∗∗∗p ​< ​0.001.

Out of the 9,989,446 double-entry full-siblings, 445,188 had the diagnosis knee OA with a fully adjusted familial HR for full-siblings of 1.75 (95 ​% CI 1.73–1.77) (Fig. 1C and Table 4).

Out of the 2,839,076 double-entry half-siblings, 69,510 had the diagnosis knee OA with a fully adjusted familial HR for half-siblings of 1.29 (95 ​% CI 1.25–1.33) (Fig. 1C and Table 4).

Out of the 25,883,163 double-entry cousins, 322,766 had the diagnosis knee OA with a fully adjusted familial HR for cousins of 1.20 (95 ​% CI 1.18–1.23) (Fig. 1C and Table 4).

Fig. 1C shows the familial fully adjusted HRs of knee OA among twins, full-siblings, half-siblings, and cousins. There is a correlation to the degree of genetic resemblance in Fig. 1C.

### 3.6 Familial risk of thumb OA

Out of the 148,758 double-entry twins, 723 had the diagnosis thumb OA with a fully adjusted familial HR for twins of 5.33 (95 ​% CI 3.54–8.04) (Fig. 1D and Table 5).

**Table 5:** Familial Hazard ratios (HRs) with 95 ​% confidence intervals (95 ​% CI) among twins, full-siblings, half-siblings and cousins for osteoarthritis of first carpometacarpal joint (ICD-10 ​= ​M18) for individuals with relative history of osteoarthritis of first carpometacarpal joint compared with those with non-affected relatives.

Relative history of Knee osteoarthritis	Person-years, No.	Cases, No./Persons at risk, No.	Incidence rate, cases/1000 person-years	Incidence rate ratio (95%CI)	HR(95 ​% CI)		
					Model 1	Model 2	Model 3
**Twins** (n ​= ​148758)							
Non-thumb osteoarthritis	2660136	673/148035	0.26 (0.23–0.27)	1 [Reference]	1 [Reference]	1 [Reference]	1 [Reference]
Thumb osteoarthritis	14614	50/723	3.42 (2.59–4.51)	**13.52∗∗∗** (10.15–18.02)	**12.68∗∗∗** (8.42–19.08)	**5.35∗∗∗** (3.55–8.07)	**5.33∗∗∗** (3.54–8.04)
**Full-siblings** (n ​= ​9989446)							
Non-thumb osteoarthritis	192350100	67797/9919159	0.35 (0.35–0.36)	1 [Reference]	1 [Reference]	1 [Reference]	1 [Reference]
Thumb osteoarthritis	1435871	2490/70287	1.73 (1.67–1.80)	**4.92∗∗∗** (4.73–5.12)	**4.70∗∗∗** (4.42–4.99)	**2.62∗∗∗** (2.46–2.78)	**2.60∗∗∗** (2.45–2.76)
**Half-siblings** (n ​= ​2839076)							
Non-thumb osteoarthritis	54445111	12030/2826804	0.22 (0.22–0.22)	1 [Reference]	1 [Reference]	1 [Reference]	1 [Reference]
Thumb osteoarthritis	256542	242/12272	0.94 (0.83–1.07)	**4.27∗∗∗** (3.76–4.85)	**4.01∗∗∗** (3.51–4.58)	**1.69∗∗∗** (1.48–1.93)	**1.68∗∗∗** (1.47–1.92)
**Cousins** (n ​= ​25845852)							
Non-thumb osteoarthritis	494138525	39000/25806608	0.08 (0.08–0.08)	1 [Reference]	1 [Reference]	1 [Reference]	1 [Reference]
Thumb osteoarthritis	843345	244/39244	0.29 (0.26–0.33)	**3.66∗∗∗** (3.23–4.15)	**3.39∗∗∗** (2.99–3.84)	**1.48∗∗∗** (1.30–1.67)	**1.46∗∗∗** (1.29–1.66)

HR, Hazard ratio. Model 1 crude. Model 2 adjusted for sex, education and birth year. Model 3 adjusted additionally for COPD, alcoholism and obesity.

All calculations were based on double entry. Significance levels: ∗p ​< ​0.05, ∗∗p ​< ​0.01, ∗∗∗p ​< ​0.001.

Out of the 9,989,446 double-entry full-siblings, 70,287 had the diagnosis thumb OA with a fully adjusted familial HR for full-siblings of 2.60 (95 ​% CI 2.45–2.76) (Fig. 1D and Table 5).

Out of the 2,839,076 double-entry half-siblings, 12,272 had the diagnosis thumb OA with a fully adjusted familial HR for half-siblings of 1.68 (95 ​% CI 1.47–1.92) (Fig. 1D and Table 5).

Out of the 25 883 163 double-entry cousins, 39 307 had the diagnosis thumb OA with a fully adjusted familial HR for cousins of 1.46 (95 ​% CI 1.29–1.66) (Fig. 1D and Table 5).

Fig. 1D shows the familial adjusted HRs of thumb OA among twins, full-siblings, half-siblings, and cousins. There is a correlation between HR and the degree of genetic resemblance in Fig. 1D.

### 3.7 Familial risk of other OA

Out of the 148,758 double-entry twins, 2578 had the diagnosis other OA with a fully adjusted familial HR for twins of 2.21 (95 ​% CI 1.81–2.70) (Fig. 1E and Table 6).

**Table 6:** Familial Hazard ratios (HRs) with 95 ​% confidence intervals (95 ​% CI) among twins, full-siblings, half-siblings and cousins for other osteoarthritis (ICD-10 ​= ​M19) for individuals with relative history of other osteoarthritis compared with those with non-affected relatives.

Variable	Person-years, No.	Cases, No./Persons at risk, No.	Incidence rate, cases/1000 person-years	Incidence rate ratio (95%CI)	HR(95 ​% CI)		
					Model 1	Model 2	Model 3
**Twins** (n ​= ​148758)							
Non-other osteoarthritis	2610438	2360/146180	0.90 (0.87–0.94)	1 [Reference]	1 [Reference]	1 [Reference]	1 [Reference]
Other osteoarthritis	52393	218/2578	4.16 (3.64–4.75)	**4.60∗∗∗** (4.01–5.29)	**4.22∗∗∗** (3.47–5.14)	**2.22∗∗∗** (1.82–2.70)	**2.21∗∗∗** (1.81–2.70)
**Full-siblings** (n ​= ​9989446)							
Non-other osteoarthritis	187650766	230162/9743712	1.23 (1.22–1.23)	1 [Reference]	1 [Reference]	1 [Reference]	1 [Reference]
Other osteoarthritis	5007881	15572/245734	3.11 (3.06–3.16)	**2.54∗∗∗** (2.49–2.78)	**2.41∗∗∗** (2.35–2.47)	**1.53∗∗∗** (1.50–1.57)	**1.52∗∗∗** (1.48–1.56)
**Half-siblings** (n ​= ​2839076)							
Non-other osteoarthritis	53486905	45668/2791360	0.85 (0.85–0.86)	1 [Reference]	1 [Reference]	1 [Reference]	1 [Reference]
Other osteoarthritis	991484	2048/47716	2.07 (1.98–2.16)	**2.42∗∗∗** (2.31–2.53)	**2.27∗∗∗** (2.16–2.38)	**1.26∗∗∗** (1.20–1.32)	**1.25∗∗∗** (1.19–1.31)
**Cousins** (n ​= ​25845852)							
Non-other osteoarthritis	488775522	229812/25611020	0.47 (0.47–0.47)	1 [Reference]	1 [Reference]	1 [Reference]	1 [Reference]
Other osteoarthritis	5011114	5020/234832	1.00 (0.97–1.03)	**2.13∗∗∗** (2.07–2.19)	**1.99∗∗∗** (1.93–2.05)	**1.21∗∗∗** (1.18–1.25)	**1.20∗∗∗** (1.17–1.24)

HR, Hazard ratio. Model 1 crude. Model 2 adjusted for sex, education and birth year. Model 3 adjusted additionally for COPD, alcoholism and obesity.

All calculations were based on double entry. Significance levels: ∗p ​< ​0.05, ∗∗p ​< ​0.01, ∗∗∗p ​< ​0.001.

Out of the 9,989,446 double-entry full-siblings, 245,734 had the diagnosis other OA with a fully adjusted familial HR for full-siblings of 1.52 (95 ​% CI 1.48–1.56) (Fig. 1E and Table 6).

Out of the 2,839,076 double-entry half-siblings, 47,716 had the diagnosis other OA with a fully adjusted familial HR for half-siblings of 1.25 (95 ​% CI 1.19–1.31) (Fig. 1E and Table 6).

Out of the 25,883,163 double-entry cousins, 235,123 had the diagnosis other OA with a fully adjusted familial HR for cousins of 1.20 (95 ​% CI 1.17–1.24) (Fig. 1E and Table 6).

Fig. 1E shows the familial adjusted HRs of other OA among twins, full-siblings, half-siblings, and cousins. There is a correlation to the degree of genetic resemblance in Fig. 1E.

### 3.8 Stratified analysis among full-siblings

Sex and age-stratified analysis was performed among full-siblings. In the sex-stratified analysis of familial HRs among full-siblings, there were only minor differences between males and females for all types of OA (Supplementary Table 5). In the age-stratified analysis of familial hazard ratios among full-siblings stratified by birth year (median year ​= ​1969), the higher familial HRs were higher for younger than older individuals (Supplementary Table 6).

### 3.9 Additional analysis among full-siblings

Additional adjustments for cancer and opioid use did not change the results to any major degree (Supplementary Table 7). Moreover, the exclusion of patients with cancer or COPD with adjustment for opioid use did not change the results to any major degree (Supplementary Table 8). Adjustment for occupation did not change the familial risk to any major degree either (Supplementary Table 9). Analysis was also made with ascertainment of only the two oldest siblings in multiplex families using either double or single entry (Supplementary Tables 10 and 11). This did not change the results to any major degree.

### 3.10 Other additional analysis

Among twins, there was a large difference between crude and adjusted HRs for all types of OA. This was related to the adjustment for birth year for all types of OA (Supplementary Tables 12–16).

An aggregated analysis of all different relationships (twins, full-siblings, half-sibling, cousins) in the same model did not change the results to any major degree (Supplementary Table 17). Thus, the familial risks were independent. Moreover, for the same models, familial risks were calculated using logistic regression (OR), Cox regression (HR) or Competing risk models (HR) (Supplementary Table 17).

Parent-offspring risk was also calculated (Supplementary Table 18). The familial risks were not, to any major degree, different from the full-sibling risks.

## 4 Discussion

This first large nationwide study presents estimates for familial risks in first-, second-, and third-degree relatives for five major types of OA. The strongest aggregations of OA were observed in pairs of twins and full-siblings, whereas this association was attenuated among half-siblings and cousin pairs. This correlation between familial risk and genetic resemblance is typical for complex traits and suggests a genetic contribution to OA [31,32]. Significant familial associations in third-degree relatives, i.e. cousins, of affected individuals support that genetic factors influence familial aggregation since cousins usually do not share a household [31,32]. Moreover, full-siblings and twins share a household, which could not explain the higher familial risk among twins compared with full-siblings. Unfortunately, we have no information about the zygosity of the included twins. There were significant hereditary patterns in all studied OA diagnoses, thus confirming a familial background of OA [33]. This study shows that there is strong heredity in carpometacarpal thumb OA, in poly OA, and hip OA, but less heredity in knee and other types of OA. This correlates with previous studies concerning thumb carpometacarpal joint osteoarthritis [34,35]. Among Swedish twins, a strong genetic component for hand OA was shown with a heritability of 48–87 ​% [35]. There were no significant results in poly OA (M15) for twins and half-siblings after adjustments. This is likely due to that poly OA is rarer and not sufficient to reach significance among half-siblings. Thus, in poly OA, there was only significance in the adjusted model for full-siblings and cousins, but not for twins or half-siblings, although the Kaplan-Meier analysis was significant (Table 2). Knee OA is more susceptible to obesity, previous trauma, and work-related overuse, which may lower familial risk in knee OA [4]. Shared household environments may affect familial risk. Our analysis has been adjusted for educational attainment and therefore, indirectly, for physical workload, i.e. blue-collar versus white-collar work. Direct analysis with adjustments for occupation (including blue versus white-collar jobs) did not affect the results either, which indicates that the estimated familial risks are not to a major degree related to socioeconomical or occupational factors [3].

For many complex diseases, the average familial risk in first-degree relatives is around two as for OA in the present study [32]. In complex traits, the familial risk is also higher among younger individuals, as in the present study [36]. In the analysis of familial HRs among full-siblings stratified by birth year (median year ​= ​1969), especially high familial HRs were observed for young individuals for all types of OA (Supplementary Table 7). Though large genome-wide association studies (GWAS) have found many genetic variants associated with OA, the variance in the phenotype explained by all genetic variants (i.e. variant heritability) is much lower than results from family studies [34]. For instance, variant heritability End-Stage Hip Osteoarthritis was only 17.6 ​% [37]. In the present study, the high familial HRs in the general population therefore suggest that further genetic studies of OA might be worthwhile.

### 4.1 Strengths and limitations

The nationwide population-based design of the present study is an advantage because there is no selection bias as in case-control studies. Moreover, OA was defined by hospital diagnoses, thus eliminating reporting bias. The OA diagnoses came from the NPR, which is a reliable register since it included both hospital discharge diagnoses from 1997 onwards as well as specialist outpatient clinic diagnoses from 2001 onwards [20,23]. It is therefore likely that included patients had later-stage OA, when they needed to be referred from primary health care to specialised care at hospitals or specialist clinics. However, in genetics, it is an advantage to study the most severe cases, i.e. those referred to hospital as in the present study, because the genetic contribution is often higher [36]. In Sweden about 60 ​% of all patients with hip OA and 55 ​% of all patients with knee OA have radiographs to confirm their diagnosis when they begin first-line treatment with physiotherapy according to the Swedish Osteoarthritis Register Report 2022 [38]. However, in the Swedish Osteoarthritis Register, mostly earlier stages of OA is registered, suggesting that a higher percentage of OA patients in our studied material of late stage OA had radiologically confirmed OA diagnosis. A limitation is that we could not confirm radiographically diagnosed OA, nor include early stages from primary care.

Compared with a Cox model, the Fine-Gray competing risk model takes competing risks into account, which provides a better estimation for the risk of the main outcome of interest when one or more competing risks are presented, such as death [30]. However, in the present study, the results were similar when using logistic regression, Cox regression, and Fine-Gray competing risk modelling, probably due to the large nationwide study design.

The prevalence numbers for all types of OA in this study ranged from 0.34 to 3.57 ​%, which are low, but this is related to that the study only included OA patients that were identified in the National Patient Register [20,23]. The global prevalence of radiographically confirmed knee OA in 2010 was estimated to be 3.8 ​% (95 ​% CI 3.6–4.1) and the global prevalence of radiographically confirmed hip OA in 2010 was 0.85 ​% (95 ​% CI 0.74–1.02), which, however, correlates with our prevalence numbers [39]. The present study population is young because only individuals born in 1932 or later with both biological parents identified were included in the study. The coverage of OA might therefore be high. Moreover, in genetics, it is an advantage to study younger cases because the genetic contribution is often higher, though a potential source of error is that OA is often underdiagnosed in young people [2,36].

A medical diagnosis of obesity was overrepresented among all OA groups but was especially prevalent among knee OA patients. Obesity is the main modifiable risk factor for OA [40]. Hip OA patients were the oldest, with a mean age of 62.27 years. The youngest OA group were those with M19 (other type) with a mean age of 57.70 years. Women were particularly prone to develop poly OA, (77.4 ​%) and thumb OA (75.1 ​%). Women also had more OA in general: hip OA (52.7 ​%), knee OA (50.7 ​%), and other OA (52.3 ​%). This is consistent with previous studies, which is a strength [4]. In the analysis of familial HRs among full-siblings stratified by sex only minor differences were observed between males and females for all types of OA (Supplementary Table 5). COPD was overrepresented among all types of OA, suggesting that severe smoking might harm the joints [41].

In conclusion, the present nationwide family study confirms that OA is a complex trait, although the genetic contribution varies between the different types of OA. Family history is an important risk factor for OA in the general population in Sweden. Information about the individual's future risk of OA may be of importance for the prevention of modifiable risk factors in predisposed individuals.

## Author contributions

CAH: Study design, statistical analysis, and writing and editing the manuscript, JS: Writing and editing the manuscript. KS: Study design, writing and editing the manuscript, and funding. MN: Study design, statistical analysis, writing, editing manuscript, and funding. BZ: Study design, statistical analysis, writing and editing manuscript, and funding.

## Ethical approval

The regional ethical committee at Lund University approved the study (approval nos. 2012/95 and later amendments) and no informed consent was needed. All the protocols were conducted in accordance with the Helsinki Declaration and the Data Registry Inspection in Stockholm.

## Declaration of Generative AI in scientific writing

The authors declare that they have not used generative AI in the writing of the article.

## Role of the funding source

None.

## Declaration of competing interest

The authors declare that they have no competing interests.
