# Supplementary material for: Familial risks of five types of osteoarthritis in first-, second- and third-degree relatives - A nationwide Swedish family study
Source: Osteoarthr Cartil Open. 2025 May 30;7(3):100637. doi: 10.1016/j.ocarto.2025.100637 (PMC12172947; doi:10.1016/j.ocarto.2025.100637)
Supplement: Multimedia component 1 [file mmc1.docx]

**Supplementary Tables article: Familial risks of five types of osteoarthritis in first-, second- and third-degree relatives - a nationwide Swedish family study.**

Anker-Hansen C, Pirouzifard M, Sundquist J, Sundquist K, Zöller B.

**Supplementary Tables 1-18**

**Supplementary Figures 1A-E**

**Supplementary Figures 2A-E**

| **Supplementary Table 1. TWINS. Characteristics of patients in the twin study sample (unique individuals) for poly osteoarthritis (M15), hip osteoarthritis (M16), knee osteoarthritis (M17), osteoarthritis of first carpometacarpal joint (M18), and other osteoarthritis (M19), respectively.** | | | | | | | | | | | |  |
| --- | --- | --- | --- | --- | --- | --- | --- | --- | --- | --- | --- | --- |
|  | All individuals  n= 146230 (100%) | No M15  n = 145789 (99.70) | M15  n = 441  (0.30) | No M16  n = 143572 (98.18) | M16  n = 2658 (1.82) | No M17  n = 141496 (96.76) | M17  n = 4734 (3.24) | No M18  n = 145511 (99.51) | M18  n = 719 (0.49) | No M19  n = 143667 (98.25) | M19  n = 2563 (1.75) | |
| **Sex** |  |  |  |  |  |  |  |  |  |  |  | |
| Male | 73185  (50.05) | 73095  (50.14) | 90  (20.41) | 71918 (50.09) | 1267  (47.67) | 70860 (50.08) | 2325  (49.11) | 72988 (50.16) | 197 (27.40) | 71967 (50.09) | 1218  (47.52) | |
| Female | 73045  (49.95) | 72694  (49.86) | 351  (79.59) | 71654 (49.91) | 1391  (52.33) | 70636 (49.92) | 2409  (50.89) | 72523 (49.84) | 522  (72.60) | 71700 (49.91) | 1345 (52.48) | |
| **Education** |  |  |  |  |  |  |  |  |  |  |  | |
| > 11y | 70605  (48.28) | 70479  (48.34) | 126  (28.57) | 69895 (48.65) | 710  (26.71) | 69301 (48.98) | 1310  (27.67) | 70426 (48.40) | 179  (24.90) | 69944 (48.68) | 669  (26.10) | |
| **Year of birth** |  |  |  |  |  |  |  |  |  |  |  | |
| Mean (SD) | 1980.13 (24.44) | 1980.23 (24.41) | 1948.02  (8.19) | 1980.73 (24.22) | 1947.75  (10.52) | 1981.12 (24.15) | 1951.51  (11.35) | 1980.28 (24.40) | 1949.80  (9.43) | 1980.62 (24.33) | 1952.85  (12.49) | |
| Median (IQR) | 1984  (1958-2001) | 1984  (1958-2001) | 1947  (1942-1953) | 1985  (1959-2002) | 1946  (1941-1953) | 1986  (1960-2002) | 1950  (1943-1959) | 1984  (1958-2001) | 1948  (1943-1955) | 1985  (1959-2002) | 1950  (1944-1960) | |
| (Range) | (1932-2018) | (1932-2018) | (1933-1975) | (1932-2018) | (1932-2000) | (1932-2018) | (1932-2015) | (1932-2018) | (1932-2000) | (1932-2018) | (1932-2005) | |
| **Age at end follow-up** |  |  |  |  |  |  |  |  |  |  |  | |
| Mean (SD) | 37.84  (24.01) | 37.77  (24.00) | 61.88  (8.45) | 37.26  (23.81) | 62.62  (10.50) | 36.87  (23.72) | 59.38  (11.50) | 37.71  (23.99) | 60.11  (9.39) | 37.37  (23.91) | 58.48  (12.77) | |
| Median (IQR) | 33.87  (17.04-59.35) | 33.80  (17.04-59.21) | 61.97  (56.61-67.36) | 33.04  (16.80-58.38) | 63.75  (56.99-69.79) | 32.46  (16.54-57.63) | 60.62  (53.11-67.42) | 33.71  (16.6-59.13) | 60.47  (54.95-66.08) | 33.04  (16,80) | 60.14  (51.20-67.76) | |
| (Range) | (0.00-86.96) | (0.00-86.96) | (31.26-82.84) | (0.00-86.96) | (7.76-84.64) | (0.00-86.96) | (11.70-85.16) | (0.00-86.96) | (17.25-82.50) | (0.00-86.96) | (5.10-85.25) | |
| **Age at M onset** |  |  |  |  |  |  |  |  |  |  |  | |
| Mean  (SD) | NA | NA | 61.88  (8.45) | NA | 62.62  (10.50) | NA | 59.38  (11.50) | NA | 60.11  (9.39) | NA | 58.48  (12.77) | |
| Median  (IQR) | NA | NA | 61.97  (56.61-67.36) | NA | 63.75  (56.99-69.79) | NA | 60.62  (53.11-67.42) | NA | 60.47  (54.95-66.08) | NA | 60.14  (51.20-67.76) | |
| (Range) | NA | NA | (31.26-82.84) | NA | (7.76-84.64) | NA | (11.70-85.16) | NA | (17.25-82.50) | NA | (5.10-85.25) | |
| **Comorbidities; %** |  |  |  |  |  |  |  |  |  |  |  | |
| COPD | 1.70 | 1.68 | 6.58 | 1.61 | 6.43 | 1.61 | 4.35 | 1.68 | 5.98 | 1.63 | 5.42 | |
| Alcoholism | 2.94 | 2.94 | 2.95 | 2.91 | 4.06 | 2.89 | 4.16 | 2.93 | 4.03 | 2.91 | 4.53 | |
| Obesity | 1.88 | 1.87 | 4.76 | 1.82 | 5.23 | 1.73 | 6.38 | 1.87 | 4.45 | 1.81 | 5.54 | |
| **Exposure (number affected)** | |  |  |  |  |  |  |  |  |  |  | |
|  | **Twins** | 434  (0.30) | 10  (2.27) | 2214  (1.54) | 452  (17.01) | 3973  (2.81) | 794  (16.77) | 673  (0.46) | 50  (6.95) | 2360  (1.64) | 218  (8.51) | |
|  | **Triplets** | --- | --- | 0  (0.00) | 3  (0.11) | 2  (0.00) | 0  (0.00) | --- | --- | --- | --- | |
| Age at end of follow-up: death, migration, M15-19 diagnosis or end of study period on December 31, 2018, whichever came first; NA, no applicable; SD, standard deviation; IQR, interquartile range.  M15, poly osteoarthritis; M16 osteoarthritis of the hip joint; M17 osteoarthritis of the knee joint; M18 osteoarthritis of the first carpometacarpal joint; M19 other types of osteoarthritis. | | | | | | | | | | | |  |

| **Supplementary Table 2. FULL-SIBLINGS. Characteristics of patients in the full-sibling study sample (unique individuals) for poly osteoarthritis (M15), hip osteoarthritis (M16), knee osteoarthritis (M17), osteoarthritis of first carpometacarpal joint (M18), and other osteoarthritis (M19), respectively.** | | | | | | | | | | | |  |
| --- | --- | --- | --- | --- | --- | --- | --- | --- | --- | --- | --- | --- |
|  | All individuals  n= 5714175 (100%) | No M15  n = 5693345 (99.64) | M15  n = 20830  (0.36) | No M16  n = 5594496 (97.91) | M16  n = 119679 (2.09) | No M17  n = 5496764 (96.20) | M17  n = 217411 (3.80) | No M18  n = 5679990 (99.40) | M18  n = 34185 (0.60) | No M19  n = 5594989 (97.91) | M19  n = 119186 (2.09) | |
| **Sex** |  |  |  |  |  |  |  |  |  |  |  | |
| Male | 2929859  (51.27) | 2925125  (51.38) | 4734  (22.73) | 2873216 (51.36) | 56643 (47.33) | 2822652 (51.35) | 107207 (49.31) | 2921316 (51.43) | 8543 (24.99) | 2873163 (51.35) | 56696 (47.57) | |
| Female | 2784316 (48.73) | 2768220  (48.62) | 16096  (77.27) | 2721280 (48.64) | 63036  (52.67) | 2674112 (48.65) | 110204  (50.69) | 2758674 (48.57) | 25642  (75.01) | 2721826 (48.65) | 62490 (52.43) | |
| **Education** |  |  |  |  |  |  |  |  |  |  |  | |
| > 11y | 2648803  (46.35) | 2642310  (46.41) | 6493 (31.17) | 2615251 (46.75) | 33623  (28.09) | 2586348 (47.05) | 62708  (28.84) | 2639668 (46.47) | 9138 (26.73) | 2616694 (46.77) | 32374  (27.16) | |
| **Year of birth** |  |  |  |  |  |  |  |  |  |  |  | |
| Mean (SD) | 1975.23 (23.22) | 1975.33 (23.20) | 1948.04  (8.76) | 1975.81 (23.06) | 1947.73  (10.35) | 1976.18 (23.06) | 1951.08  (11.19) | 1975.38 (23.20) | 1950.02  (9.55) | 1975.70 (23.17) | 1953.22 (12.30) | |
| Median (IQR) | 1974  (1955-1994) | 1974  (1956-1994) | 1947  (1942-1953) | 1975  (1956-1995) | 1946  (1940-1953) | 1976  (1957-1995) | 1949  (1943-1958) | 1974  (1956-1994) | 1949  (1943-1956) | 1975  (1956-1995) | 1951  (1944-1961) | |
| (Range) | (1932-2018) | (1932-2018) | (1932-2010) | (1932-2018) | (1932-2017) | (1932-2018) | (1932-2015) | (1932-2018) | (1932-2013) | (1932-2018) | (1932-2013) | |
| **Age at end follow-up** |  |  |  |  |  |  |  |  |  |  |  | |
| Mean (SD) | 42.70  (22.77) | 42.63  (22.77) | 61.92  (9.09) | 42.14  (22.63) | 62.61  (10.48) | 41.76  (22.61) | 58.98  (11.44) | 42.57  (22.77) | 60.22  (9.36) | 42.25  (22.74) | 58.24  (12.47) | |
| Median (IQR) | 43.96  (24.21-62.04) | 43.80  (24.13-61.96) | 62.09  (56.41-68.20) | 43.13  (23.80-61.29) | 63.80  (56.79-69.95) | 42.46  (23.46-60.80) | 60.10  (52.16-67.15) | 43.71  (24.04-61.87) | 60.50  (54.66-66.39) | 43.21  (23.80-61.54) | 59.62  (50.94-67.19) | |
| (Range) | (0.00-86.96) | (0.00-86.96) | (0.41-85.38) | (0.00-86.96) | (0.05-86.68) | (0.00-86.96) | (0.54-86.62) | (0.00-86.96) | (1.33-86.28) | (0.00-86.96) | (0.31-86.60) | |
| **Age at M onset** |  |  |  |  |  |  |  |  |  |  |  | |
| Mean  (SD) | NA | NA | 61.92  (9.09) | NA | 62.61  (10.48) | NA | 58.98  (11.44) | NA | 60.22  (9.36) | NA | 58.24  (12.47) | |
| Median  (IQR) | NA | NA | 62.09  (56.41-68.20) | NA | 63.80  (56.79-69.95) | NA | 60.10  (52.16-67.15) | NA | 60.50  (54.66-66.39) | NA | 59.62  (50.94-67.19) | |
| (Range) | NA | NA | (0.41-85.38) | NA | (0.05-86.68) | NA | (0.54-86.62) | NA | (1.33-86.28) | NA | (0.31-86.60) | |
| **Comorbidities; %** |  |  |  |  |  |  |  |  |  |  |  | |
| COPD | 1.77 | 1.76 | 6.44 | 1.68 | 6.26 | 1.67 | 4.55 | 1.74 | 6.86 | 1.70 | 5.38 | |
| Alcoholism | 3.04 | 3.04 | 3.01 | 3.03 | 3.58 | 3.02 | 3.52 | 3.03 | 3.77 | 3.01 | 4.61 | |
| Obesity | 2.85 | 2.84 | 5.46 | 2.77 | 6.81 | 2.64 | 8.22 | 2.84 | 5.77 | 2.77 | 6.73 | |
| **Exposure (number affected)** | |  |  |  |  |  |  |  |  |  |  | |
| **1** | | 40585  (0.71) | 916  (4.40) | 185378  (3.31) | 19726  (16.48) | 306046  (5.57) | 42580  (19.59) | 63573  (1.12) | 2226  (6.51) | 203802  (3.64) | 12930  (10.85) | |
| **2** | | 849  (0.01) | 24  (0.12) | 15593 (0.28) | 2908 (2.43) | 30832  (0.56) | 6906  (3.18) | 1969  (0.03) | 114  (0.33) | 11620  (0.21) | 1073  (0.90) | |
| **3** | | 14  (0.00) | 0  (0.00) | 1870  (0.03) | 546  (0.46) | 4302  (0.08) | 1152  (0.53) | 86  (0.00) | 12  (0.04) | 899  (0.02) | 122  (0.10) | |
| **4** | | --- | --- | 344  (0.01) | 140  (0.12) | 601  (0.01) | 248  (0.11) | 7  (0.00) | 0  (0.00) | 84  (0.00) | 25  (0.05) | |
| **5** | | --- | --- | 92  (0.00) | 30  (0.03) | 162  (0.00) | 48  (0.02) | --- | --- | 15  (0.00) | 6  (0.01) | |
| **6** | | --- | --- | 11  (0.00) | 0  (0.00) | 23  (0.00) | 14  (0.01) | --- | --- | 2  (0.00) | 0  (0.00) | |
| **7** | | --- | --- | --- | --- | 8  (0.00) | 0  (0.00) | --- | --- | --- | --- | |
| Age at end of follow-up: death, migration, M15-19 diagnosis or end of study period on December 31, 2018, whichever came first; NA, no applicable; SD, standard deviation; IQR, interquartile range.  M15, poly osteoarthritis; M16 osteoarthritis of the hip joint; M17 osteoarthritis of the knee joint; M18 osteoarthritis of the first carpometacarpal joint; M19 other types of osteoarthritis. | | | | | | | | | | | |  |

| **Supplementary Table 3. HALF-SIBLINGS. Characteristics of patients in the half-sibling study sample (unique individuals) for poly osteoarthritis (M15), hip osteoarthritis (M16), knee osteoarthritis (M17), osteoarthritis of first carpometacarpal joint (M18), and other osteoarthritis (M19), respectively.** | | | | | | | | | | | |  |
| --- | --- | --- | --- | --- | --- | --- | --- | --- | --- | --- | --- | --- |
|  | All individuals  n= 1374897 (100%) | No M15  n = 1372082 (99.80) | M15  n = 2815  (0.20) | No M16  n = 1360008 (98.92) | M16  n = 14889 (1.08) | No M17  n = 1341774 (97.59) | M17  n = 33123 (2.41) | No M18  n = 1369243 (99.59) | M18  n = 5654 (0.41) | No M19  n = 1352856 (98.40) | M19  n = 22041 (1.60) | |
| **Sex** |  |  |  |  |  |  |  |  |  |  |  | |
| Male | 701997  (51.06) | 701376  (51.12) | 621  (22.06) | 695082 (51.11) | 6915  (46.44) | 685557  (51.09) | 16440  (49.63) | 700685 (51.17) | 1312 (23.20) | 691019 (51.08) | 10978  (49.81) | |
| Female | 672900 (48.94) | 670706  (48.88) | 2194  (77.94) | 664926 (48.89) | 7974  (53.56) | 656217  (48.91) | 16683  (50.37) | 668558 (48.83) | 4342  (76.80) | 661837 (48.92) | 11063  (50.19) | |
| **Education** |  |  |  |  |  |  |  |  |  |  |  | |
| > 11y | 497854  (36.21) | 497160  (36.23) | 694 (24.65) | 494382 (36.35) | 3496  (23.48) | 489966 (36.52) | 7973  (24.07) | 496751 (36.28) | 1106 (19.56) | 493174 (36.45) | 4764  (21.61) | |
| **Year of birth** |  |  |  |  |  |  |  |  |  |  |  | |
| Mean (SD) | 1981.34 (19.75) | 1981.34 (19.73) | 1952.63 (9.49) | 1981.64 (19.61) | 1953.76 (11.68) | 1981.94 (19.54) | 1956.97 (11.41) | 1981.45 (19.71) | 1955.23 (9.97) | 1981.69 (19.66) | 1959.82 (12.38) | |
| Median (IQR) | 1982  (1966-1997) | 1983  (1966-1997) | 1952  (1946-1958) | 1983  (1966-1997) | 1952  (1945-1961) | 1983  (1967-1997) | 1956  (1949-1997) | 1983  (1966-1997) | 1955  (1948-1962) | 1983  (1967-1997) | 1959  (1951-1967) | |
| (Range) | (1932-2018) | (1932-2018) | (1932-1996) | (1932-2018) | (1932-2014) | (1932-2018) | (1932-2007) | (1932-2018) | (1932-2009) | (1932-2018) | (1932-2010) | |
| **Age at end follow-up** |  |  |  |  |  |  |  |  |  |  |  | |
| Mean (SD) | 36.74  (19.45) | 36.69  (19.44) | 57.83  (9.39) | 36.45  (19.32) | 57.28  (11.64) | 36.15  (19.24) | 53.54  (11.66) | 36.64  (19.43) | 55.84  (9.62) | 36.40  (19.36) | 51.97  (12.46) | |
| Median (IQR) | 35.55  (21.80-51.96) | 35.46  (21.80-51.87) | 58.08  (52.39-63.69) | 35.21  (21.63-51.55) | 58.48  (50.46-65.48) | 34.80  (21.38-51.13) | 54.05  (46.33-61.57) | 35.38  (21.71-51.80) | 56.34  (50.38-61.88) | 35.04  (21.54-51.55) | 52.87  (44.16-60.59) | |
| (Range) | (0.00-86.96) | (0.00-86.96) | (15.35-83.43) | (0.00-86.96) | (1.13-85.26) | (0.00-86.96) | (3.78-86.57) | (0.00-86.96) | (1.69-86.28) | (0.00-86.96) | (0.31-85.85) | |
| **Age at M onset** |  |  |  |  |  |  |  |  |  |  |  | |
| Mean  (SD) | NA | NA | 57.83  (9.39) | NA | 57.28  (11.64) | NA | 53.54  (11.66) | NA | 55.84  (9.62) | NA | 51.97  (12.46) | |
| Median  (IQR) | NA | NA | 58.08  (52.39-63.69) | NA | 58.48  (50.46-65.48) | NA | 54.05  (46.33-61.57) | NA | 56.34  (50.38-61.88) | NA | 52.87  (44.16-60.59) | |
| (Range) | NA | NA | (15.35-83.43) | NA | (1.13-85.26) | NA | (3.78-86.57) | NA | (1.69-86.28) | NA | (0.31-85.85) | |
| **Comorbidities; %** |  |  |  |  |  |  |  |  |  |  |  | |
| COPD | 1.25 | 1.24 | 6.75 | 1.19 | 6.39 | 1.18 | 4.27 | 1.23 | 6.93 | 1.19 | 5.01 | |
| Alcoholism | 4.79 | 4.79 | 5.22 | 4.78 | 6.06 | 4.77 | 5.76 | 4.79 | 5.62 | 4.74 | 7.62 | |
| Obesity | 4.10 | 4.09 | 6.75 | 4.04 | 9.34 | 3.94 | 10.52 | 4.08 | 8.24 | 4.01 | 9.19 | |
| **Exposure (number affected)** | |  |  |  |  |  |  |  |  |  |  | |
| **1** | | 5883  (0.43) | 44  (1.56) | 26935  (1.98) | 1299  (8.72) | 54567  (4.07) | 4131  (12.47) | 11450  (0.84) | 230  (4.07) | 41346  (3.06) | 1719  (7.80) | |
| **2** | | 72  (0.01) | 3  (0.11) | 1195  (0.09) | 96 (0.64) | 4031  (0.30) | 457  (1.38) | 267  (0.02) | 4  (0.07) | 1957  (0.14) | 143  (0.65) | |
| **3** | | 1  (0.00) | 0  (0.00) | 112  (0.01) | 15  (0.10) | 444  (0.03) | 63  (0.19) | 14  (0.00) | 0  (0.00) | 121  (0.01) | 13  (0.06) | |
| **4** | | --- | --- | 12  (0.00) | 3  (0.02) | 50  (0.00) | 13  (0.04) | 1  (0.00) | 1  (0.02) | 10  (0.00) | 1  (0.00) | |
| **5** | | --- | --- | 4  (0.00) | 0  (0.00) | 10  (0.00) | 0  (0.00) | --- | --- | 1  (0.00) | 0  (0.00) | |
| **6** | | --- | --- | --- | --- | 0  (0.00) | 1  (0.00) | --- | --- | --- | --- | |
| **7** | | --- | --- | --- | --- | 1  (0.00) | 0  (0.00) | --- | --- | --- | --- | |
| Age at end of follow-up: death, migration, M15-19 diagnosis or end of study period on December 31, 2018, whichever came first; NA, no applicable; SD, standard deviation; IQR, interquartile range.  M15, poly osteoarthritis; M16 osteoarthritis of the hip joint; M17 osteoarthritis of the knee joint; M18 osteoarthritis of the first carpometacarpal joint; M19 other types of osteoarthritis. | | | | | | | | | | | |  |

| **Supplementary Table 4. COUSINS. Characteristics of patients in the cousin study sample (unique individuals) for poly osteoarthritis (M15), hip osteoarthritis (M16), knee osteoarthritis (M17), osteoarthritis of first carpometacarpal joint (M18), and other osteoarthritis (M19), respectively.** | | | | | | | | | | | |  |
| --- | --- | --- | --- | --- | --- | --- | --- | --- | --- | --- | --- | --- |
|  | All individuals  n= 4148991 (100%) | No M15  n = 4146676 (99.94) | M15  n = 2315  (0.06) | No M16  n = 4133283 (99.62) | M16  n = 15708 (0.38) | No M17  n = 4102700 (98.88) | M17  n = 46291 (1.12) | No M18  n = 4143319 (99.86) | M18  n = 5672 (0.14) | No M19  n = 4115704 (99.20) | M19  n = 33287 (0.80) | |
| **Sex** |  |  |  |  |  |  |  |  |  |  |  | |
| Male | 2131583 (51.38) | 2130968  (51.39) | 615  (26.57) | 2123211 (51.37) | 8372  (53.30) | 2106317  (51.34) | 25266  (54.58) | 2130152 (51.41) | 1431  (25.23) | 2113214 (51.35) | 18369  (55.18) | |
| Female | 2017408 (48.62) | 2015708  (48.61) | 1700  (73.43) | 2010072 (48.63) | 7336  (46.70) | 1996383  (48.66) | 21025  (45.42) | 2013167 (48.59) | 4241  (74.77) | 2002490 (48.65) | 14918  (44.82) | |
| **Education** |  |  |  |  |  |  |  |  |  |  |  | |
| > 11y | 2198401  (52.99) | 2197664  (53.00) | 737 (31.84) | 2193018 (53.06) | 5463  (34.78) | 2183020 (53.21) | 15642  (33.79) | 2197023 (53.03) | 1382  (24.37) | 2188704 (53.18) | 9983  (29.99) | |
| **Year of birth** |  |  |  |  |  |  |  |  |  |  |  | |
| Mean (SD) | 1988.13 (16.66) | 1988.15 (16.66) | 1963.76 (7.91) | 1988.21 (16.63) | 1966.63 (8.95) | 1988.37 (16.58) | 1966.96 (8.52) | 1988.17 (16.65) | 1964.52 (7.89) | 1988.29 (16.62) | 1968.83 (9.46) | |
| Median (IQR) | 1988  (1974-2002) | 1988  (1974-2002) | 1963  (1958-1968) | 1988  (1974-2002) | 1965  (1961-1971) | 1989  (1974-2002) | 1966  (1961-1971) | 1988  (1974-2002) | 1963  (1959-1968) | 1988  (1974-2002) | 1967  (1962-1974) | |
| (Range) | (1947-2018) | (1947-2018) | (1949-2010) | (1947-2018) | (1948-2017) | (1947-2018) | (1949-2015) | (1947-2018) | (1949-2017) | (1947-2018) | (1948-2013) | |
| **Age at end follow-up** |  |  |  |  |  |  |  |  |  |  |  | |
| Mean (SD) | 30.16  (16.57) | 30.15  (16.57) | 48.28  (8.73) | 30.08  (16.54) | 45.76  (9.95) | 29.92  (16.48) | 44.31  (9.60) | 30.13  (16.56) | 48.19  (8.75) | 30.00  (16.52) | 43.61  (10.16) | |
| Median (IQR) | 29.87  (16.29-44.13) | 29.87  (16.29-44.04) | 49.48  (43.79-54.27) | 29.80  (16.21-43.96) | 47.32  (40.45-52.61) | 29.71  (16.13-43.71) | 45.55  (38.83-51.12) | 29.87  (16.29-44.04) | 49.49  (43.99-54.08) | 29.71  (16.13-43.87) | 45.06  (37.69-50.95) | |
| (Range) | (0.00-71.55) | (0.00-71.55) | (0.41-68.20) | (0.00-71.55) | (0.05-69.80) | (0.00-71.55) | (0.54-68.60) | (0.00-71.55) | (0.46-68.69) | (0.00-71.55) | (0.08-69.69) | |
| **Age at M onset** |  |  |  |  |  |  |  |  |  |  |  | |
| Mean  (SD) | NA | NA | 48.28  (8.73) | NA | 45.76  (9.95) | NA | 44.31  (9.60) | NA | 48.19  (8.75) | NA | 43.61  (10.16) | |
| Median  (IQR) | NA | NA | 49.48  (43.79-54.27) | NA | 47.32  (40.45-52.61) | NA | 45.55  (38.83-51.12) | NA | 49.49  (43.99-54.08) | NA | 45.06  (37.69-50.95) | |
| (Range) | NA | NA | (0.41-68.20) | NA | (0.05-69.80) | NA | (0.54-68.60) | NA | (0.46-68.69) | NA | (0.08-69.69) | |
| **Comorbidities; %** |  |  |  |  |  |  |  |  |  |  |  | |
| COPD | 0.26 | 0.26 | 2.51 | 0.26 | 1.38 | 0.25 | 1.17 | 0.26 | 2.66 | 0.25 | 1.47 | |
| Alcoholism | 2.71 | 2.71 | 4.28 | 2.70 | 4.80 | 2.69 | 4.07 | 2.71 | 4.64 | 2.69 | 5.69 | |
| Obesity | 2.86 | 2.85 | 7.60 | 2.83 | 8.77 | 2.78 | 9.10 | 2.85 | 8.85 | 2.81 | 7.89 | |
| **Exposure (number affected)** | |  |  |  |  |  |  |  |  |  |  | |
| **1** | | 14880  (0.36) | 46  (1.99) | 93844  (2.27) | 978  (6.23) | 226302  (5.52) | 6663  (14.39) | 36064  (0.87) | 232  (4.09) | 185684  (4.51) | 3635  (10.92) | |
| **2** | | 314  (0.01) | 2  (0.09) | 5942  (0.14) | 86  (0.55) | 31777  (0.77) | 1306  (2.82) | 1390  (0.03) | 6  (0.11) | 17828 (0.43) | 526  (1.58) | |
| **3** | | 15  (0.00) | 0  (0.00) | 542  (0.01) | 2  (0.01) | 5498  (0.13) | 266  (0.57) | 52  (0.00) | 0  (0.00) | 2295 (0.06) | 85  (0.26) | |
| **4** | | --- | --- | 32  (0.00) | 0  (0.00) | 1043  (0.03) | 73  (0.16) | --- | --- | 275  (0.01) | 9  (0.03) | |
| **5** | | --- | --- | 6  (0.00) | 0  (0.00) | 212  (0.01) | 9  (0.02) | --- | --- | 69  (0.00) | 0  (0.00) | |
| **6** | | --- | --- | --- | --- | 45  (0.00) | 1  (0.00) | --- | --- | 19  (0.00) | 7  (0.02) | |
| **7** | | --- | --- | --- | --- | 12  (0.00) | 0  (0.00) | --- | --- | 4  (0.00) | 0  (0.00) | |
| Age at end of follow-up: death, migration, M15-19 diagnosis or end of study period on December 31, 2018, whichever came first; NA, no applicable; SD, standard deviation; IQR, interquartile range.  M15, poly osteoarthritis; M16 osteoarthritis of the hip joint; M17 osteoarthritis of the knee joint; M18 osteoarthritis of the first carpometacarpal joint; M19 other types of osteoarthritis. | | | | | | | | | | | |  |

| **Supplementary Table 5. Fully adjusted familial Hazard ratios (HRs) with 95% confidence intervals (95% CI) among full-siblings, stratified by sex. M15 poly OA, M16 hip OA, M17 knee OA, M18 OA of the first carpometacarpal joint and M19 other OA, respectively.** | | | |
| --- | --- | --- | --- |
|  | | HR^$^ | 95% CI |
| **Poly OA** | Male | **1.96***** | (1.68-2.29) |
|  | Female | **2.39***** | (2.17-2.64) |
|  |  |  |  |
| **Hip OA** | Male | **2.04***** | (1.99-2.10) |
|  | Female | **2.01***** | (1.96-2.06) |
|  |  |  |  |
| **Knee OA** | Male | **1.78***** | (1.75-1.81) |
|  | Female | **1.72***** | (1.69-1.75) |
|  |  |  |  |
| **Thumb OA** | Male | **2.69***** | (2.43-2.97) |
|  | Female | **2.57***** | (2.41-2.74) |
|  |  |  |  |
| **Other OA** | Male | **1.53***** | (1.48-1.58) |
|  | Female | **1.50***** | (1.47-1.56) |
| ^$^Adjusted for education, birth year, COPD (chronic obstructive pulmonary disease), alcoholism, and obesity. All calculations were based on double entry. Significance levels: * p<0.05, ** p<0.01, *** p<0.001 | | | |

| **Supplementary Table 6. Fully adjusted familial Hazard ratios (HRs) with 95% confidence intervals (95% CI) among full-siblings, stratified by birth year (median). M15 poly OA, M16 hip OA, M17 knee OA, M18 OA of the first carpometacarpal joint and M19 other OA, respectively.** | | | |
| --- | --- | --- | --- |
|  | | HR^$^ | 95% CI |
| **Poly OA** | Birth y ≤ 1969 | **2.30***** | (2.10-2.53) |
|  | Birth y > 1969 | **4.32*** | (1.08-17.34) |
|  |  |  |  |
| **Hip OA** | Birth y ≤ 1969 | **2.03***** | (1.99-2.08) |
|  | Birth y > 1969 | **3.08***** | (2.97-4.63) |
|  |  |  |  |
| **Knee OA** | Birth y ≤ 1969 | **1.73***** | (1.71-1.76) |
|  | Birth y > 1969 | **2.39***** | (2.18-2.61) |
|  |  |  |  |
| **Thumb OA** | Birth y ≤ 1969 | **2.57***** | (2.42-2.73) |
|  | Birth y > 1969 | **3.46***** | (1.99-6.02) |
|  |  |  |  |
| **Other OA** | Birth y ≤ 1969 | **1.49***** | (1.45-1.53) |
|  | Birth y > 1969 | **1.93***** | (1.71-2.18) |
| ^$^Adjusted for sex, education, birth year, COPD (chronic obstructive pulmonary disease), alcoholism, and obesity. All calculations were based on double entry. Significance levels: * p<0.05, ** p<0.01, *** p<0.001 | | | |

| **Supplementary Table 7. Additional analysis with adjustment also for cancer and opioid use (Model 4). Familial Hazard ratios (HRs) with 95% confidence intervals (95% CI) among full-siblings for (ICD-10= M15, M16, M17, M18, M19 ) for individuals with relative history of M15, M16, M17, M18, M19 compared with those with non-affected relatives ( n = 9989446).** | | | | | | | | |
| --- | --- | --- | --- | --- | --- | --- | --- | --- |
| Variable | Person-years,  No. | Cases, No./Persons at risk, No. | Incidence rate, cases/1000 person-years | Incidence rate ratio (95%CI) | HR (95% CI) | | | |
|  |  |  |  |  | **Model 1** | **Model 2** | **Model 3** | **Model 4** |
| **M15** |  |  |  |  |  |  |  |  |
| Non-affected | 193103424 | 42325/9946157 | 0.22  (0.21-0.22) | 1 [Reference] | 1 [Reference] | 1 [Reference] | 1 [Reference] | 1 [Reference] |
| Affected | 885178 | 964/43289 | 1.09  (1.02-1.16) | **4.97*****  (4.66-5.30) | **4.73*****  (4.32-5.18) | **2.30*****  (2.30-2.53) | **2.29*****  (2.09-2.51) | **2.20*****  (2.01-2.41) |
| **M16** |  |  |  |  |  |  |  |  |
| Non-affected | 187446050 | 224076/9737480 | 1.20  (1.19-1.20) | 1 [Reference] | 1 [Reference] | 1 [Reference] | 1 [Reference] | 1 [Reference] |
| Affected | 4949965 | 27890/251966 | 5.63  (5.57-5.70) | **4.71*****  (4.66-4.77) | **4.50*****  (4.40-4.59) | **2.03*****  (1.99-2.08) | **2.04*****  (1.98-2.07) | **1.94*****  (1.90-1.98) |
| **M17** |  |  |  |  |  |  |  |  |
| Non-affected | 181979682 | 384024/9544258 | 2.11  (2.10-2.12) | 1 [Reference] | 1 [Reference] | 1 [Reference] | 1 [Reference] | 1 [Reference] |
| Affected | 8718710 | 61164/445188 | 7.02  (6.96-7.07) | **3.32*****  (3.30-3.35) | **3.20*****  (3.16-3.25) | **1.78*****  (1.75-1.80) | **1.75*****  (1.73-1.77) | **1.67*****  (1.65-1.70) |
| **M18** |  |  |  |  |  |  |  |  |
| Non-affected | 192350100 | 67797/9919159 | 0.35  (0.35-0.36) | 1 [Reference] | 1 [Reference] | 1 [Reference] | 1 [Reference] | 1 [Reference] |
| Affected | 1435871 | 2490/70287 | 1.73  (1.67-1.80) | **4.92*****  (4.73-5.12) | **4.70*****  (4.42-4.99) | **2.62*****  (2.46-2.78) | **2.60*****  (2.45-2.76) | **2.48*****  (2.33-2.63) |
| **M19** |  |  |  |  |  |  |  |  |
| Non-affected | 187650766 | 230162/9743712 | 1.23  (1.22-1.23) | 1 [Reference] | 1 [Reference] | 1 [Reference] | 1 [Reference] | 1 [Reference] |
| Affected | 5007881 | 15572/245734 | 3.11  (3.06-3.16) | **2.54*****  (2.49-2.78) | **2.41*****  (2.35-2.47) | **1.53*****  (1.50-1.57) | **1.52*****  (1.48-1.56) | **1.43*****  (1.39-1.46) |
| HR, Hazard ratio. Model 1 crude. Model 2 adjusted for sex, education and birth year. Model 3 adjusted additionally for COPD, alcoholism and obesity. Model 4 adjusted additionally for cancer and opioid use (ATC code=N02A). All calculations were based on double entry. M15, poly osteoarthritis; M16 osteoarthritis of the hip joint; M17 osteoarthritis of the knee joint; M18 osteoarthritis of the first carpometacarpal joint ; M19 other types of osteoarthritis. Significance levels: * p<0.05, ** p<0.01, *** p<0.001 | | | | | | | | |

| **Supplementary Table 8. Exclusion of patients diagnosed with cancer or chronic obstructive pulmonary disease (COPD) with adjustment for opioid use. Familial Hazard ratios (HRs) with 95% confidence intervals (95% CI) among full-siblings for (ICD-10 = M15, M16, M17, M18, M19 ) for individuals with relative history of M15, M16, M17, M18, M19 compared with those with non-affected relatives (n = 84961109.** | | | | | | | |
| --- | --- | --- | --- | --- | --- | --- | --- |
| Variable | Person-years,  No. | Cases, No./Persons at risk, No. | Incidence rate, cases/1000 person-years | Incidence rate ratio (95%CI) | HR(95% CI) | | |
|  |  |  |  |  | **Model 1** | **Model 2** | **Model 3** |
| **M15** |  |  |  |  |  |  |  |
| Non-affected | 164119840 | 27553/8467997 | 0.17  (0.17-0.17) | 1 [Reference] | 1 [Reference] | 1 [Reference] | 1 [Reference] |
| Affected | 591351 | 560/28113 | 0.95  (0.87-1.03) | **5.64*****  (5.19-6.13) | **5.40*****  (4.78-6.10) | **2.17*****  (1.92-2.45) | **2.07*****  (1.83-2.34) |
| **M16** |  |  |  |  |  |  |  |
| Non-affected | 160383532 | 146472/8332586 | 0.91  (0.91-0.92) | 1 [Reference] | 1 [Reference] | 1 [Reference] | 1 [Reference] |
| Affected | 3319614 | 17052/163524 | 5.14  (5.06-5.21) | **5.63*****  (5.54-5.72) | **5.39*****  (5.26-5.53) | **2.08*****  (2.02-2.13) | **1.96*****  (1.91-2.02) |
| **M17** |  |  |  |  |  |  |  |
| Non-affected | 156193129 | 269166/8186056 | 1.72  (1.72-1.73) | 1 [Reference] | 1 [Reference] | 1 [Reference] | 1 [Reference] |
| Affected | 6245952 | 40888/310054 | 6.55  (6.48-6.61) | **3.80*****  (3.76-3.84) | **3.67*****  (3.61-3.73) | **1.79*****  (1.76-1.82) | **1.68*****  (1.65-1.71) |
| **M18** |  |  |  |  |  |  |  |
| Non-affected | 163584491 | 45519/8448915 | 0.28  (0.28-0.28) | 1 [Reference] | 1 [Reference] | 1 [Reference] | 1 [Reference] |
| Affected | 989346 | 1676/47195 | 1.69  (1.61-1.78) | **6.09*****  (5.80-6.39) | **5.82*****  (5.41-6.26) | **2.80*****  (2.60-3.01) | **2.64*****  (2.45-2.84) |
| **M19** |  |  |  |  |  |  |  |
| Non-affected | 160139807 | 163265/8322453 | 1.02  (1.01-1.02) | 1 [Reference] | 1 [Reference] | 1 [Reference] | 1 [Reference] |
| Affected | 3625322 | 10392/173657 | 2.87  (2.87-2.92) | **2.81*****  (2.76-2.87) | **2.66*****  (2.59-2.74) | **1.54*****  (1.49-1.58) | **1.43*****  (1.38-1.47) |
| HR, Hazard ratio. Model 1 crude. Model 2 adjusted for sex, education and birth year. Model 3 adjusted additionally for alcoholism, obesity, and opioid use (ATC code=N02A). All calculations were based on double entry. M15, poly osteoarthritis; M16 osteoarthritis of the hip joint; M17 osteoarthritis of the knee joint; M18 osteoarthritis of the first carpometacarpal joint; M19 other types of osteoarthritis. Significance levels: * p<0.05, ** p<0.01, *** p<0.001 | | | | | | | |

| **Supplementary Table 9. Adjustment for occupation (Model 4). Familial Hazard ratios (HRs) with 95% confidence intervals (95% CI) among full-siblings for (ICD-10= M15, M16, M17, M18, M19 ) for individuals with relative history of M15, M16, M17, M18, M19 compared with those with non-affected relatives (n = 9989446).** | | | | | | | | |
| --- | --- | --- | --- | --- | --- | --- | --- | --- |
| Variable | Person-years,  No. | Cases, No./Persons at risk, No. | Incidence rate, cases/1000 person-years | Incidence rate ratio (95%CI) | HR(95% CI) | | | |
|  |  |  |  |  | **Model 1** | **Model 2** | **Model 3** | **Model 4** |
| **M15** |  |  |  |  |  |  |  |  |
| Non-affected | 193103424 | 42325/9946157 | 0.22  (0.21-0.22) | 1 [Reference] | 1 [Reference] | 1 [Reference] | 1 [Reference] | 1 [Reference] |
| Affected | 885178 | 964/43289 | 1.09  (1.02-1.16) | **4.97*****  (4.66-5.30) | **4.73*****  (4.32-5.18) | **2.30*****  (2.30-2.53) | **2.29*****  (2.09-2.51) | **2.29*****  (2.09-2.51) |
| **M16** |  |  |  |  |  |  |  |  |
| Non-affected | 187446050 | 224076/9737480 | 1.20  (1.19-1.20) | 1 [Reference] | 1 [Reference] | 1 [Reference] | 1 [Reference] | 1 [Reference] |
| Affected | 4949965 | 27890/251966 | 5.63  (5.57-5.70) | **4.71*****  (4.66-4.77) | **4.50*****  (4.40-4.59) | **2.03*****  (1.99-2.08) | **2.04*****  (1.98-2.07) | **2.02*****  (1.98-2.06) |
| **M17** |  |  |  |  |  |  |  |  |
| Non-affected | 181979682 | 384024/9544258 | 2.11  (2.10-2.12) | 1 [Reference] | 1 [Reference] | 1 [Reference] | 1 [Reference] | 1 [Reference] |
| Affected | 8718710 | 61164/445188 | 7.02  (6.96-7.07) | **3.32*****  (3.30-3.35) | **3.20*****  (3.16-3.25) | **1.78*****  (1.75-1.80) | **1.75*****  (1.73-1.77) | **1.75*****  (1.72-1.77) |
| **M18** |  |  |  |  |  |  |  |  |
| Non-affected | 192350100 | 67797/9919159 | 0.35  (0.35-0.36) | 1 [Reference] | 1 [Reference] | 1 [Reference] | 1 [Reference] | 1 [Reference] |
| Affected | 1435871 | 2490/70287 | 1.73  (1.67-1.80) | **4.92*****  (4.73-5.12) | **4.70*****  (4.42-4.99) | **2.62*****  (2.46-2.78) | **2.60*****  (2.45-2.76) | **2.60*****  (2.45-2.76) |
| **M19** |  |  |  |  |  |  |  |  |
| Non-affected | 187650766 | 230162/9743712 | 1.23  (1.22-1.23) | 1 [Reference] | 1 [Reference] | 1 [Reference] | 1 [Reference] | 1 [Reference] |
| Affected | 5007881 | 15572/245734 | 3.11  (3.06-3.16) | **2.54*****  (2.49-2.78) | **2.41*****  (2.35-2.47) | **1.53*****  (1.50-1.57) | **1.52*****  (1.48-1.56) | **1.52*****  (1.48-1.56) |
| HR, Hazard ratio. Model 1 crude. Model 2 adjusted for sex, education and birth year. Model 3 adjusted additionally for COPD, alcoholism and obesity. Model 4 adjusted additionally for occupation. All calculations were based on double entry. M15, poly osteoarthritis; M16 osteoarthritis of the hip joint; M17 osteoarthritis of the knee joint; M18 osteoarthritis of the first carpometacarpal joint; M19 other types of osteoarthritis. Significance levels: * p<0.05, ** p<0.01, *** p<0.001. | | | | | | | | |

| **Supplementary Table 10. Inclusion only of one sibling pair from each family. In multiplex families only the two oldest siblings were included (double entry). Familial Hazard ratios (HRs) with 95% confidence intervals (95% CI) full-siblings for (ICD-10= M15, M16, M17, M18, M19 ) for individuals with relative history of M15, M16, M17, M18, M19 compared with those with non-affected relatives (n = 4602704).** | | | |
| --- | --- | --- | --- |
| Variable | HR(95% CI) | | |
|  | **Model 1** | **Model 2** | **Model 3** |
| **M15** |  |  |  |
| Affected | **5.88*****  (5.09-6.80) | **2.32*****  (2.01-2.68) | **2.31*****  (2.00-2.67) |
| **M16** |  |  |  |
| Affected | **5.64*****  (5.48-5.80) | **2.05*****  (1.99-2.11) | **2.04*****  (1.98-2.10) |
| **M17** |  |  |  |
| Affected | **3.80*****  (3.73-3.88) | **1.79*****  (1.76-1.83) | **1.77*****  (1.73-1.80) |
| **M18** |  |  |  |
| Affected | **5.89*****  (5.38-6.45) | **2.72*****  (2.49-2.98) | **2.70*****  (2.46-2.96) |
| **M19** |  |  |  |
| Affected | **2.79*****  (2.68-2.89) | **1.58*****  (1.52-1.64) | **1.56*****  (1.51-1.63) |
| HR, Hazard ratio. Model 1 crude. Model 2 adjusted for sex, education and birth year. Model 3 adjusted additionally for COPD, alcoholism and obesity. All calculations were based on double entry. M15, poly osteoarthritis; M16 osteoarthritis of the hip joint; M17 osteoarthritis of the knee joint; M18 osteoarthritis of the first carpometacarpal joint; M19 other types of osteoarthritis. Significance levels: * p<0.05, ** p<0.01, *** p<0.001 | | | |

| **Supplementary Table 11. Inclusion only of one sibling pair from each family. In multiplex families only the two oldest siblings were included (single entry). Familial Hazard ratios (HRs) with 95% confidence intervals (95% CI) among full-siblings for (ICD-10= M15, M16, M17, M18, M19 ) for individuals with relative history of M15, M16, M17, M18, M19 compared with those with non-affected relatives (n = 2301352).** | | | |
| --- | --- | --- | --- |
| Variable | HR(95% CI) | | |
|  | **Model 1** | **Model 2** | **Model 3** |
| **M15** |  |  |  |
| Affected | **6.02*****  (5.12-6.96) | **2.25*****  (1.94-2.60) | **2.23*****  (1.93-2.58) |
| **M16** |  |  |  |
| Affected | **5.84*****  (5.68-6.01) | **2.01*****  (1.95-2.07) | **2.00*****  (1.94-2.06) |
| **M17** |  |  |  |
| Affected | **3.90*****  (3.82-3.98) | **1.76*****  (1.72-1.79) | **1.74*****  (1.70-1.77) |
| **M18** |  |  |  |
| Affected | **6.03*****  (5.51-6.60) | **2.66*****  (2.42-2.91) | **2.63*****  (2.40-2.88) |
| **M19** |  |  |  |
| Affected | **2.84*****  (2.74-2.96) | **1.56*****  (1.50-1.62) | **1.55*****  (1.49-1.61) |
| HR, Hazard ratio. Model 1 crude. Model 2 adjusted for sex, education and birth year. Model 3 adjusted additionally for COPD, alcoholism and obesity.  All calculations were based on single entry. M15, poly osteoarthritis; M16 osteoarthritis of the hip joint; M17 osteoarthritis of the knee joint; M18 osteoarthritis of the first carpometacarpal joint; M19 other types of osteoarthritis. Significance levels: * p<0.05, ** p<0.01, *** p<0.001 | | | |

| **Supplementary Table 12. Twin model poly OA. Familial Hazard ratios (HRs) with 95% confidence intervals (95% CI) among twins for poly OA (ICD-10 = M15) for individuals with relative history of poly OA, M15 compared with those with non-affected relatives. (n= 148758).** | | | | | | |
| --- | --- | --- | --- | --- | --- | --- |
|  |  | HR(95% CI) | | | | |
| **Variables** | Ref. | **Model 1** | **Model 2** | **Model 3** | **Model 4** | **Model 5** |
| **Affected** | No | **6.27*****  (2.58-15.27) | 2.41  (0.98-5.91) | **5.85*****  (2.40-14.25) | 2.21  (0.90-5.41) | 2.18  (0.89-5.36) |
| **Year of birth** |  | **0.93*****  (0.92-0.93) | **0.93*****  (0.93-0.93) |  | **0.93*****  (0.92-0.93) | **0.93*****  (0.92-0.93) |
| **Sex** | Male | **3.95*****  (3.12-4.99) |  | **3.93*****  (3.11-4.96) | **3.76*****  (2.98-4.75) | **3.73*****  (2.95-4.72) |
| **Education** |  |  |  |  |  |  |
| 10 – 11 y | <= 9 y or missing | **0.55*****  (0.43-0.69) |  |  | **1.28***  (1.01-1.62) | **1.28***  (1.01-1.62) |
| > 11 y |  | **0.34*****  (0.26-0.44) |  |  | 1.31  (1.00-1.73) | 1.34  (1.01-1.76) |
| **Comorbidities** |  |  |  |  |  |  |
| COPD | No | **3.58*****  (2.44-5.55) |  |  |  | 1.13  (0.76-1.68) |
| Alcoholism | No | 0.80  (0.46-1.40) |  |  |  | 0.92  (0.52-1.63) |
| Obesity | No | **2.28*****  (1.47-3.53) |  |  |  | **1.92****  (1.23-2.99) |
| HR, Hazard ratio.  Model 1 crude.  Model 2 adjusted for year of birth.  Model 3 adjusted for sex.  Model 4 adjusted for sex, education and birth year.  Model 5 adjusted additionally for COPD, alcoholism and obesity.  All calculations were based on double entry. Significance levels: * p<0.05, ** p<0.01, *** p<0.001 | | | | | | |

| **Supplementary Table 13. Twin model hip OA. Familial Hazard ratios (HRs) with 95% confidence intervals (95% CI) among twins for hip OA (ICD-10 = M16) for individuals with relative history of hip OA. M16 compared with those with non-affected relatives. (n=** **148758)** | | | | | | |
| --- | --- | --- | --- | --- | --- | --- |
|  |  | HR(95% CI) | | | | |
| **Variables** | Ref. | **Model 1** | **Model 2** | **Model 3** | **Model 4** | **Model 5** |
| **Affected** | No | **9.89*****  (8.55-11.44) | **3.45*****  (2.97-4.01) | **9.88*****  (8.54-11.43) | **3.44*****  (2.96-4.00) | **3.44*****  (2.96-4.01) |
| **Year of birth** |  | **0.93*****  (0.92-0.93) | **0.93*****  (0.93-0.93) |  | **0.93*****  (0.92-0.93) | **0.93*****  (0.92-0.93) |
| **Sex** | Male | **1.10***  (1.02-1.19) |  | **1.09*****  (1.01-1.18) | 1.05  (0.97-1.14) | 1.05  (0.97-1.13) |
| **Education** |  |  |  |  |  |  |
| 10 – 11 y | <= 9 y or missing | **0.46*****  (0.42-0.51) |  |  | **1.13****  (1.03-1.25) | **1.13****  (1.03-1.25) |
| > 11 y |  | **0.27*****  (0.25-0.31) |  |  | **1.14***  (1.02-1.27) | **1.14***  (1.02-1.27) |
| **Comorbidities** |  |  |  |  |  |  |
| COPD | No | **3.45*****  (2.95-4.05) |  |  |  | 1.06  (0.90-1.25) |
| Alcoholism | No | 1.11  (0.92-1.35) |  |  |  | 1.01  (0.83-1.23) |
| Obesity | No | **2.60*****  (2.18-3.09) |  |  |  | **2.36*****  (1.98-2.81) |
| HR, Hazard ratio.  Model 1 crude.  Model 2 adjusted for year of birth.  Model 3 adjusted for sex.  Model 4 adjusted for sex, education and birth year.  Model 5 adjusted additionally for COPD, alcoholism and obesity.  All calculations were based on double entry. Significance levels: * p<0.05, ** p<0.01, *** p<0.001 | | | | | | |

| **Supplementary Table 14. Twin model knee OA. Familial Hazard ratios (HRs) with 95% confidence intervals (95% CI) among twins for knee OA (ICD-10 = M17) for individuals with relative history of knee OA, M17 compared with those with non-affected relatives. (n=** **148758)** | | | | | | |
| --- | --- | --- | --- | --- | --- | --- |
|  |  | HR(95% CI) | | | | |
| **Variables** | Ref. | **Model 1** | **Model 2** | **Model 3** | **Model 4** | **Model 5** |
| **Affected** | No | **9.89*****  (8.55-11.44) | **3.45*****  (2.97-4.01) | **9.88*****  (8.54-11.43) | **3.44*****  (2.96-4.00) | **3.44*****  (2.96-4.01) |
| **Year of birth** |  | **0.93*****  (0.92-0.93) | **0.93*****  (0.93-0.93) |  | **0.93*****  (0.92-0.93) | **0.93*****  (0.92-0.93) |
| **Sex** | Male | **1.10***  (1.02-1.19) |  | **1.09*****  (1.01-1.18) | 1.05  (0.97-1.14) | 1.05  (0.97-1.13) |
| **Education** |  |  |  |  |  |  |
| 10 – 11 y | <= 9 y or missing | **0.46*****  (0.42-0.51) |  |  | **1.13****  (1.03-1.25) | **1.13****  (1.03-1.25) |
| > 11 y |  | **0.27*****  (0.25-0.31) |  |  | **1.14***  (1.02-1.27) | **1.14***  (1.02-1.27) |
| **Comorbidities** |  |  |  |  |  |  |
| COPD | No | **3.45*****  (2.95-4.05) |  |  |  | 1.06  (0.90-1.25) |
| Alcoholism | No | 1.11  (0.92-1.35) |  |  |  | 1.01  (0.83-1.23) |
| Obesity | No | **2.60*****  (2.18-3.09) |  |  |  | **2.36*****  (1.98-2.81) |
| HR, Hazard ratio.  Model 1 crude.  Model 2 adjusted for year of birth.  Model 3 adjusted for sex.  Model 4 adjusted for sex, education and birth year.  Model 5 adjusted additionally for COPD, alcoholism and obesity.  All calculations were based on double entry. Significance levels: * p<0.05, ** p<0.01, *** p<0.001 | | | | | | |

| **Supplementary Table 15. Twin model thumb OA. Familial Hazard ratios (HRs) with 95% confidence intervals (95% CI) among twins for OA of the first carpometacarpal joint (ICD-10 = M18) for individuals with relative history of OA of the first carpometacarpal joint, M18 compared with those with non-affected relatives. (n=** **148758)** | | | | | | |
| --- | --- | --- | --- | --- | --- | --- |
|  |  | HR(95% CI) | | | | |
| **Variables** | Ref. | **Model 1** | **Model 2** | **Model 3** | **Model 4** | **Model 5** |
| **Affected** | No | **12.68*****  (8.42-19.08) | **5.64*****  (3.73-8.52) | **12.14*****  (8.08-18.23) | **5.35*****  (3.55-8.07) | **5.33*****  (3.54-8.04) |
| **Year of birth** |  | **0.93*****  (0.92-0.94) | **0.94*****  (0.93-0.94) |  | **0.94*****  (0.93-0.94) | **0.94*****  (0.93-0.94) |
| **Sex** | Male | **2.63*****  (2.22-3.10) |  | **2.59*****  (2.20-3.06) | **2.51*****  (2.13-2.96) | **2.51*****  (2.13-2.96) |
| **Education** |  |  |  |  |  |  |
| 10 – 11 y | <= 9 y or missing | **0.73*****  (0.60-0.88) |  |  | **1.59****  (1.31-1.92) | **1.60*****  (1.31-1.93) |
| > 11 y |  | **0.34*****  (0.27-0.42) |  |  | 1.18  (0.93-1.49) | 1.20  (0.95-1.52) |
| **Comorbidities** |  |  |  |  |  |  |
| COPD | No | **3.29*****  (2.39-4.53) |  |  |  | 1.10  (0.80-1.53) |
| Alcoholism | No | 1.15  (0.79-1.68) |  |  |  | 1.22  (0.83-1.74) |
| Obesity | No | **2.13*****  (1.50-3.03) |  |  |  | **1.73****  (1.22-2.45) |
| HR, Hazard ratio.  Model 1 crude.  Model 2 adjusted for year of birth.  Model 3 adjusted for sex.  Model 4 adjusted for sex, education and birth year.  Model 5 adjusted additionally for COPD, alcoholism and obesity.  All calculations were based on double entry. Significance levels: * p<0.05, ** p<0.01, *** p<0.001 | | | | | | |

| **Supplementary Table 16. Twin model other OA. Familial Hazard ratios (HRs) with 95% confidence intervals (95% CI) among twins for other OA (ICD-10 = M19) for individuals with relative history of other OA, M19 compared with those with non-affected relatives. (n=** **148758)** | | | | | | |
| --- | --- | --- | --- | --- | --- | --- |
|  |  | HR(95% CI) | | | | |
| **Variables** | Ref. | **Model 1** | **Model 2** | **Model 3** | **Model 4** | **Model 5** |
| **Affected** | No | **4.22*****  (3.47-5.14) | **2.22*****  (1.82-2.71) | **4.22*****  (3.47-5.13) | **2.22*****  (1.19-2.70) | **2.21*****  (1.81-2.70) |
| **Year of birth** |  | **0.95*****  (0.95-0.95) | **0.95*****  (0.95-0.95) |  | **0.95*****  (0.95-0.95) | **0.95*****  (0.95-0.95) |
| **Sex** | Male | **1.10***  (1.02-1.19) |  | **1.10***  (1.01-1.19) | 1.26  (0.99-1.16) | 1.06  (0.98-1.15) |
| **Education** |  |  |  |  |  |  |
| 10 – 11 y | <= 9 y or missing | **0.63*****  (0.57-0.70) |  |  | **1.26*****  (1.14-1.40) | **1.27*****  (1.15-1.41) |
| > 11 y |  | **0.33*****  (0.30-0.37) |  |  | 0.99  (0.88-1.11) | 1.10  (0.90-1.14) |
| **Comorbidities** |  |  |  |  |  |  |
| COPD | No | **2.86*****  (2.40-3.40) |  |  |  | 1.06  (0.89-1.27) |
| Alcoholism | No | **1.23***  (1.02-1.48) |  |  |  | 1.10  (0.91-1.33) |
| Obesity | No | **2.72*****  (2.29-3.22) |  |  |  | **2.38*****  (2.00-2.83) |
| HR, Hazard ratio.  Model 1 crude.  Model 2 adjusted for year of birth.  Model 3 adjusted for sex.  Model 4 adjusted for sex, education and birth year.  Model 5 adjusted additionally for COPD, alcoholism and obesity.  All calculations were based on double entry. Significance levels: * p<0.05, ** p<0.01, *** p<0.001 | | | | | | |

| **Supplementary Table 17. Aggregated analysis of twins, full-siblings, half-siblings, and cousins in the same model. Fully adjusted Odds ratio, Hazard ratio and Competing-event analysis according to Fine and Gray in family members by degree of relationship (siblings, half-siblings, and cousins in one dataset (n=38 823 132, double entry) for all types of OA (ICD-10= M15, M16, M17, M18, or M19). Individuals with relative history of OA (M15, M16, M17, M18, or M19) are compared with those with non-affected relatives.** | | | |
| --- | --- | --- | --- |
|  | ^‡^OR (95% CI) | ^&^HR(95% CI) | ^£^HR(95% CI) |
| **M15** |  |  |  |
| Twins affected | 2.00  (0.81-4.95) | 2.02  (0.84-4.84) | 1.99  (0.83-4.78) |
| Full-Siblings affected | **2.17*****  (1.97-2.38) | **2.15*****  (1.96-2.36) | **2.15*****  (1.96-2.36) |
| Half-Siblings affected | 1.47  (0.99-2.17) | 1.46  (0.99-2.15) | 1.46  (0.99-2.16) |
| Cousins affected | **2.23*****  (1.66-3.00) | **2.18*****  (162-2.93) | **2.20*****  (1.64-2.96) |
| **M16** |  |  |  |
| Twins affected | **3.27*****  (2.82-3.78) | **3.03*****  (2.67-3.45) | **3.02*****  (2.65-3.43) |
| Full-Siblings affected | **2.00*****  (1.96-2.05) | **1.95*****  (1.91-1.99) | **1.94*****  (1.90-1.98) |
| Half-Siblings affected | **1.55*****  (1.45-1.66) | **1.54*****  (1.44-1.64) | **1.54*****  (1.44-1.64) |
| Cousins affected | **1.29*****  (1.21-1.37) | **1.26*****  (1.18-1.34) | **1.27*****  (1.19-1.35) |
| **M17** |  |  |  |
| Twins affected | **2.01*****  (1.80-2.24) | **1.90*****  (1.73-2.09) | **1.91*****  (1.73-2.10) |
| Full-Siblings affected | **1.60*****  (1.58-1.63) | **1.57*****  (1.55-1.59) | **1.57*****  (1.54-1.59) |
| Half-Siblings affected | **1.44*****  (1.38-1.50) | **1.42*****  (1.37-1.47) | **1.42*****  (1.37-1.47) |
| Cousins affected | **1.53*****  (1.50-1.57) | **1.48*****  (1.45-1.51) | **1.50*****  (1.46-1.53) |
| **M18** |  |  |  |
| Twins affected | **4.63*****  (3.07-6.99) | **4.42*****  (2.99-6.54) | **4.55*****  (3.09-6.70) |
| Full-Siblings affected | **2.36*****  (2.22-2.51) | **2.32*****  (2.19-2.47) | **2.33*****  (2.19-2.48) |
| Half-Siblings affected | **2.20*****  (1.85-2.61) | **2.16*****  (1.82-2.55) | **2.17*****  (1.84-2.56) |
| Cousins affected | **1.97*****  (1.74-2.24) | **1.90*****  (1.68-2.16) | **1.93*****  (1.70-2.19) |
| **M19** |  |  |  |
| Twins affected | **1.89*****  (1.55-2.30) | **1.86*****  (1.55-2.24) | **1.90*****  (1.58-2.27) |
| Full-Siblings affected | **1.32*****  (1.29-1.36) | **1.34*****  (1.31-1.37) | **1.34*****  (1.30-1.37) |
| Half-Siblings affected | **1.45*****  (1.37-1.54) | **1.44*****  (1.36-1.52) | **1.44*****  (1.36-1.53) |
| Cousins affected | **1.51*****  (1.47-1.56) | **1.44*****  (1.36-1.52) | **1.46*****  (1.42-1.51) |
| ^‡^Logistic regression model. ^&^Cox regression model. ^£^Competing-event analysis according to Fine and Gray.  Full model adjusted for sex, education and birth year, COPD, alcoholism and obesity. All calculations were based on double entry. M15, poly osteoarthritis; M16 osteoarthritis of the hip joint; M17 osteoarthritis of the knee joint; M18 osteoarthritis of the first carpometacarpal joint; M19 other types of osteoarthritis. Significance levels: * p<0.05, ** p<0.01, *** p<0.001. | | | |

| **Supplementary Table 18. PARENT-OFFSPRING. Familial Hazard ratios (HRs) with 95% confidence intervals (95% CI) among parent-offspring for (ICD-10= M15, M16, M17, M18, or M19) for individuals with relative history of other osteoarthritis compared with those with non-affected relatives (n=5133048). All families with one or both parents deceased before 1997 were excluded.** | | | | | | | |
| --- | --- | --- | --- | --- | --- | --- | --- |
| Variable | Person-years,  No. | Cases, No./Persons at risk, No. | Incidence rate, cases/1000 person-years | Incidence rate ratio (95%CI) | HR(95% CI) | | |
|  |  |  |  |  | **Model 1** | **Model 2** | **Model 3** |
| **M15** |  |  |  |  |  |  |  |
| Non-affected | 92898775 | 7822/5068626 | 0.08  (0.08-0.09) | 1 [Reference] | 1 [Reference] | 1 [Reference] | 1 [Reference] |
| Affected | 1377956 | 229/64422 | 0.17  (0.15-0.19) | **1.97*****  (1.73-2.25) | **1.85*****  (1.62-2.12) | **2.09*****  (1.83-2.40) | **2.08*****  (1.82-2.39) |
| **M16** |  |  |  |  |  |  |  |
| Non-affected | 85339075 | 34528/4721441 | 0.41  (0.40-0.41) | 1 [Reference] | 1 [Reference] | 1 [Reference] | 1 [Reference] |
| Affected | 8699487 | 9558/411607 | 1.10  (1.08-1.12) | **2.72*****  (2.66-2.78) | **2.52*****  (2.46-2.58) | **1.95*****  (1.91-2.00) | **1.95*****  (1.90-1.99) |
| **M17** |  |  |  |  |  |  |  |
| Non-affected | 79679762 | 76635/4464637 | 0.96  (0.96-0.97) | 1 [Reference] | 1 [Reference] | 1 [Reference] | 1 [Reference] |
| Affected | 13876835 | 25980/668411 | 1.87  (1.85-2.16) | **1.95*****  (1.92-1.97) | **1.84*****  (1.81-1.86) | **1.80*****  (1.77-1.83) | **1.77*****  (1.75-1.80) |
| **M18** |  |  |  |  |  |  |  |
| Non-affected | 92001925 | 14421/5028469 | 0.16  (0.15-0.16) | 1 [Reference] | 1 [Reference] | 1 [Reference] | 1 [Reference] |
| Affected | 2229323 | 681/104579 | 0.31  (0.28-0.33) | **1.95*****  (1.81-2.11) | **1.82*****  (1.68-1.97) | **2.33*****  (2.15-2.52) | **2.31*****  (2.13-2.50) |
| **M19** |  |  |  |  |  |  |  |
| Non-affected | 86813079 | 55861/4788140 | 0.64  (0.64-0.65) | 1 [Reference] | 1 [Reference] | 1 [Reference] | 1 [Reference] |
| Affected | 7124905 | 6990/344908 | 0.98  (0.96-1.00) | **1.53*****  (1.49-1.65) | **1.43*****  (1.40-1.47) | **1.58*****  (1.54-1.62) | **1.56*****  (1.52-1.60) |
| HR, Hazard ratio. Model 1 crude. Model 2 adjusted for sex, education and birth year. Model 3 adjusted additionally for COPD, alcoholism and obesity. M15, poly osteoarthritis; M16 osteoarthritis of the hip joint; M17 osteoarthritis of the knee joint; M18 osteoarthritis of the first carpometacarpal joint; M19 other types of osteoarthritis. * p<0.05, ** p<0.01, *** p<0.001 | | | | | | | |

**Supplementary Figures 1A.** Kaplan-Meier of M15, poly OA, free survival estimates by familial history.

| 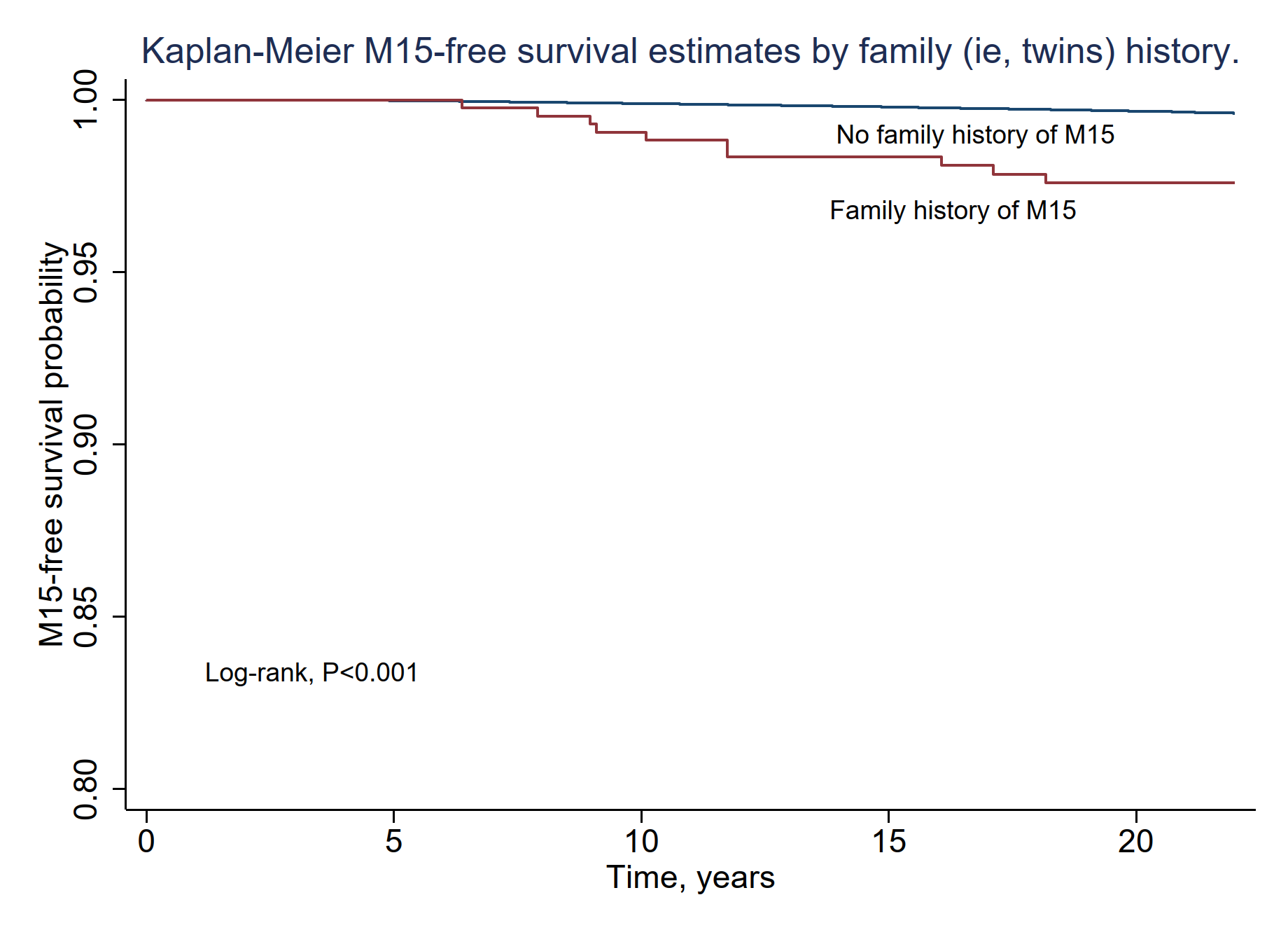 | 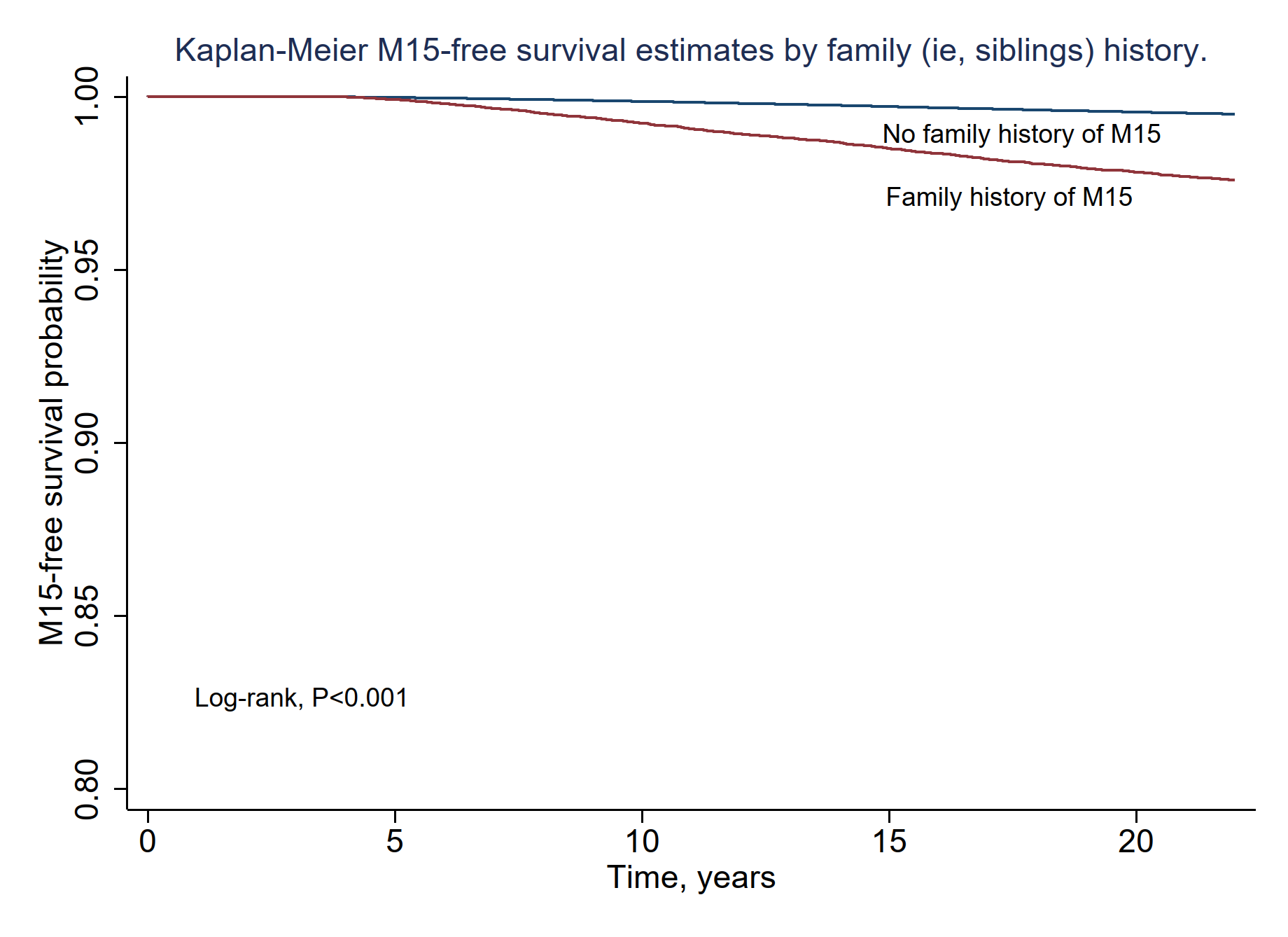 | 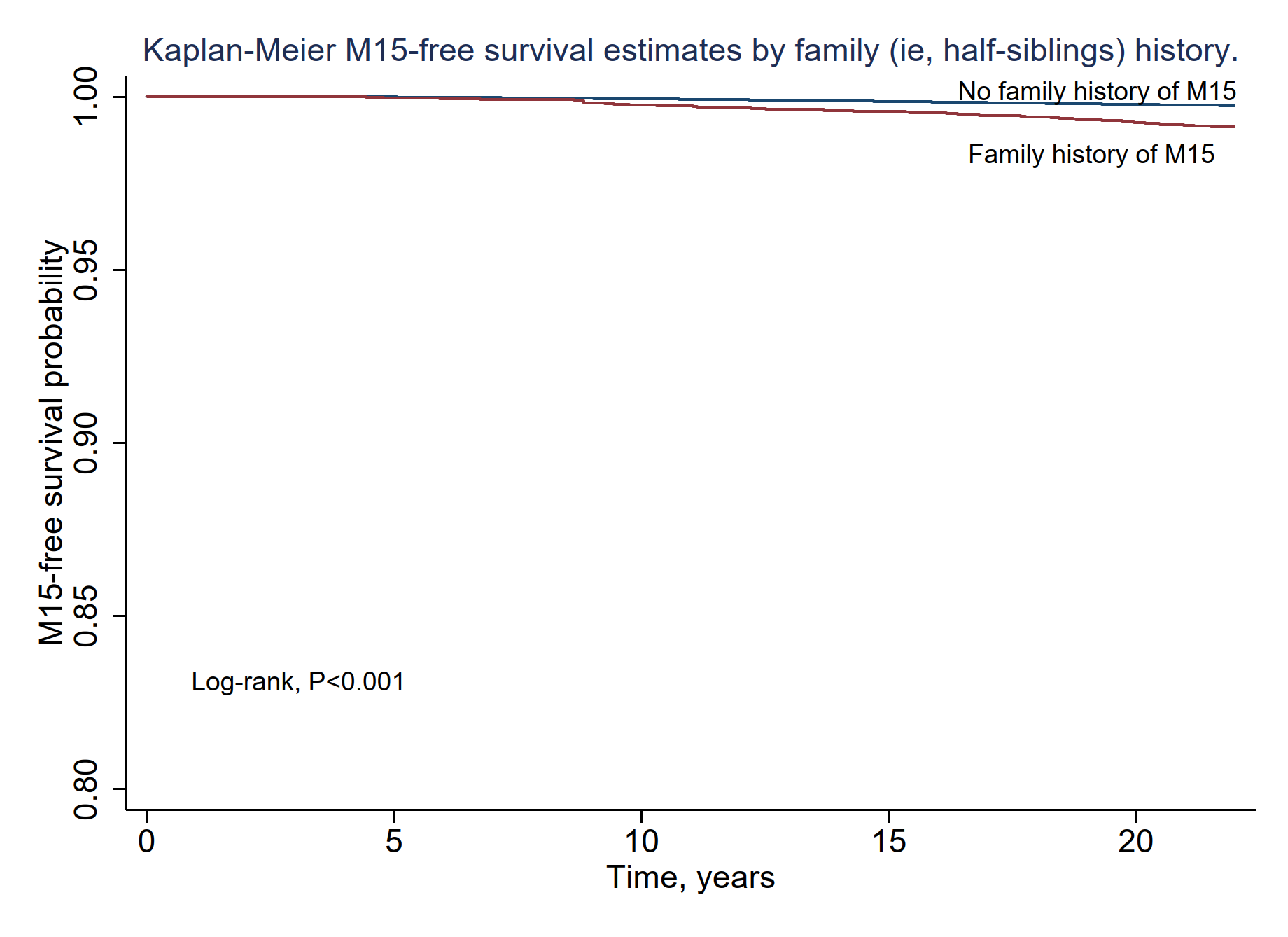 | 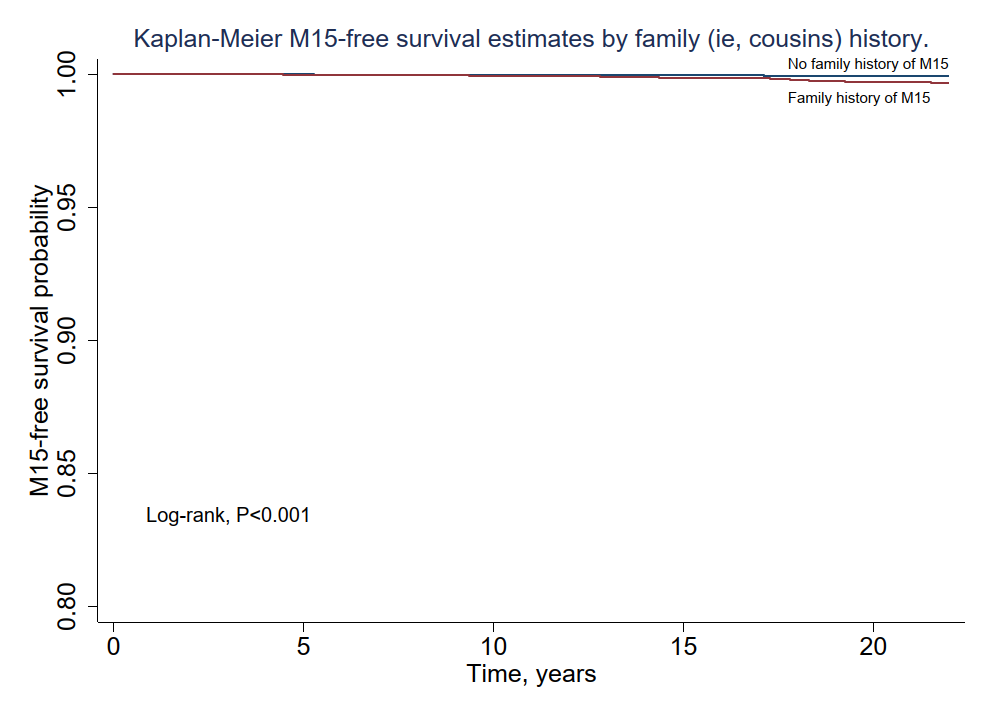 |
| --- | --- | --- | --- |

**Supplementary Figures 1B.** Kaplan-Meier M16, hip OA, free survival estimates by familial history.

| 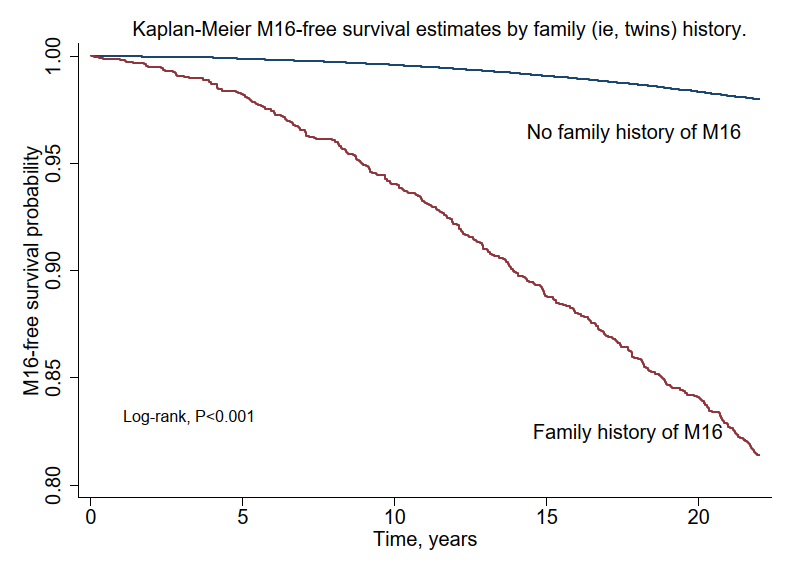 | 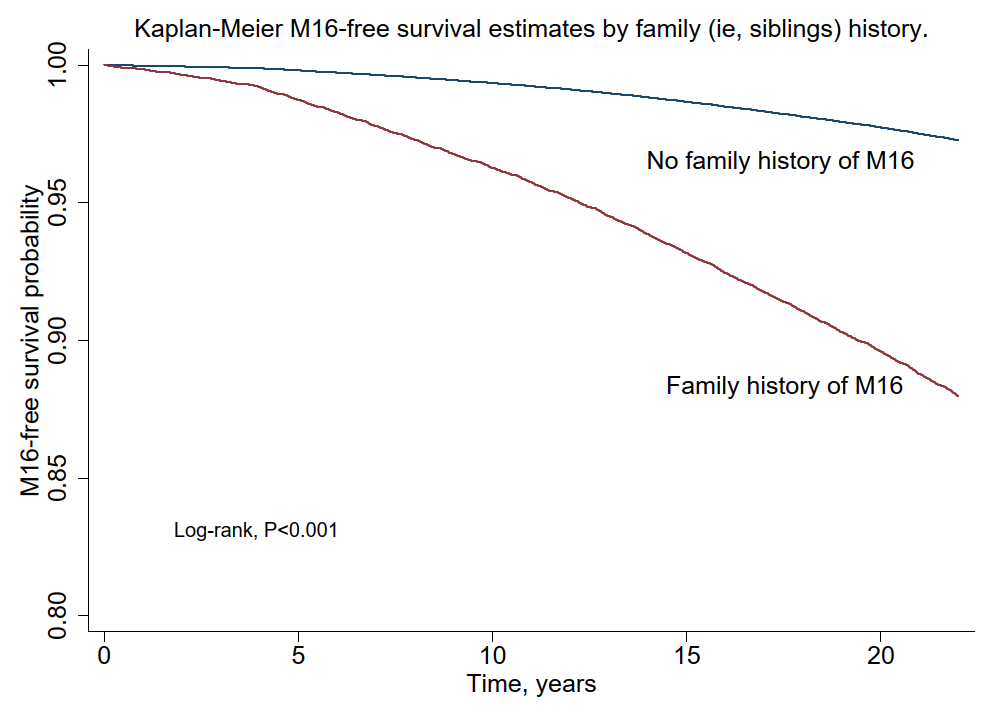 | 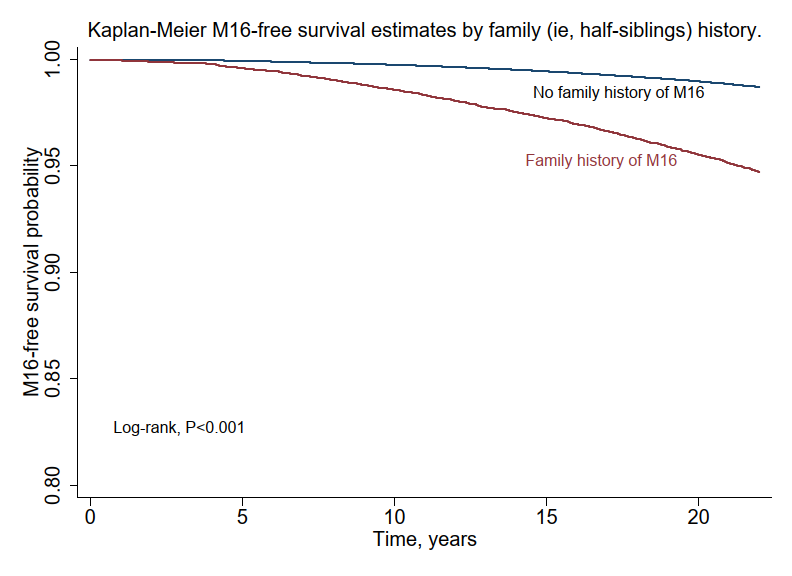 | 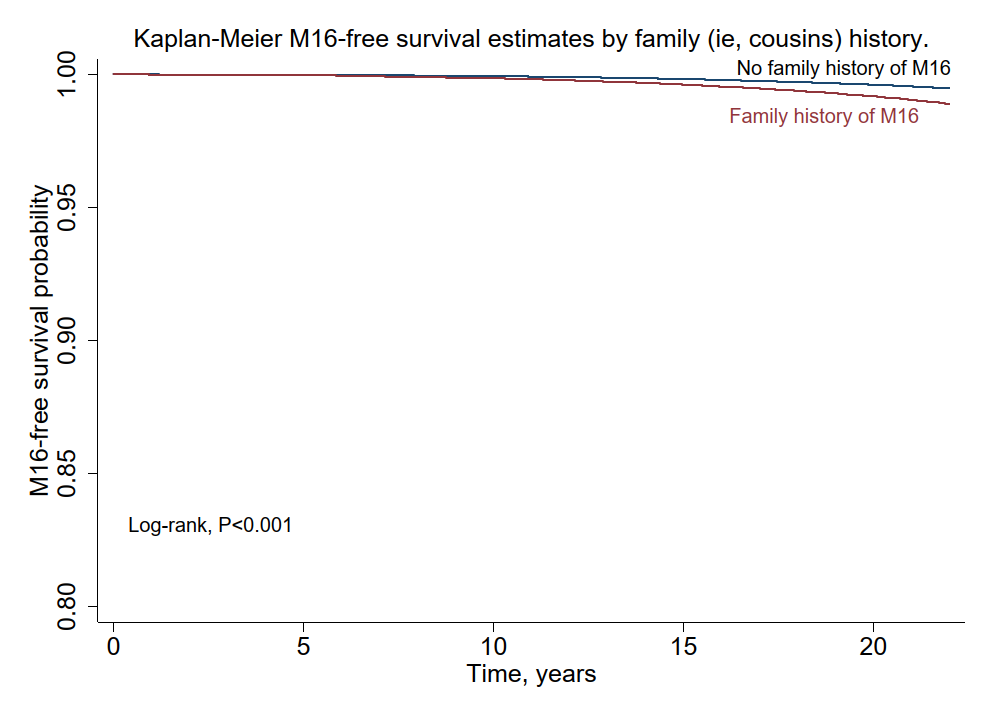 |
| --- | --- | --- | --- |

**Supplementary Figures 1C.** Kaplan-Meier M17, knee OA, free survival estimates by familial history.

| 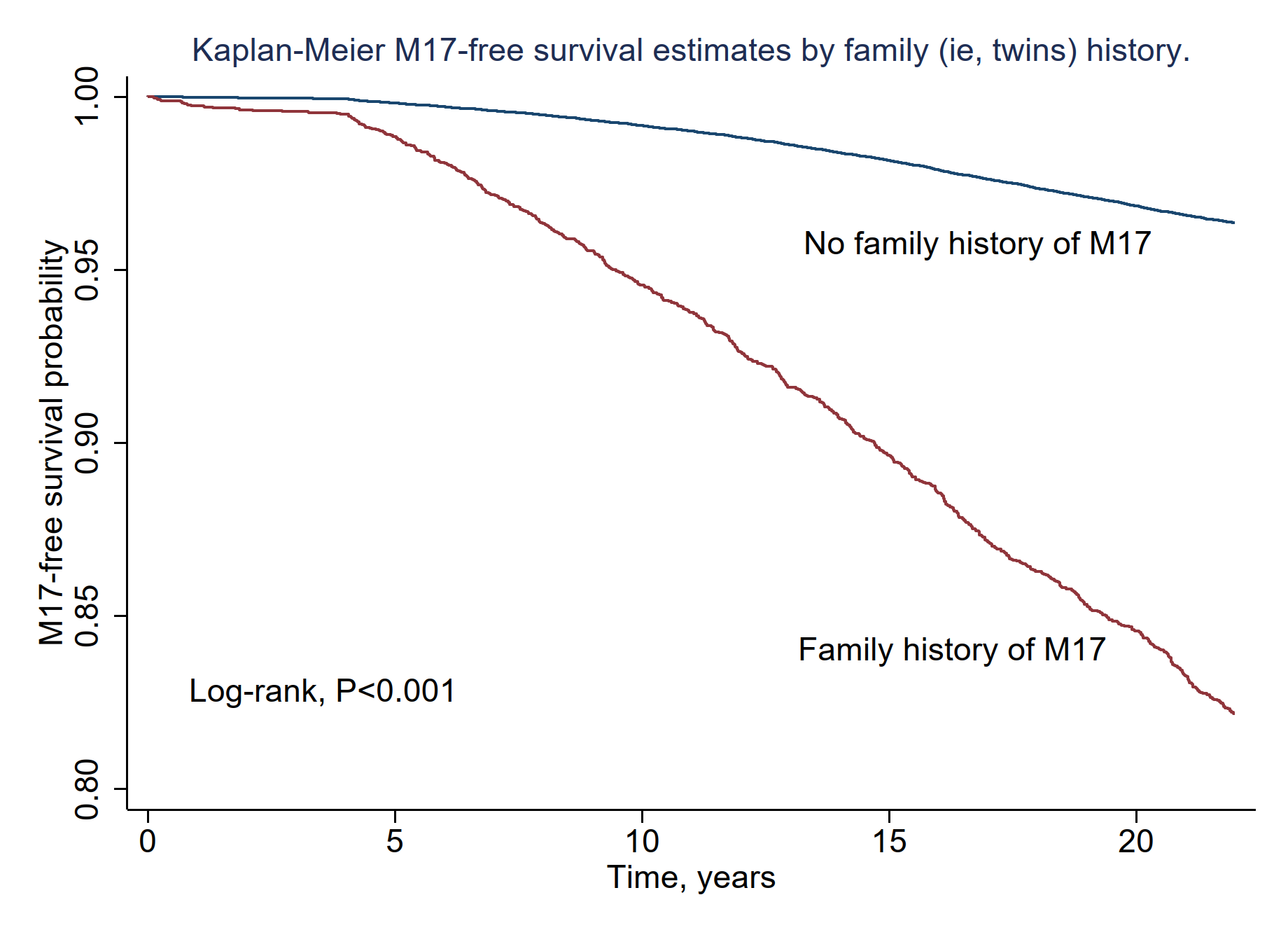 | 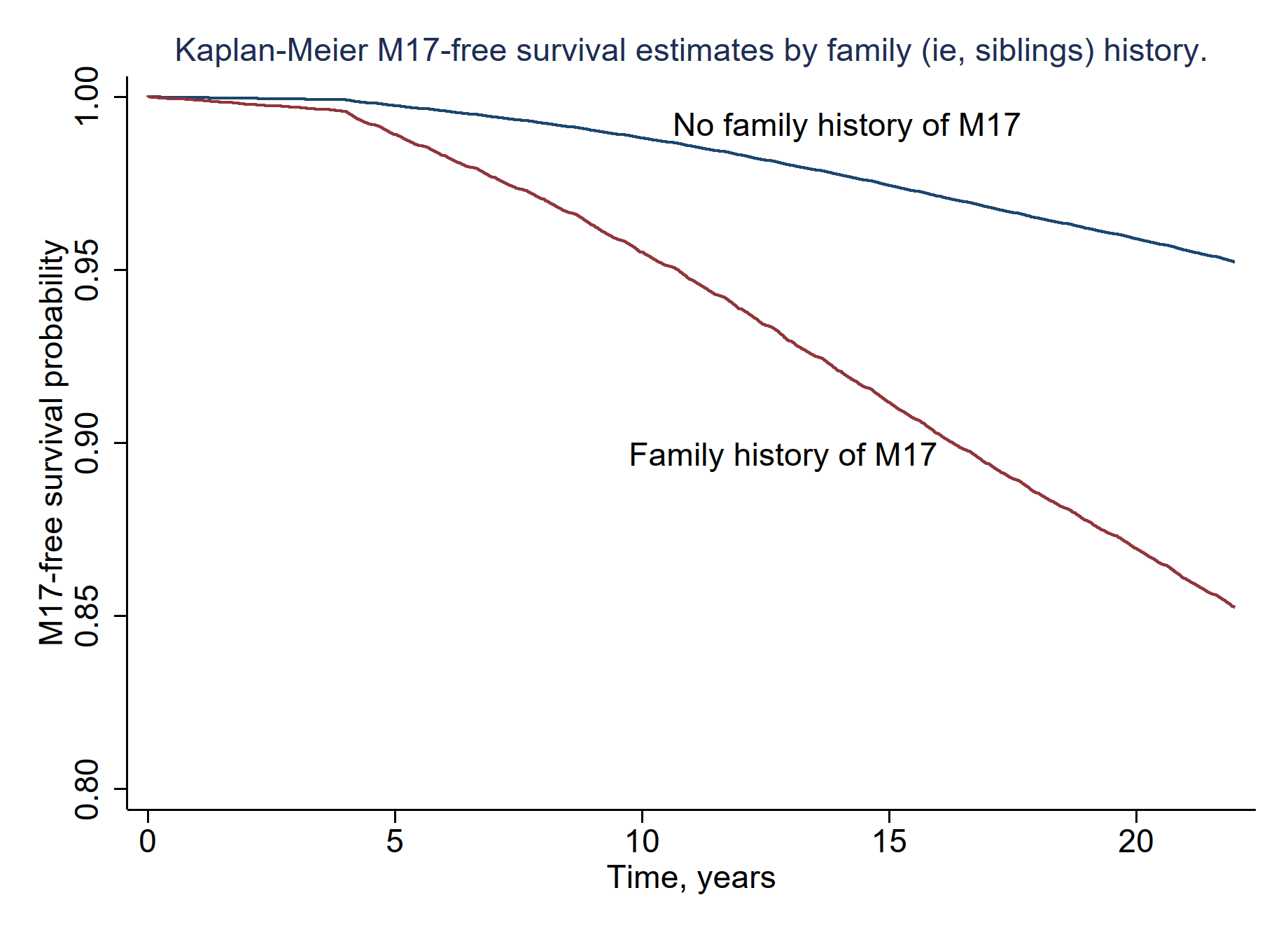 | 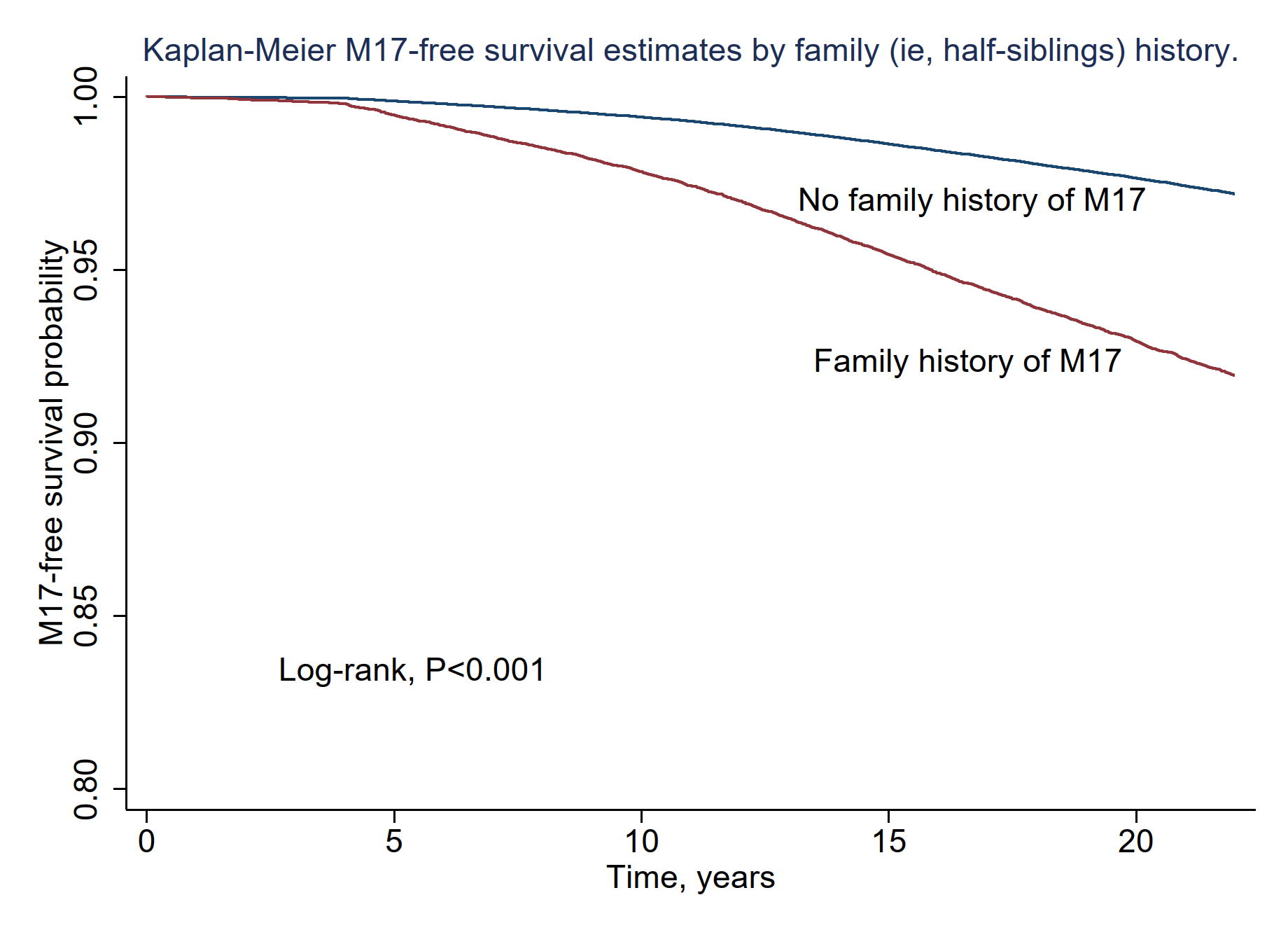 | 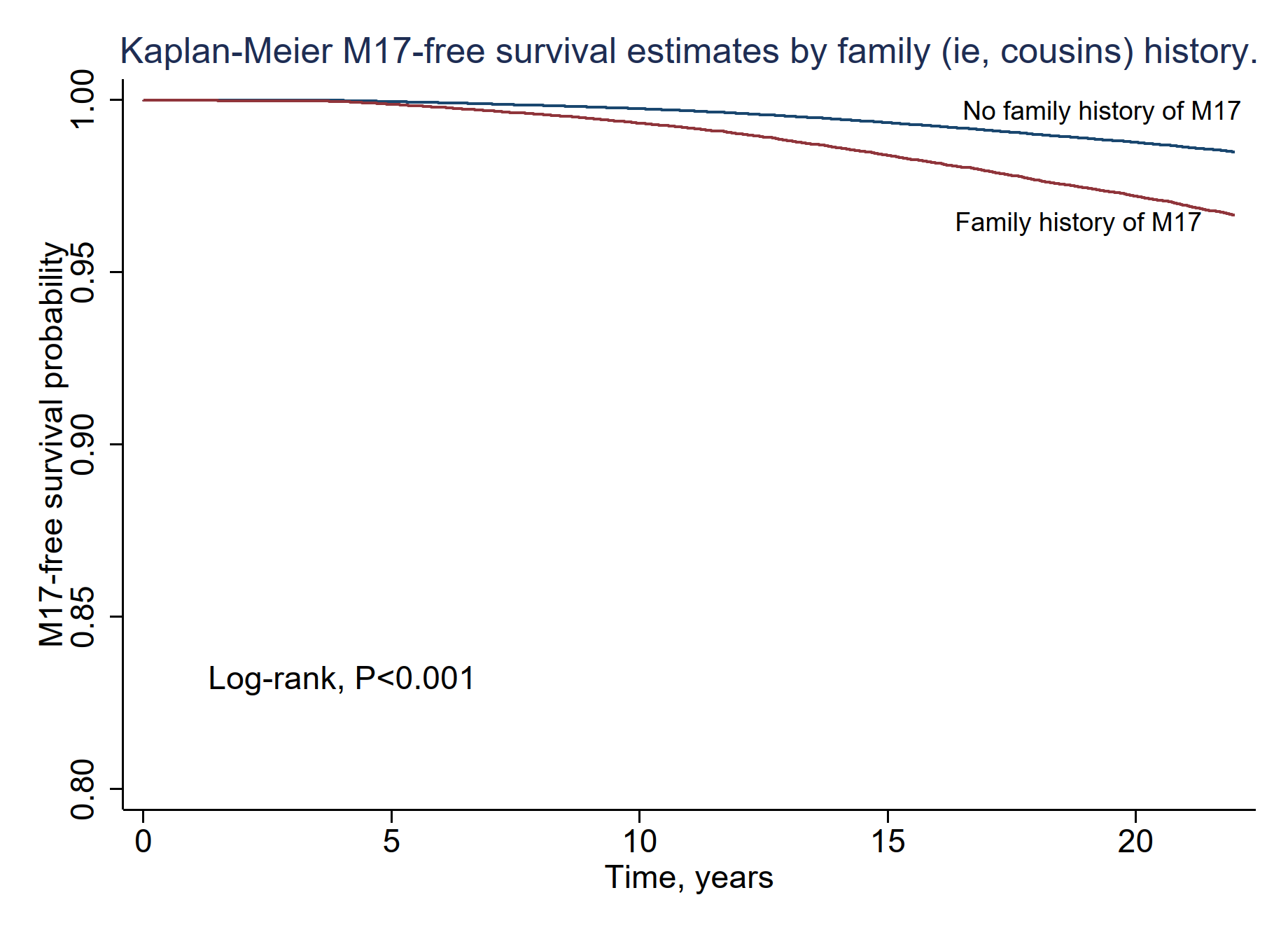 |
| --- | --- | --- | --- |

**Supplementary Figures 1D.** Kaplan-Meier M18, OA of the first carpometacarpal joint. free survival estimates by familial history.

| 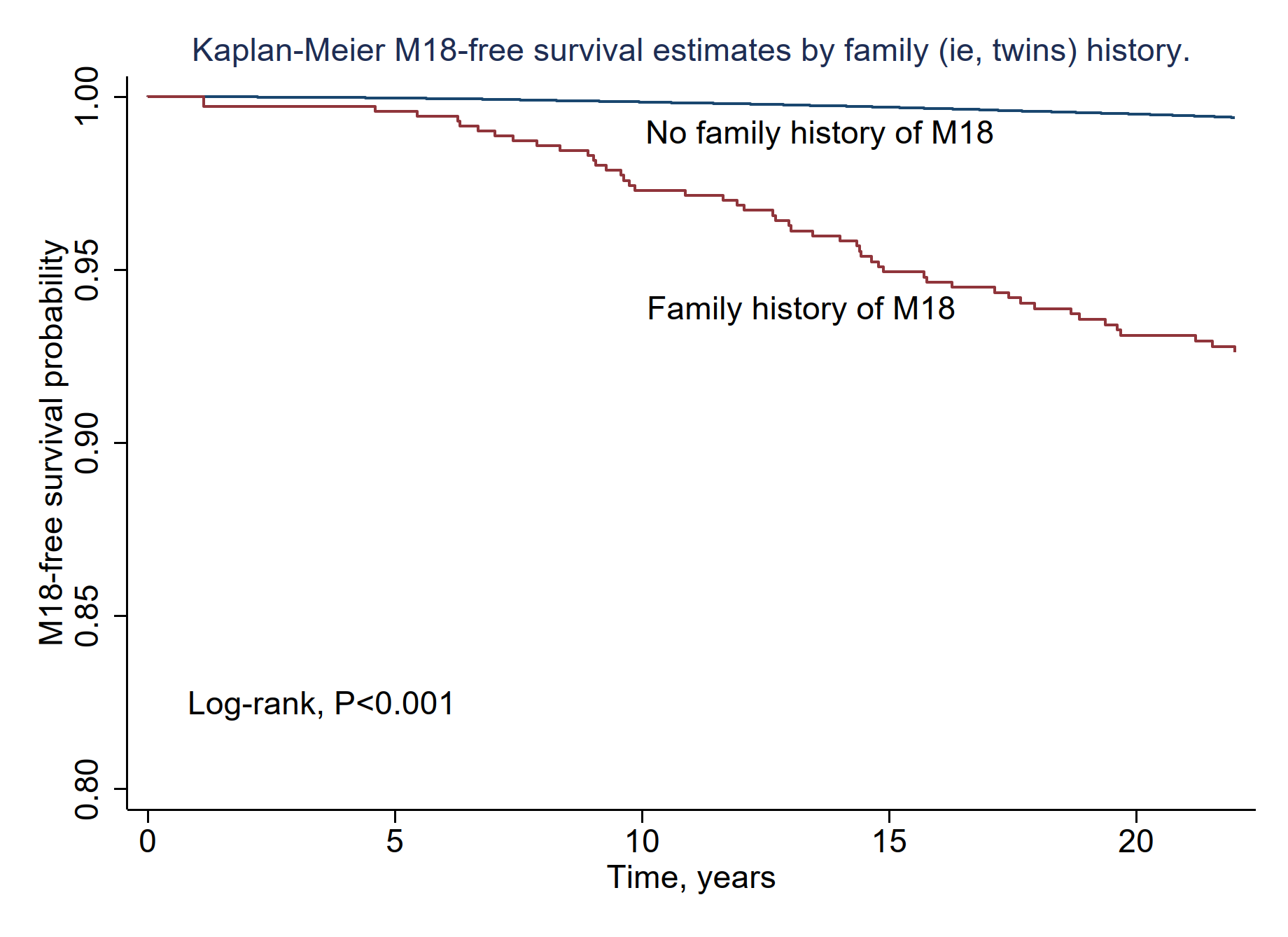 | 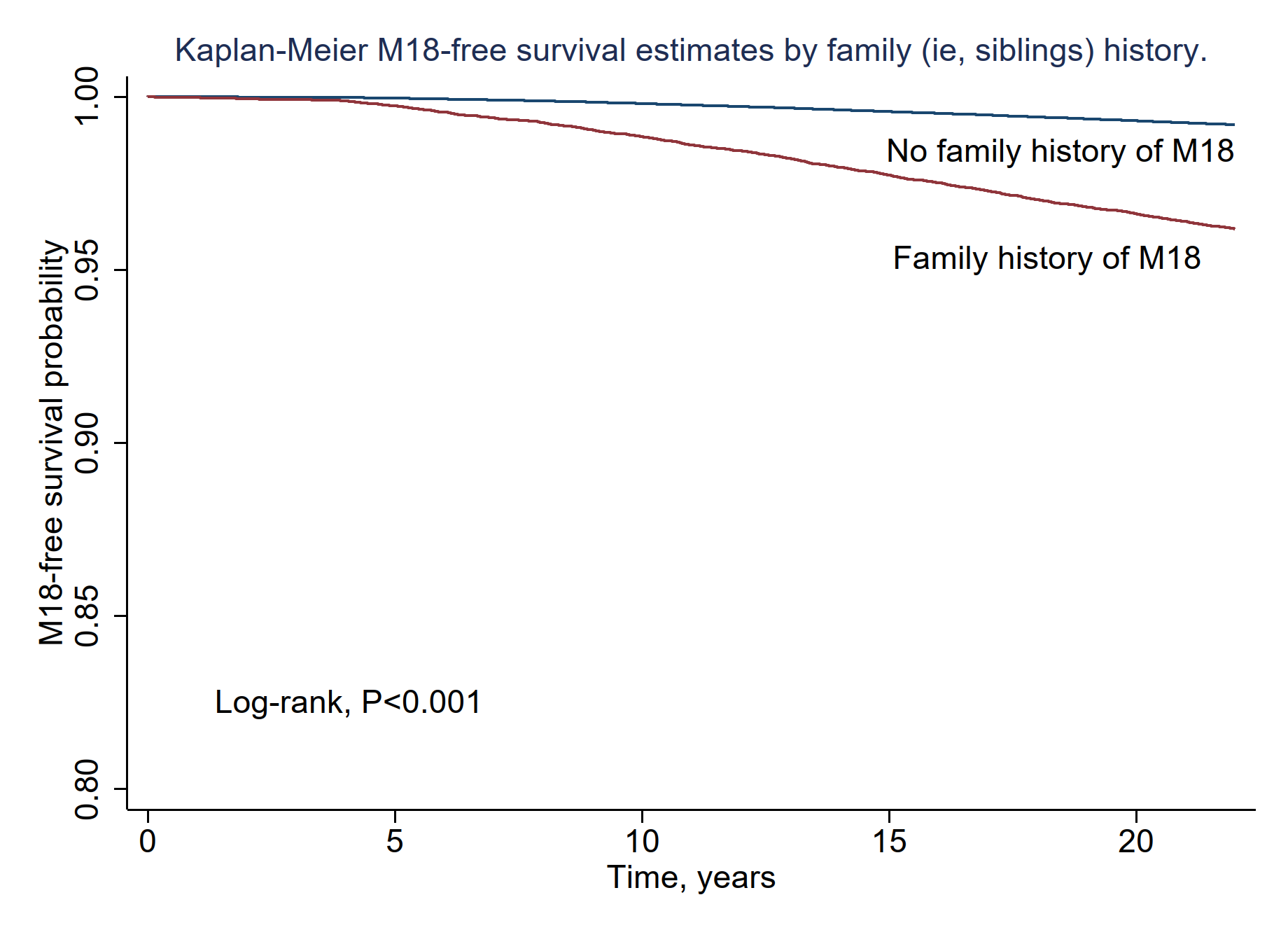 | 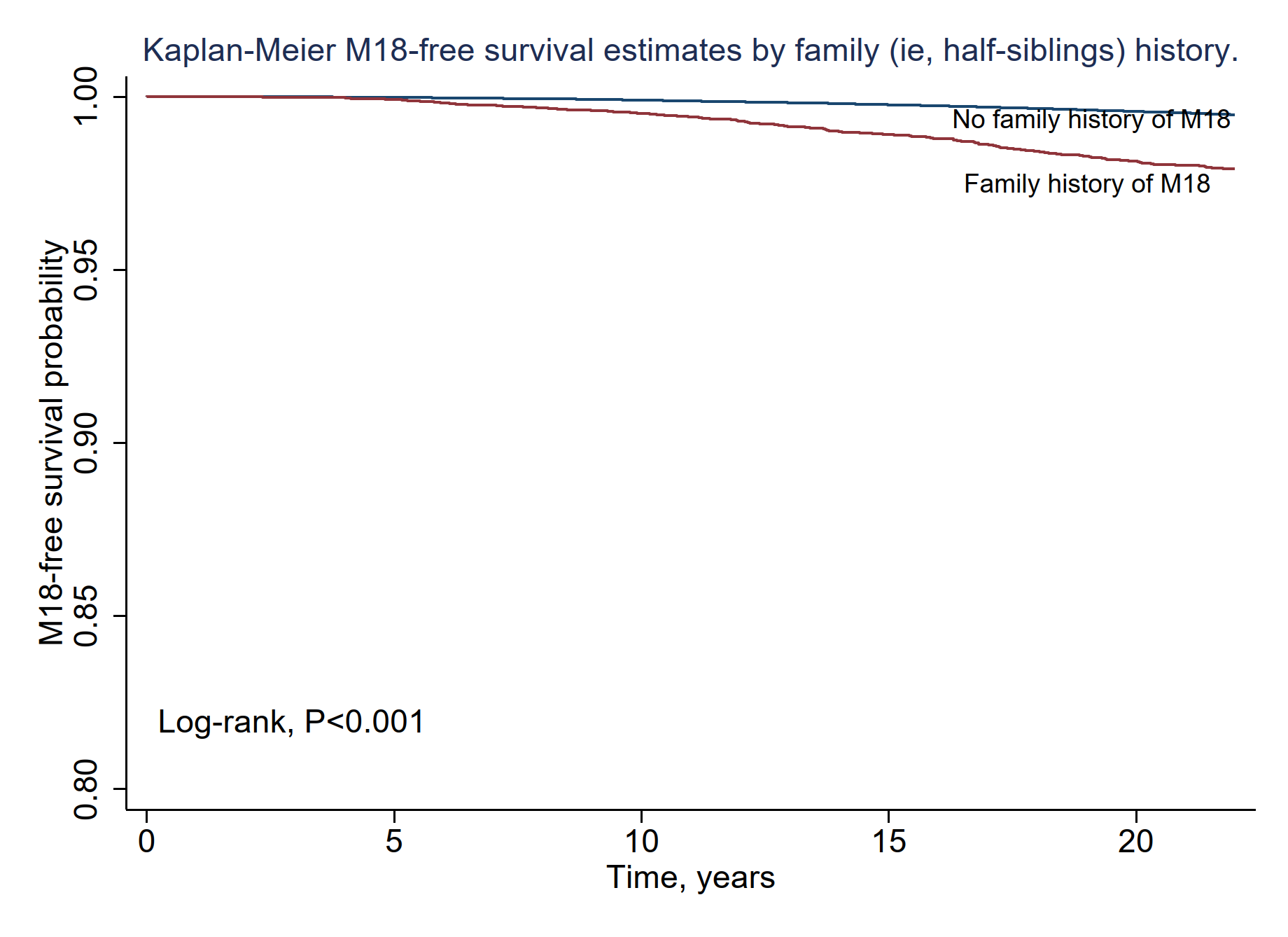 | 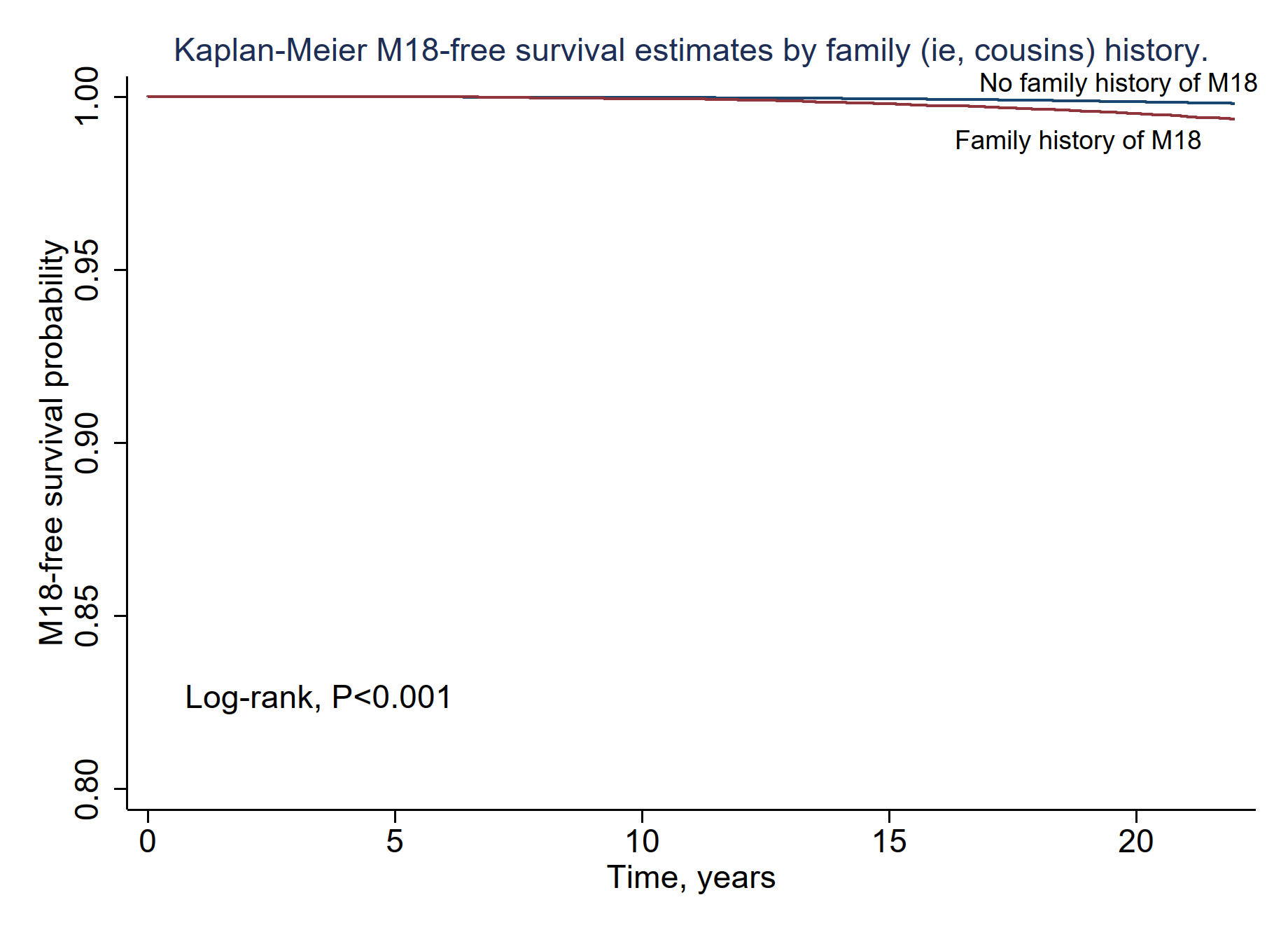 |
| --- | --- | --- | --- |

**Supplementary Figures 1E.** Kaplan-Meier of shoulder OA, M19 free survival estimates by familial history.

| 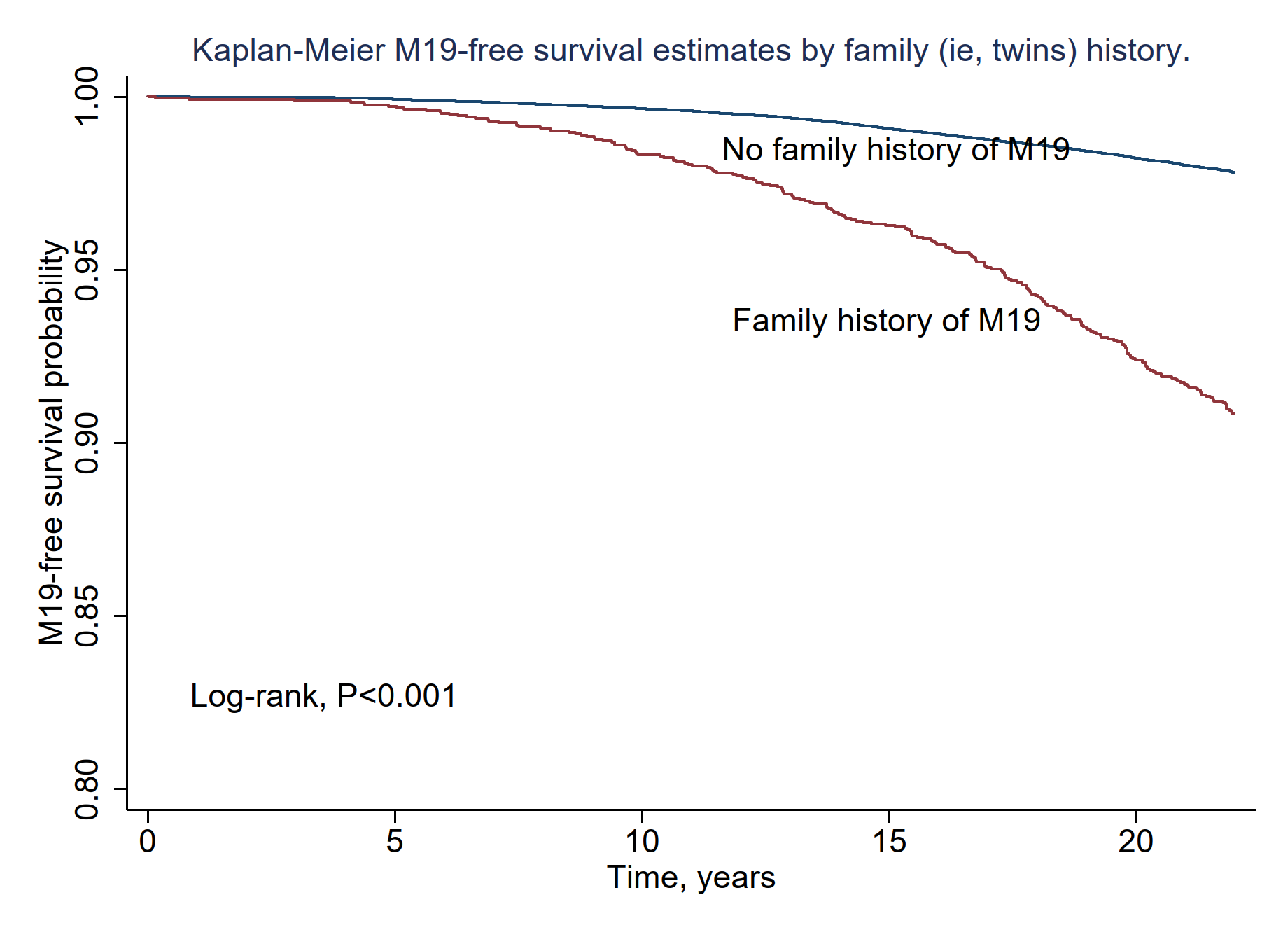 | 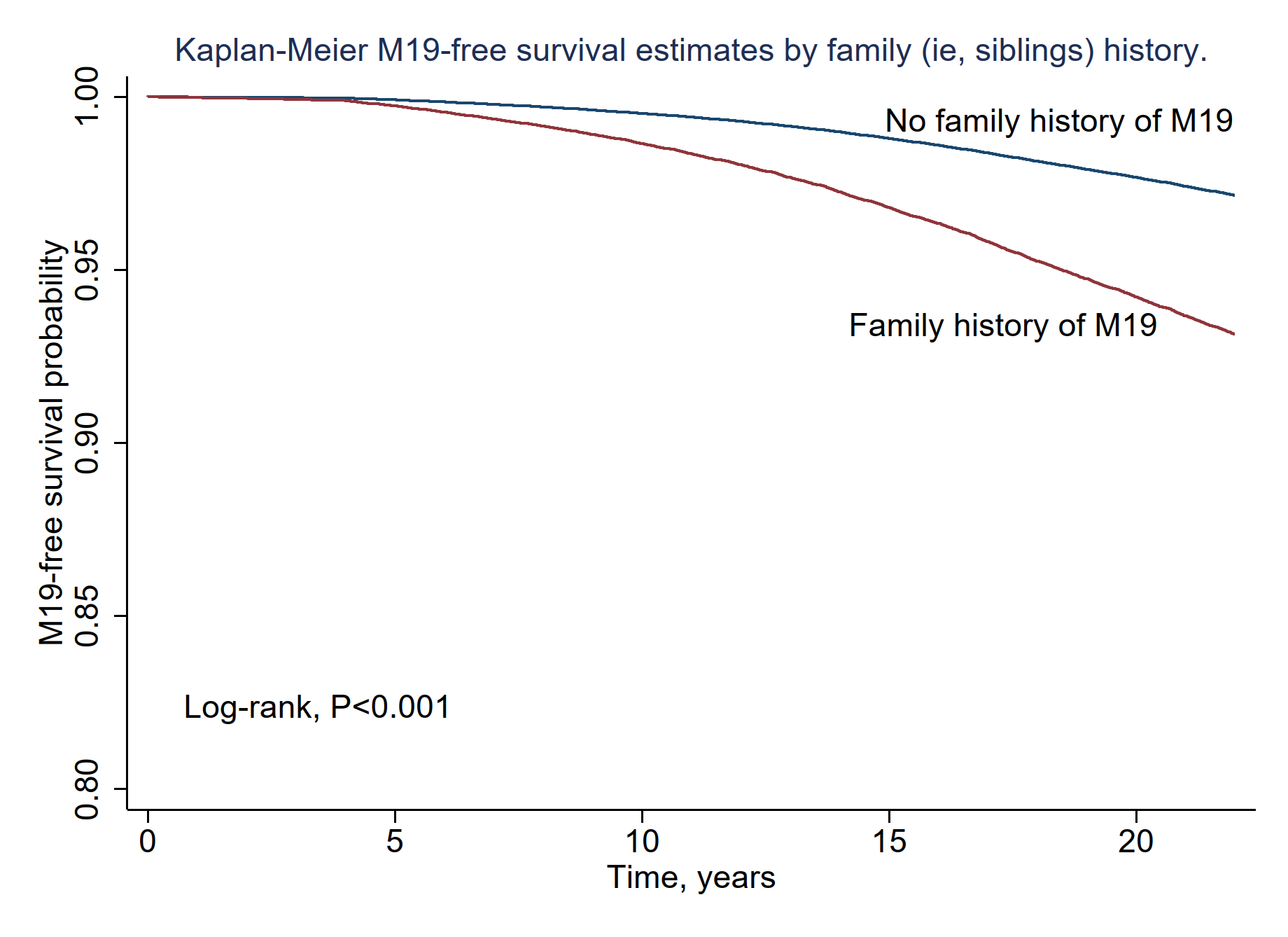 | 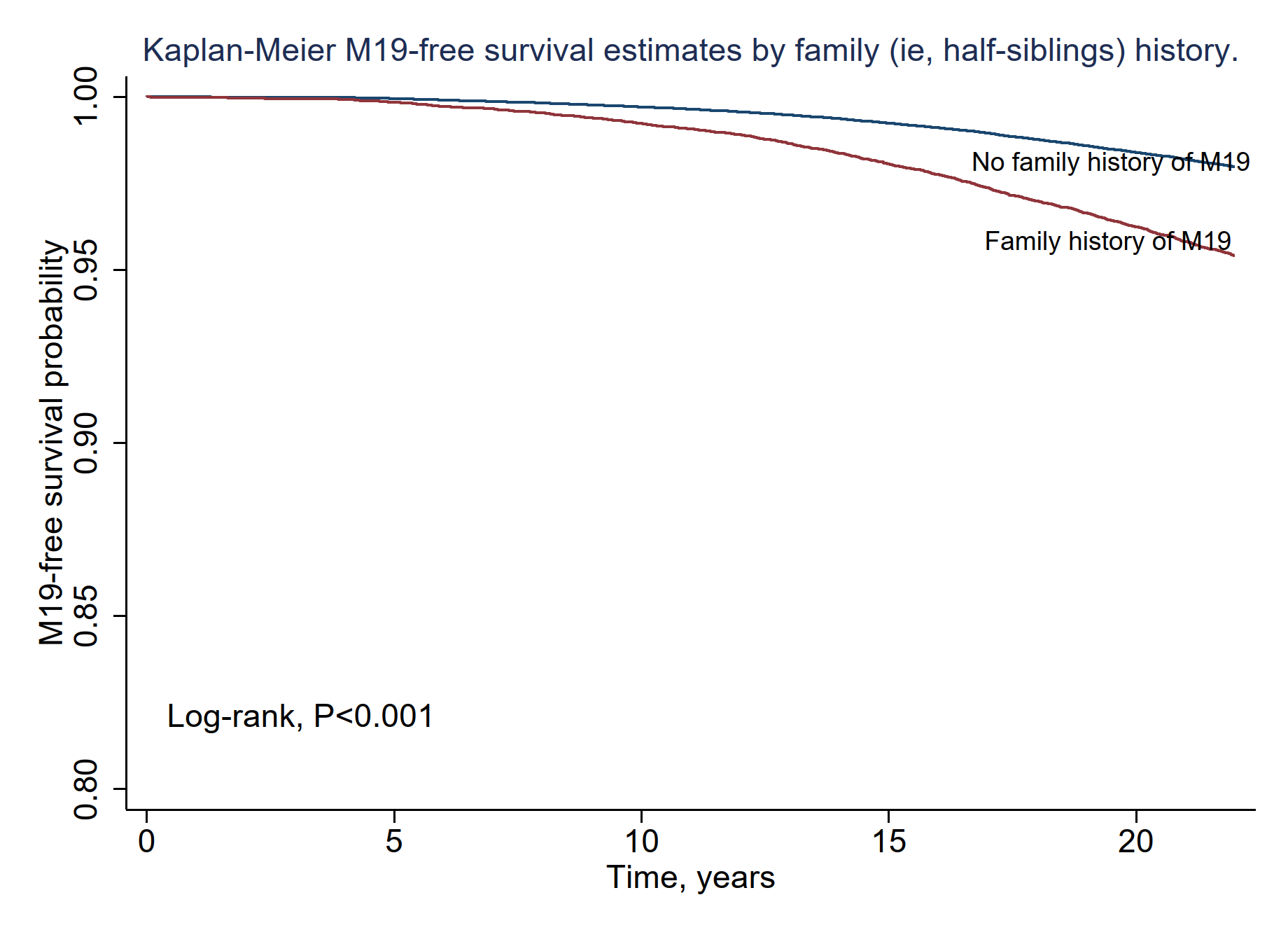 | 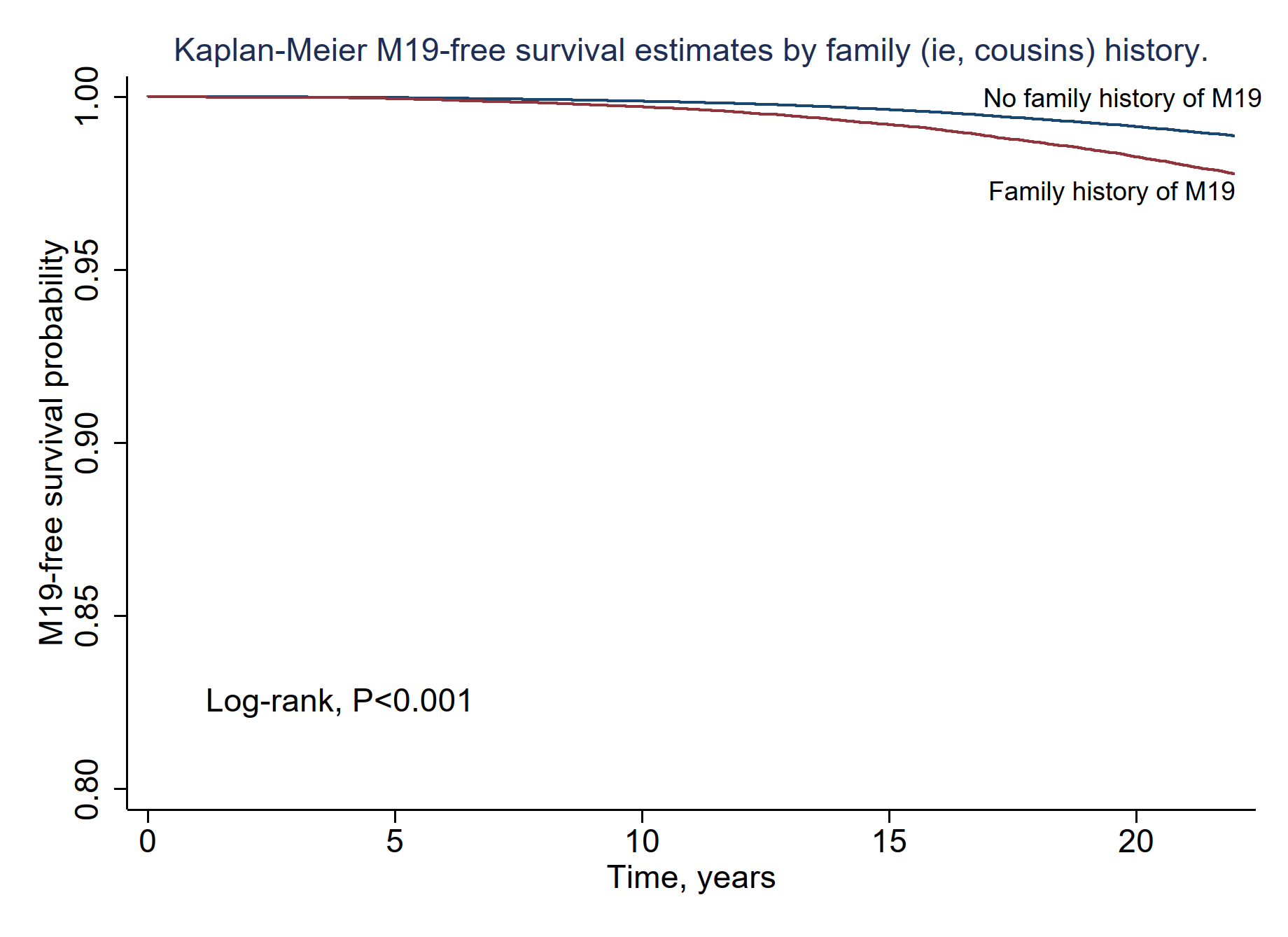 |
| --- | --- | --- | --- |

**Supplementary Figures 2A.** Cumulative incidence curves of poly OA, M15 estimates by familial history.

| 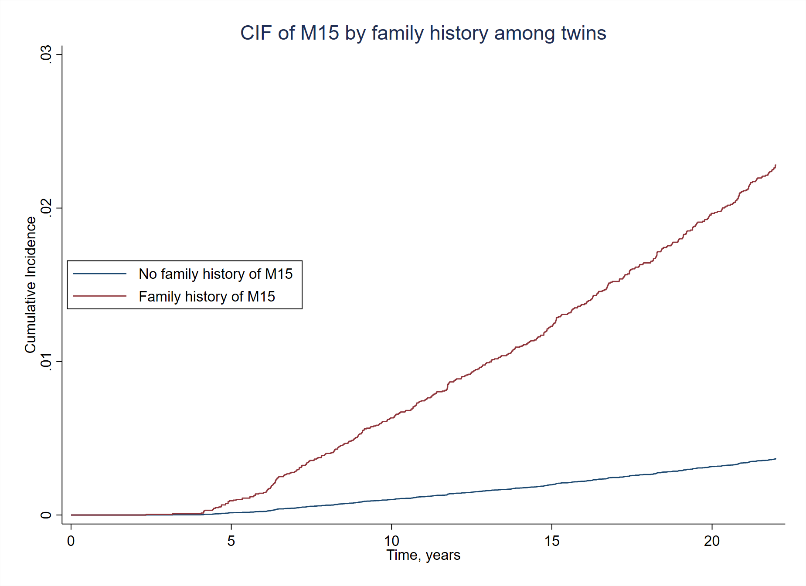 | 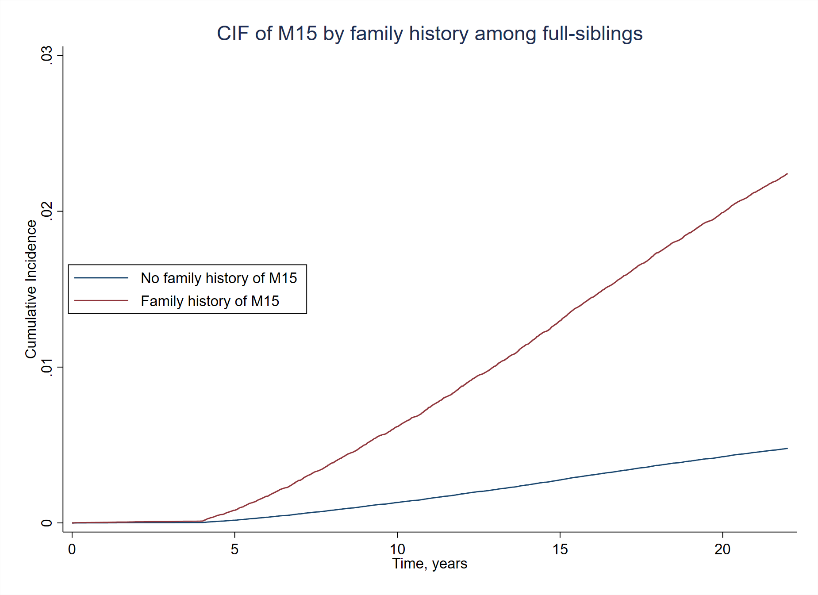 | 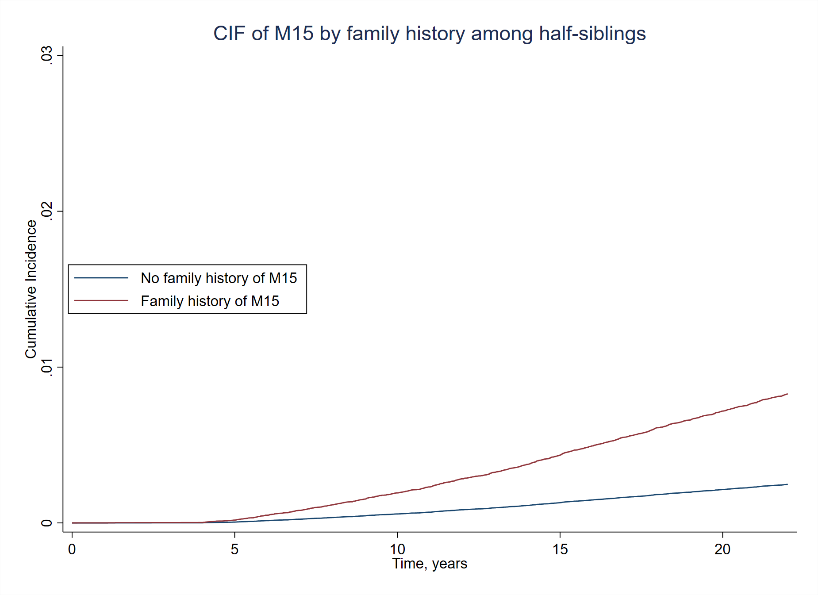 | 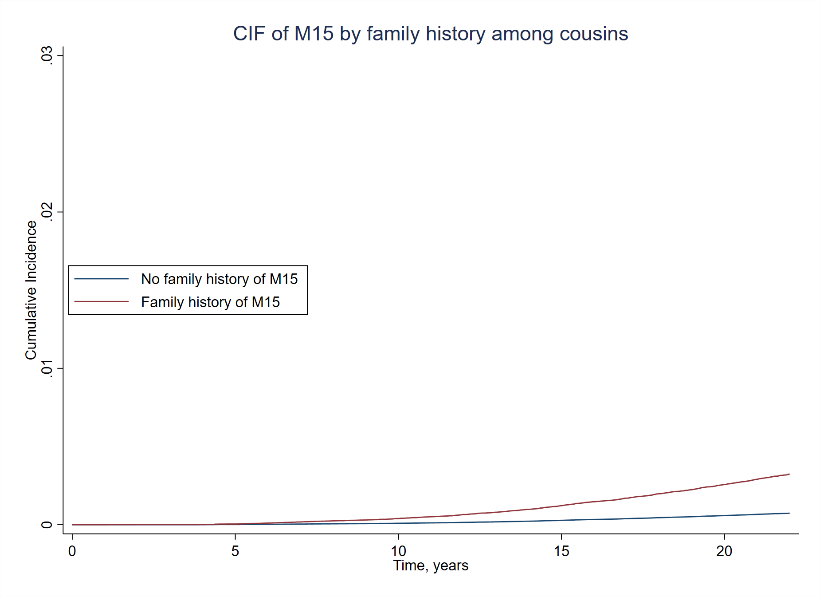 |
| --- | --- | --- | --- |

**Supplementary Figures 2B.** Cumulative incidence curves of hip OA, M16 estimates by familial history.

| 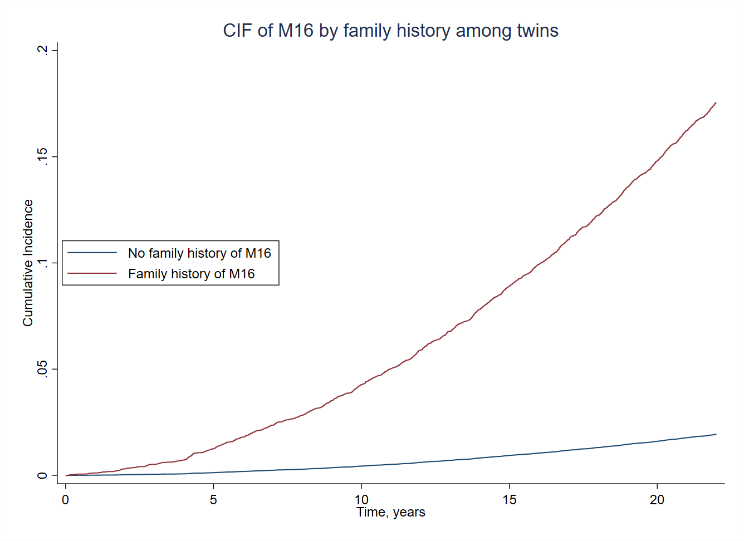 | 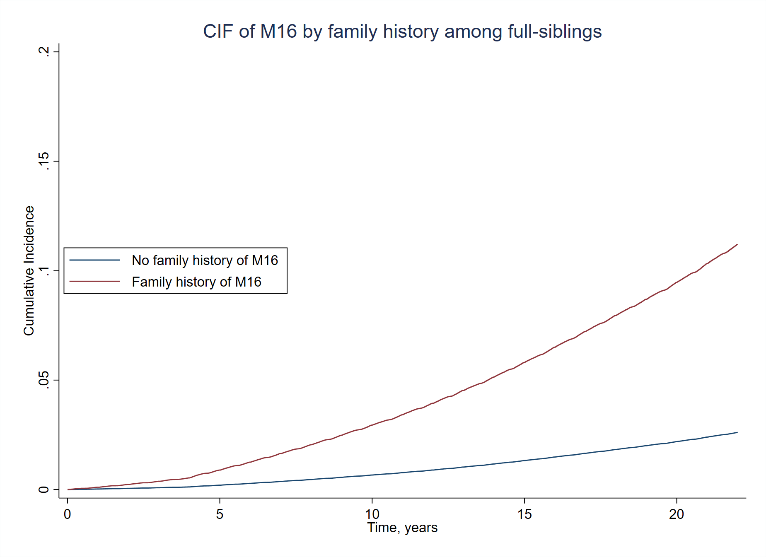 | 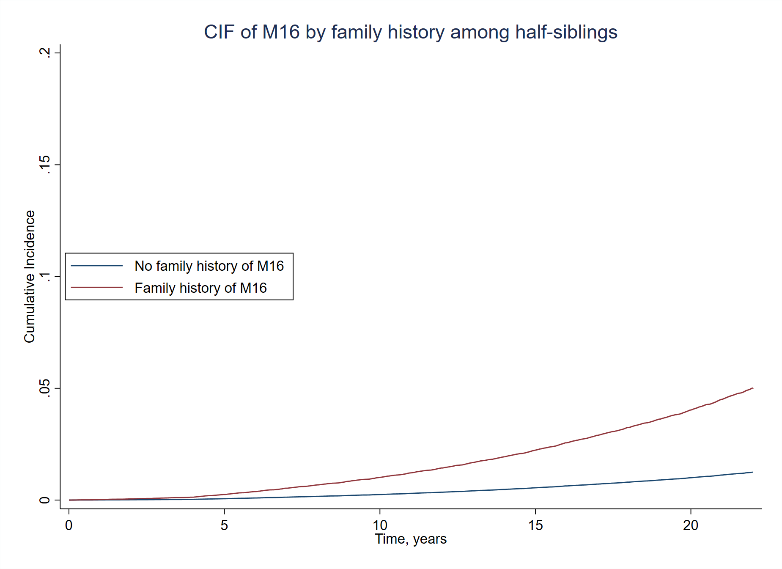 | 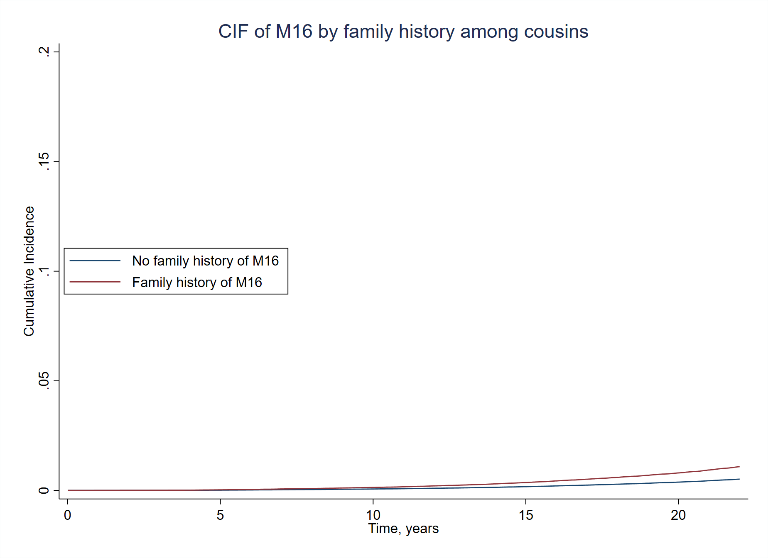 |
| --- | --- | --- | --- |

**Supplementary Figures 2C.** Cumulative incidence curves of knee OA, M17 estimates by familial history.

| 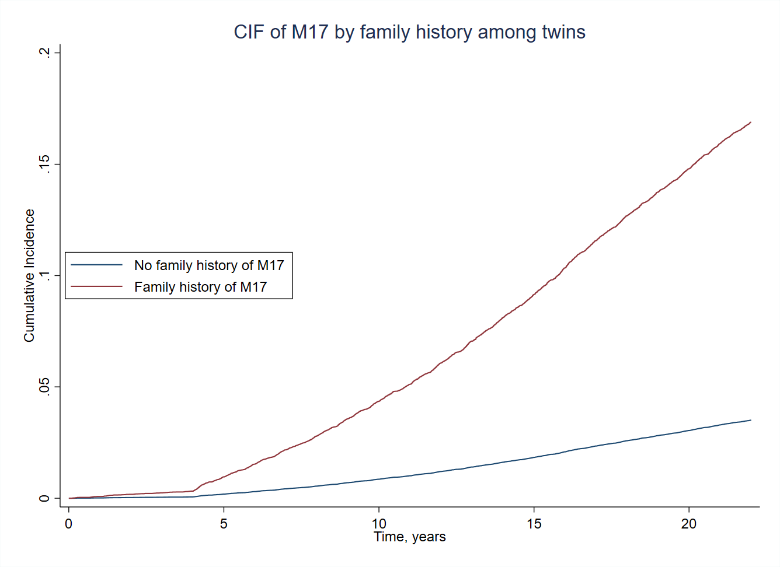 | 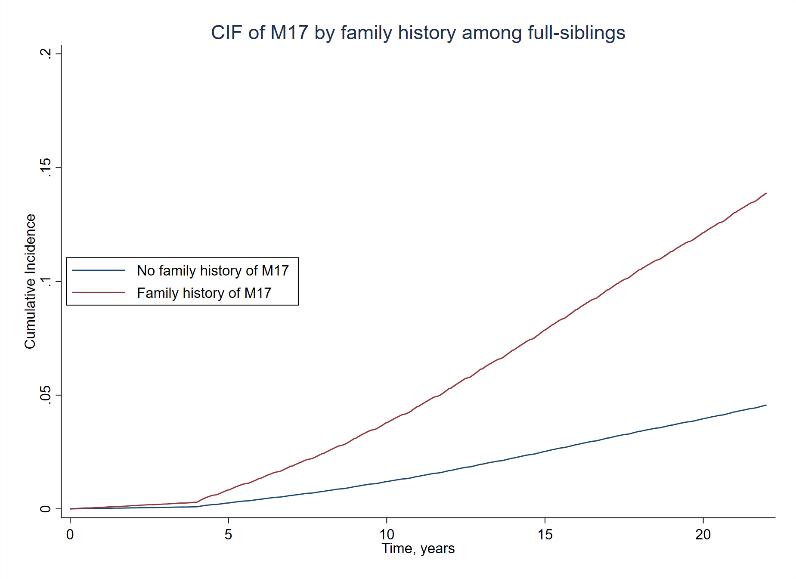 | 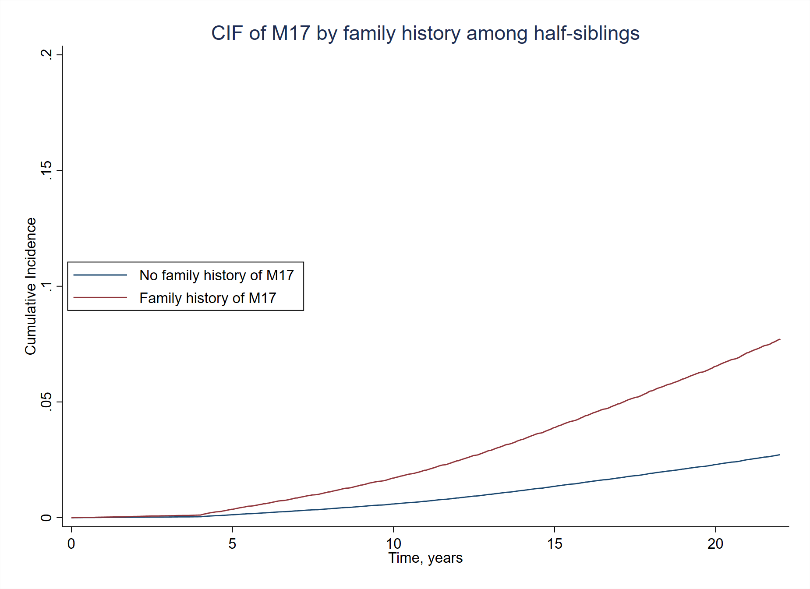 | 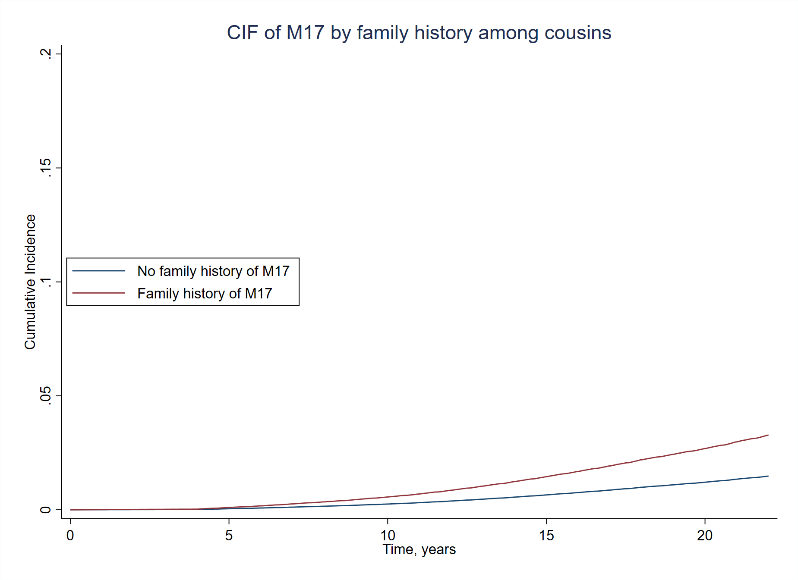 |
| --- | --- | --- | --- |

**Supplementary Figures 2D.** Cumulative incidence curves of OA of the first carpometacarpal joint, M18 estimates by familial history.

| 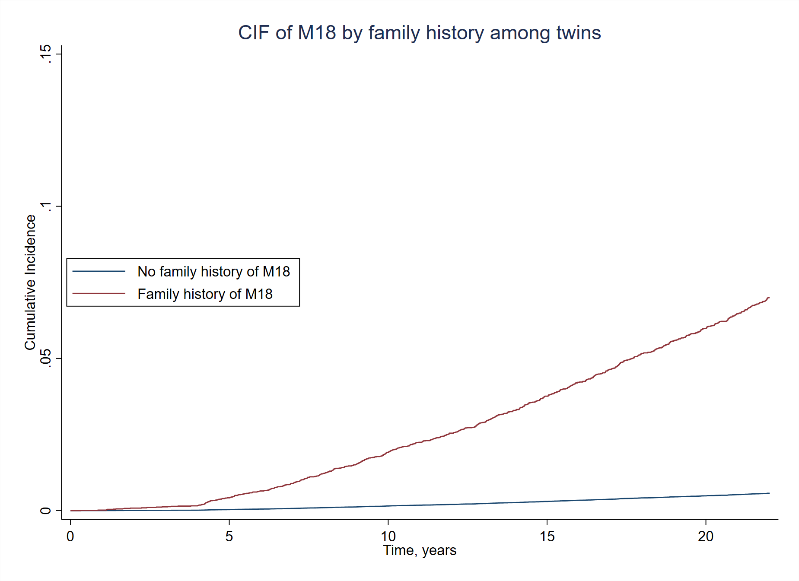 | 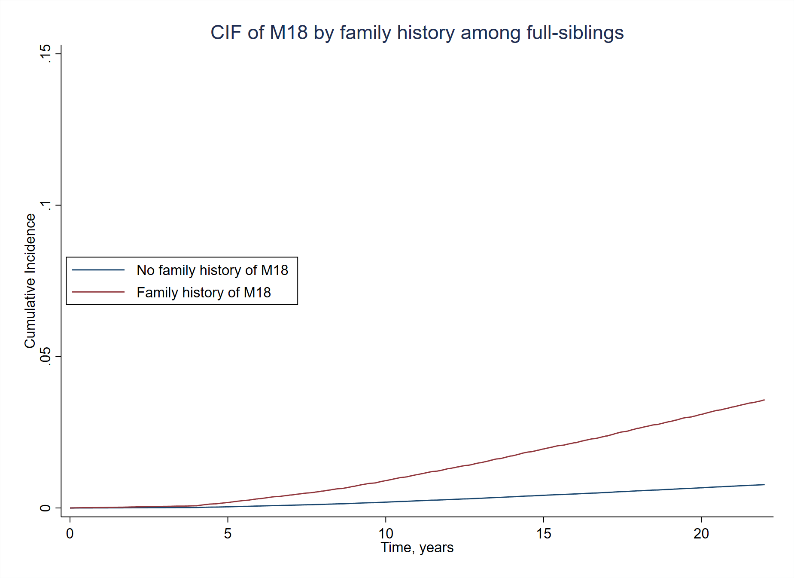 | 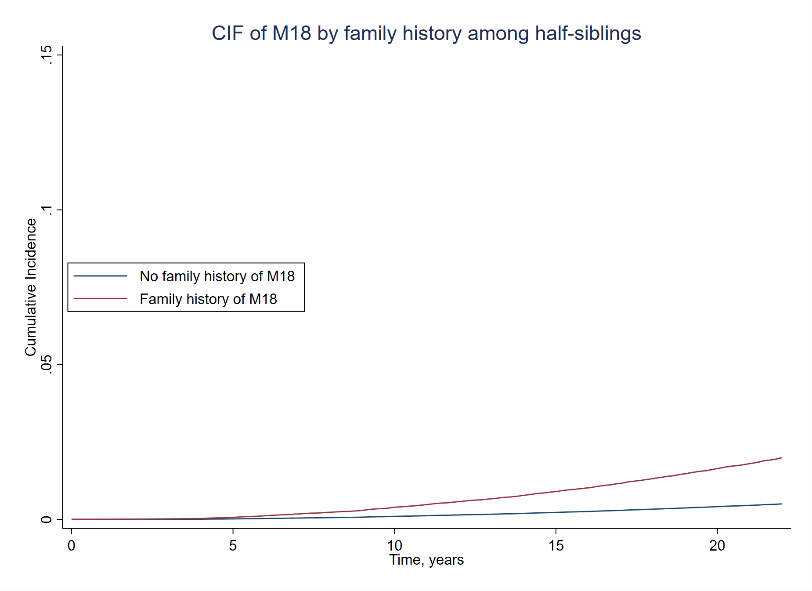 | 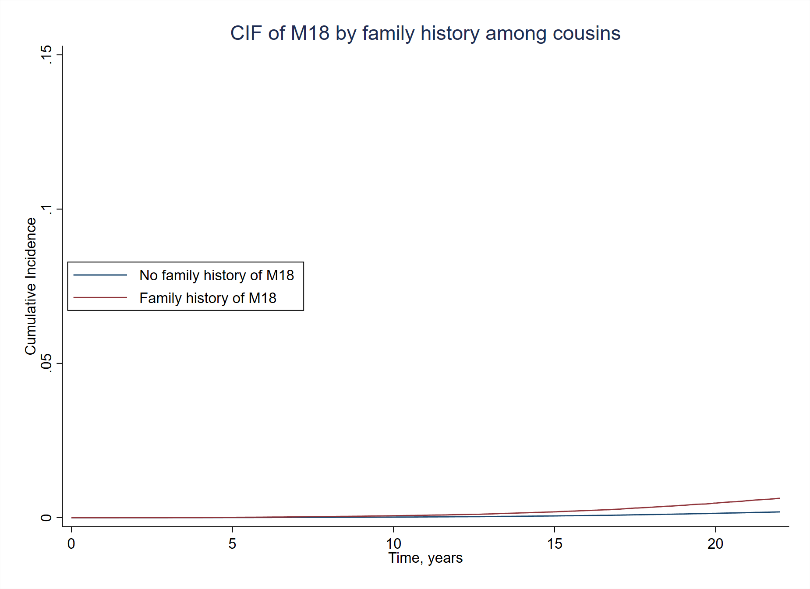 |
| --- | --- | --- | --- |

**Supplementary Figures 2E.** Cumulative incidence curves of other OA, M19 estimates by familial history.

| 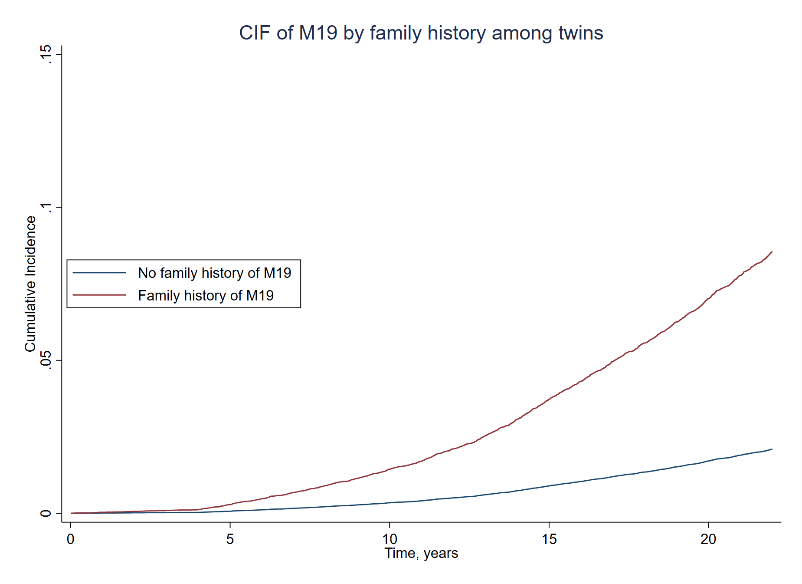 | 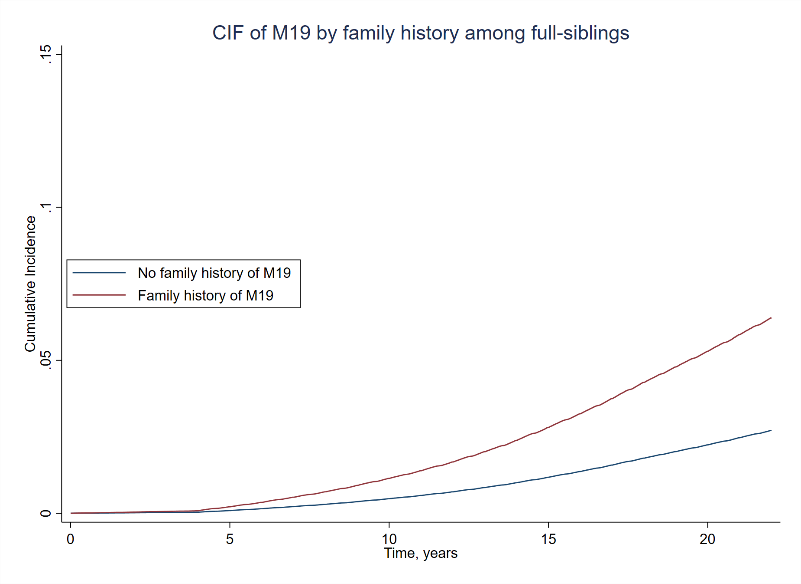 | 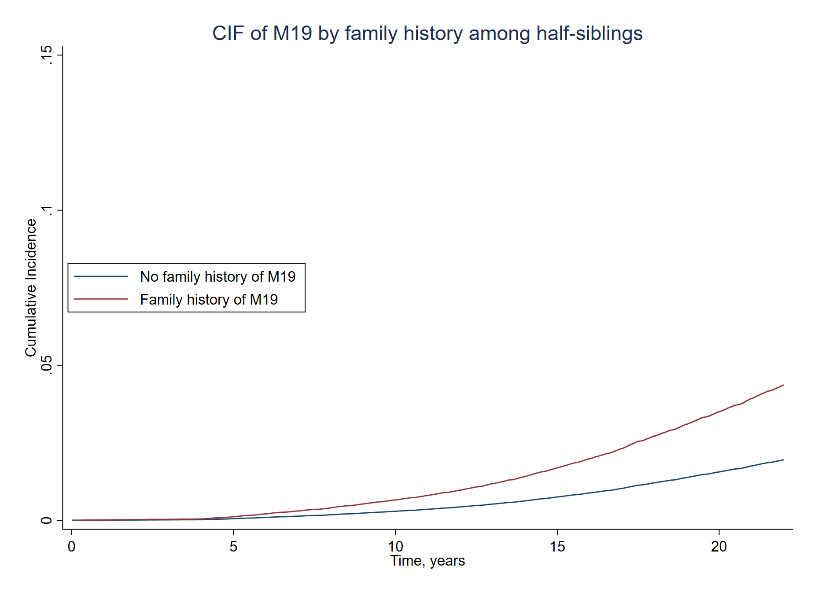 | 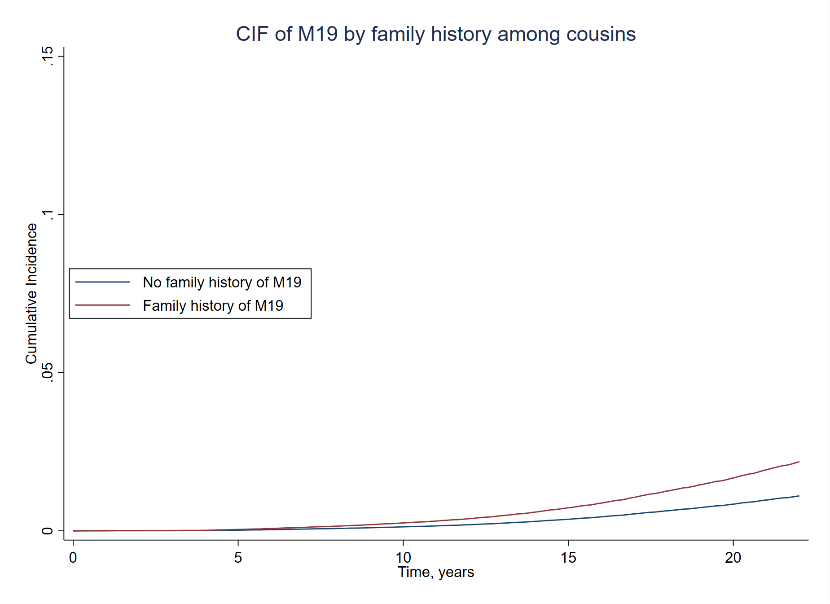 |
| --- | --- | --- | --- |
